# Sexual dimorphism in the association of circulating TAM receptors–ligands with MASLD-related fibrosis

**DOI:** 10.1016/j.jhepr.2026.101937

**Published:** 2026-06-30

**Authors:** Yiwei Zhu, Neha Gupta, Bruno Cogliati, Yulu He, Asher Leviton, Jihane N. Benhammou, Steven-Huy B. Han, Zhenqi Zhou, Alexander Nguyen, Xiaotao Zhang, Rajat Singh, Scott L. Friedman, Meena B. Bansal, Bishuang Cai

**Affiliations:** 1 Vatche and Tamar Manoukian Division of Digestive Diseases, Department of Medicine, David Geffen School of Medicine, University of California, Los Angeles, CA, USA; 2 Comprehensive Liver Research Center at UCLA, University of California, Los Angeles, CA, USA; 3 Division of Liver Diseases, Department of Medicine, Icahn School of Medicine at Mount Sinai, New York, NY, USA; 4 Division of Endocrinology, Diabetes and Hypertension, Department of Medicine, David Geffen School of Medicine, University of California, Los Angeles, CA, USA

**Keywords:** MASH, MerTK, Sex differences, Menopause, Estrogen

## Abstract

**Background & Aims:**

The TAM receptors (MerTK and Axl) and their ligands (Gas6 and Protein S) have been implicated in MASLD-related fibrosis. However, whether circulating levels are associated with fibrosis stage and whether these associations differ by sex remain unclear. This study aimed to characterize circulating TAM receptors and ligands across fibrosis stages in men and women with MASLD.

**Methods:**

Circulating TAM receptors and ligands were measured in men (n = 75) and women (n = 78) with MASLD and analyzed for associations with fibrosis stage. Time-course choline-deficient, amino acid-defined, high-fat diet mouse models were established in male and female mice to assess circulating TAM members. These markers were further compared between premenopausal and postmenopausal women with MASLD. In addition, circulating soluble MerTK (sol-MerTK) was measured in mice treated with 17β-estradiol. *In vitro*, macrophages were treated with 17β-estradiol, and sol-MerTK and Mertk mRNA levels were measured.

**Results:**

In men with MASLD, circulating sol-MerTK was inversely associated with fibrosis stage (adjusted ordinal regression: odds ratio 0.63; 95% CI 0.51–0.78; *p* <0.0001) and decreased with fibrosis progression in male mice. In contrast, sol-MerTK showed no significant association with fibrosis stage in women (odds ratio 1.12; 95% CI 1.00–1.25; *p* = 0.0543) or female mice. Higher circulating sol-Axl and Gas6 levels were associated with more advanced fibrosis in both men and women with MASLD. Circulating sol-MerTK levels were higher in premenopausal than postmenopausal women (*p* = 0.0306). Treatment with 17β-estradiol increased circulating sol-MerTK levels in mice (*p* = 0.0119). In macrophages, 17β-estradiol increased sol-MerTK release in a dose-dependent manner (*p* <0.05), with a non-significant trend toward reduced *Mertk* mRNA expression (*p* >0.05).

**Conclusions:**

Circulating TAM receptors and ligands were associated with fibrosis stage in MASLD, with sex-specific associations observed for sol-MerTK. Furthermore, 17β-estradiol promoted MerTK cleavage, providing a potential mechanism underlying these sex differences.

**Clinical trial number:**

N/A.

**Impact and implications:**

MASLD-related fibrosis is progressive and sexually dimorphic. Our findings reveal sex-specific associations of circulating sol-MerTK and Protein S with liver fibrosis stages in MASLD, highlighting the need to incorporate sex as a biological variable in MerTK- and Protein S–related studies. In contrast, circulating sol-Axl and Gas6 were positively associated with liver fibrosis stages in both sexes, suggesting their potential as sex-independent indicators of fibrosis progression in MASLD. We further show that circulating sol-MerTK levels are higher in premenopausal than in postmenopausal women, and that estrogen increases circulating sol-MerTK levels in mice and promotes MerTK cleavage in macrophages. Given the reported roles of estrogen and MerTK cleavage in MASLD-related fibrosis, our study provides potential mechanistic insights into sex differences in MASLD-related fibrosis.

## Highlights

• Circulating sol-MerTK is negatively associated with fibrosis stages in men with MASLD but not in women.
• Circulating sol-Axl and Gas6 are positively associated with fibrosis stages in both sexes.
• Premenopausal women with MASLD have higher circulating sol-MerTK than postmenopausal women.
• Estrogen increases circulating sol-MerTK levels in mice and promotes MerTK cleavage in macrophages.

## Graphical abstract

**Figure:**
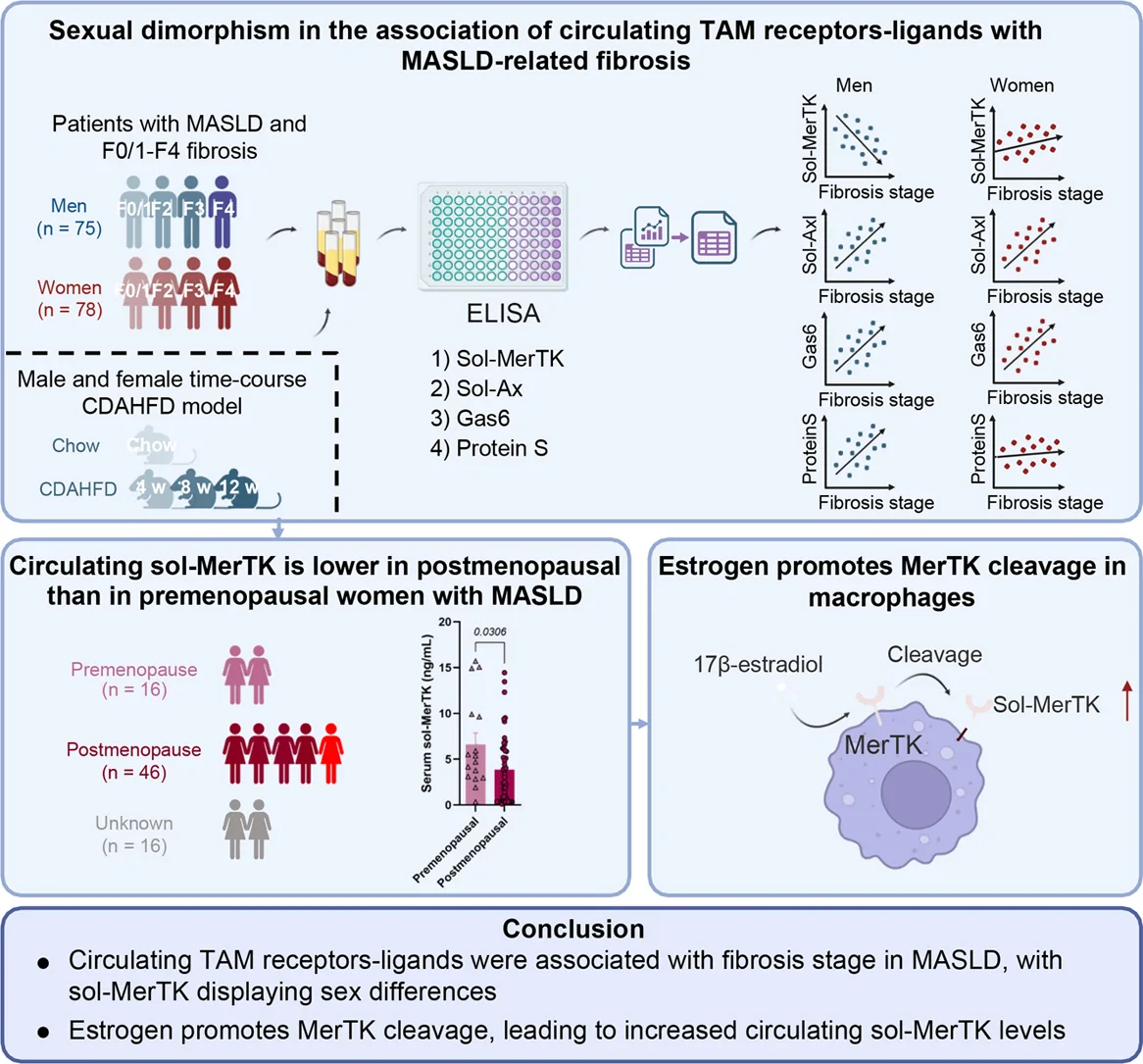

## Introduction

Metabolic dysfunction-associated steatotic liver disease (MASLD) is the most prevalent chronic liver condition worldwide, affecting approximately 25% of the general population, and is the leading indication for liver transplantation in women.^1^^,^^2^ MASLD encompasses a disease spectrum ranging from simple steatosis to metabolic dysfunction-associated steatohepatitis (MASH) and can progress to liver fibrosis. Liver fibrosis is the major determinant of adverse clinical outcomes including hepatocellular carcinoma, making it the central focus of both preclinical research and therapeutic development in MASLD.^3^ Staged from F0 (no fibrosis) to F4 (cirrhosis), liver fibrosis stage ≥ F2 (considered significant fibrosis) is associated with a higher risk of morbidity and mortality, and therefore clinical trials in MASLD have largely focused on patients with fibrosis stage ≥ F2.[4], [5], [6] Natural history studies show that fibrosis progression in MASLD is highly heterogeneous: Although many individuals progress slowly, taking approximately seven years to advance by one fibrosis stage, up to 20 percent may be classified as ‘‘rapid progressors” who develop cirrhosis within a decade.^7^ However, it is unclear why some patients progress rapidly whereas others remain stable for years. The divergence in clinical outcomes reflects a complex interplay among metabolic stress, inflammatory responsiveness, immune cell activation, and fibrogenesis that vary between individuals. Elucidating the mediators associated with fibrosis stages in MASLD is therefore essential for understanding the mechanisms driving fibrosis progression, identifying patients with at-risk MASLD, and guiding the development of more effective and individualized anti-fibrotic therapies in MASLD.

MASLD is a sex-dimorphic disease, with its development and progression influenced by sex chromosomes and sex hormones.^8^ In general, men show a higher prevalence of MASLD than women.^9^^,^^10^ Premenopausal women have a lower risk of developing MASLD and fibrosis progression compared with men; however, after menopause, the loss of estrogen-mediated protection is associated with a comparable or even higher prevalence of advanced hepatic fibrosis in women.^11^ Despite the well-recognized sexual dimorphism in MASLD development, progression, and outcomes, our understanding of the mechanisms underlying sex differences in MASLD remains limited. Moreover, sex remains insufficiently considered as a biological variable in the design of preclinical and clinical studies.^8^ Although a recent systematic review showed meaningful improvement in the representation of women in MASLD clinical trials,^12^ balanced enrollment alone does not ensure sex-aware study design or sex-stratified analysis.^8^^,^^13^ In addition, experimental studies and the development of new disease models have predominantly relied upon male animals.^14^^,^^15^ These limitations may have contributed to discrepancies in study outcomes and may hinder the translation of effective therapies, particularly for women. Therefore, incorporating sex as a biological variable in MASLD-related research is essential to enhance the translational relevance of preclinical findings and ensuring equitable diagnostic and therapeutic strategies for both sexes.

TAM (Tyro3, Axl, and MerTK) receptor tyrosine kinases are recognized as important regulators of fibrosis.[16], [17], [18], [19] By engaging their ligands Gas6 and Protein S, TAM receptors, particularly Axl and MerTK, activate downstream signaling pathways that exacerbate hepatic fibrogenesis in MASLD.^16^^,^^20^ The essential roles of TAM receptors in liver fibrosis are further supported by human genome-wide association studies (GWAS), which have linked two intronic single nucleotide polymorphisms (SNPs) in MERTK, rs4374383 and rs6726639, to liver fibrosis in patients with MASLD and HCV.[21], [22], [23] MerTK and Axl ectodomains undergo ADAM metallopeptidase-mediated cleavage, generating soluble MerTK (sol-MerTK) and soluble Axl (sol-Axl). Blocking MerTK cleavage in cleavage-resistant MerTK mutant mice has been shown to exacerbate fibrosis in MASLD.^16^ Moreover, circulating levels of TAM components have been linked to liver fibrosis in MASLD. However, the circulating patterns of TAM receptors and their ligands across liver fibrosis stages in MASLD remain poorly understood. Furthermore, it remains unknown whether their functions and circulating levels differ by sex in the setting of MASLD. Therefore, this study aimed to characterize circulating levels of TAM receptors and their ligands across fibrosis stages in MASLD and to define their associations with fibrosis severity, with analyses stratified by sex.

In this study, we characterized significant associations between circulating TAM receptors and their ligands and liver fibrosis severity, with circulating sol-MerTK exhibiting sex-specific associations with fibrosis severity in MASLD. Specifically, circulating sol-MerTK was negatively associated with fibrosis stage in men with MASLD and male MASLD mice, but showed no significant association in women with MASLD and female MASLD mice. We further made a novel observation that circulating sol-MerTK levels were higher in premenopausal than postmenopausal women with MASLD. Consistently, 17β-estradiol-treated mice had higher circulating sol-MerTK levels than controls. Finally, we demonstrated that estrogen induced MerTK cleavage, leading to increased sol-MerTK released from macrophages. Overall, our findings highlight the estrogen–MerTK axis as a plausible determinant of underlying sex differences in MASLD-related fibrosis.

## Material and methods

### Study cohort

The study included two independent study cohorts. The main cohort included 153 patients enrolled in the Mount Sinai MASLD/MASH Registry and Biobank.[24], [25] The CLRC cohort included 36 patients from the UCLA Comprehensive Liver Research Center Human MASLD Core. The Mount Sinai MASLD/MASH Registry and Biobank is an ongoing, longitudinal prospective cohort study. Eligible participants were identified from the Mount Sinai Hepatology Practice based on a clinical diagnosis of MASLD. Patients were approached for consent during routine clinical visits. Exclusion criteria included active hepatitis C virus (HCV) infection, untreated hepatitis B virus (HBV) infection, excessive alcohol use, or other chronic liver disease etiologies, ensuring a homogeneous cohort focused on MASLD. Upon enrollment, comprehensive baseline data were collected, including demographics, lifestyle factors, comorbidities, detailed metabolic profiling, and a broad panel of laboratory parameters. Exposure to alcohol was quantitatively assessed using the Alcohol Use Disorders Identification Test (AUDIT-C) along with a detailed review of the electronic health record (EHR). Liver stiffness was assessed by vibration-controlled transient elastography (VCTE), and fibrosis staging was defined as F0 to F1 (<8 kPa), F2 (8 to <10 kPa), F3 (10 to <14 kPa), and F4 (≥14 kPa). Among the 153 patients included from the Mount Sinai Biobank, 14 underwent liver biopsy within 1 year of the baseline date, 30 had biopsies performed more than one year apart from the baseline, and 109 had no biopsy data available. All the studies with human serum samples were approved by Institutional Review Boards (IRB) at the Icahn School of Medicine at Mount Sinai and UCLA.

### Animal model

To induce MASLD, 6–8-week-old male and female wild-type C57BL/6J mice (Jackson Laboratory, #000664) were randomly assigned to receive either a chow diet or a choline-deficient, L-amino acid–defined, high-fat diet (CDAHFD; Research Diets, #A06071302) for 4, 8, or 12 weeks (*n* = 5 per group). The male and female cohorts were established independently, with the male cohort was generated at the Icahn School of Medicine at Mount Sinai and the female cohort was generated at UCLA. For *in vivo* estrogen supplementation, male mice underwent subcutaneous implantation of 17β-estradiol pellets (0.001 mg/pellet; Innovative Research of America, #E-121) for 21 days. Age-matched male mice were used as controls. Food and water were provided *ad libitum*. Mice were housed in a specific pathogen-free facility under a 12-hour light/dark cycle. All animal procedures were approved by the Institutional Animal Care and Use Committee of the Icahn School of Medicine at Mount Sinai or by the UCLA Chancellor’s Animal Research Committee.

### Statistical analysis

Spearman’s correlation analysis was used to analyze correlations between two parameters. Statistical significance was evaluated using the Kruskal-Wallis test with Dunn’s post hoc correction (Bonferroni corrected), one-way ANOVA followed by Tukey’s post hoc test, the two-tailed Mann-Whitney test, or the two-tailed unpaired t-test, as appropriate. Continuous variables for patients’ baseline characteristics are presented as the mean ± SD. All other results are expressed as the mean ± SEM, and statistical significance was defined as *p* < 0.05. Statistical analyses were performed using GraphPad Prism and R software.

Full details of the materials and methods are provided in the Supplementary Materials.

## Results

### Circulating TAM receptors and their ligands are associated with liver fibrosis severity in men with MASLD

**Fig. 1:**
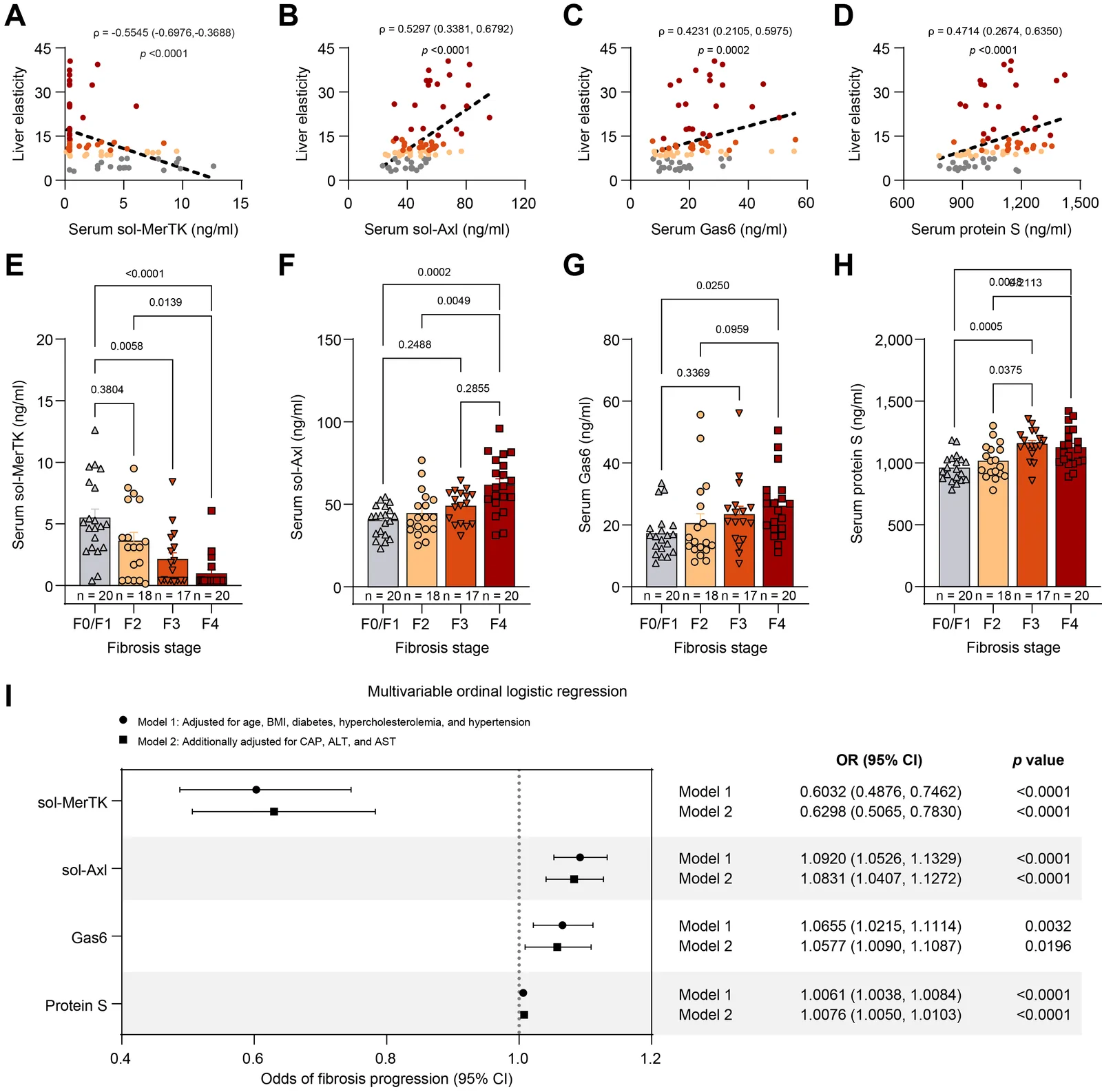
Serum sol-MerTK, sol-Axl, Gas6, and Protein S levels are associated with liver fibrosis severity in men with MASLD. (A–D) Spearman’s correlation analyses showing the correlations between liver elasticity (kPa) and serum sol-MerTK (A), sol-Axl (B), Gas6 (C), and Protein S (D) in men with MASLD (*n* = 75). (E–H) Serum levels of sol-MerTK (E), sol-Axl (F), Gas6 (G), and Protein S (H) in men with MASLD and different liver fibrosis stages (F0/1–F4; *n* = 17–20 per group). (I) Multivariable ordinal regression analysis showing the associations between liver fibrosis stage and serum levels of sol-MerTK, sol-Axl, Gas6, and Protein S. Model 1 was adjusted for age, BMI, diabetes, hypercholesterolemia, and hypertension; Model 2 was additionally adjusted for CAP, ALT, and AST. Data are shown as scatter plots with bars representing the mean ± SEM; Kruskal-Wallis with Dunn’s post hoc test (Bonferroni corrected).

Serum levels of sol-MerTK, sol-Axl, Gas6, and Protein S were measured in men with MASLD and different degrees of liver fibrosis (*n* = 75, 17–20 per fibrosis stage; from Mount Sinai cohort) to investigate their associations with fibrosis severity. Baseline characteristics of patients were shown in Table 1. Among male patients in this cohort, CAP score, serum AST levels, and the rate of hypertension differed significantly across fibrosis stages (Table 1). Spearman’s correlation analysis showed that serum sol-MerTK levels were negatively correlated with liver stiffness (Fig. 1A), whereas serum sol-Axl, Gas6, and Protein S levels were positively correlated with liver stiffness in men with MASLD (Fig. 1B–D). We further compared serum levels of sol-MerTK, sol-Axl, Gas6, and Protein S across fibrosis stages (F0/F1 to F4) in men with MASLD. Serum sol-MerTK levels gradually decreased with increasing fibrosis stage (Fig. 1E), consistent with our previous finding that MerTK cleavage is suppressed in fibrotic livers compared with steatotic livers in male MASLD mice.^16^ In contrast, serum sol-Axl, Gas6, and Protein S levels showed an increasing trend from F0/F1 to F4 and were significantly higher in the F4 stage than in earlier stages (F0/1 and F2; Fig. 1F–H). Moreover, multivariable ordinal regression analysis adjusted for age, BMI, and metabolic comorbidities showed a significant inverse association between serum sol-MerTK levels and fibrosis stage (OR = 0.6032, 95% CI = 0.4876–0.7462, *p* < 0.0001), indicating that each unit increase in sol-MerTK was associated with a 39.68% reduction in the odds of being in a higher liver fibrosis stage (Fig. 1I, Model 1). Meanwhile, sol-Axl, Gas6, and Protein S levels were positively associated with liver fibrosis stages (Fig. 1I, Model 1). Further adjustment for ALT, AST, and CAP score did not change the observed associations (Fig. 1I, Model 2). In addition, sensitivity analysis excluding patients with extremely high BMI and ALT further supported the robustness of our observations (Supplementary Fig. S1A).

**Table 1:** Baseline characteristics by sex and fibrosis stage in main cohort.

	Female (n = 78)					Male (n = 75)				
	F0–F1 (n = 20)	F2 (n = 18)	F3 (n = 20)	F4 (n = 20)	*p* values	F0–F1 (n = 20)	F2 (n = 18)	F3 (n = 17)	F4 (n = 20)	*p* values
**Continuous variables, mean (SD)**										
Liver stiffness, kPa	4.6 (1.0)	8.8 (0.5)	11.7 (1.1)	26.5 (13.1)	<0.0001^a^	5.0 (1.4)	9.1 (0.6)	11.8 (1.1)	26.9 (8.7)	<0.0001^a^
CAP, dB/m	288.8 (52.9)	328.8 (62.7)	326.8 (68.1)	309.9 (69.0)	0.0396^a^	298.7 (49.5)	305.2 (59.4)	275.5 (79.2)	343.7 (39.6)	0.0162^a^
Age, years	58.7 (16.1)	54.4 (13.5)	62.8 (10.7)	59.6 (12.9)	0.3016^a^	48.0 (19.9)	49.5 (16.7)	53.4 (13.9)	55.1 (11.2)	0.9268^a^
BMI, kg/m^2^	30.1 (4.8)	33.3 (6.1)	35.1 (9.2)	31.5 (10.2)	0.2235^a^	30.4 (6.2)	34.9 (8.4)	32.8 (4.8)	33.9 (5.7)	0.1805^a^
ALT, U/L	33.2 (17.6)	48.8 (20.6)	28.7 (17.5)	33.9 (12.3)	0.0097^a^	52.7 (47.1)	41.6 (19.7)	51.1 (22.4)	50.9 (23.1)	0.4990^a^
AST, U/L	27.2 (8.6)	36.4 (11.4)	27.0 (9.5)	36.4 (14.6)	0.0085^a^	31.6 (18.8)	32.0 (10.2)	44.4 (21.3)	46.8 (21.7)	0.0035^a^
Systolic BP, mmHg	131.4 (16.6)	127.0 (15.9)	124.9 (14.0)	132.3 (18.8)	0.4351^b^	126.9 (13.0)	121.6 (13.5)	121.7 (13.6)	120.1 (13.2)	0.4082^b^
Diastolic BP, mmHg	75.8 (12.4)	76.6 (7.6)	75.6 (11.4)	77.6 (11.3)	0.9359^b^	79.9 (5.6)	77.6 (9.2)	77.1 (7.1)	74.8 (7.9)	0.2256^b^

**Comorbidities, n (%)**										
Obesity					0.1390^c^					0.7035^c^
Yes	8 (40.0%)	13 (72.2%)	13 (65.0%)	14 (70.0%)		12 (60.0%)	12 (66.7%)	10 (58.8%)	15 (75.0%)	
No	12 (60.0%)	5 (27.8%)	7 (35.0%)	6 (30.0%)		8 (40.0%)	6 (33.3%)	7 (41.2%)	5 (25.0%)	
Diabetes					0.9030^c^					0.5822^c^
Yes	10 (50.0%)	10 (55.6%)	9 (45.0%)	11 (55.0%)		6 (30.0%)	7 (38.9%)	8 (47.1%)	10 (50.0%)	
No	10 (50.0%)	8 (44.4%)	11 (55.0%)	9 (45.0%)		14 (70.0%)	11 (61.1%)	9 (52.9%)	10 (50.0%)	
Hypertension					0.6759^c^					0.0112^c^
Yes	10 (50.0%)	12 (66.7%)	13 (65.0%)	13 (65.0%)		6 (30.0%)	10 (55.6%)	13 (76.5%)	6 (30.0%)	
No	10 (50.0%)	6 (33.3%)	7 (35.0%)	7 (35.0%)		14 (70.0%)	8 (44.4%)	4 (23.5%)	14 (70.0%)	
Hypercholesterolemia					0.2366^c^					0.6259^c^
Yes	8 (40.0%)	11 (61.1%)	12 (60.0%)	7 (35.0%)		5 (25.0%)	8 (44.4%)	6 (35.3%)	8 (40.0%)	
No	12 (60.0%)	7 (38.9%)	8 (40.0%)	13 (65.0%)		15 (75.0%)	10 (55.6%)	11 (64.7%)	12 (60.0%)	
Kidney disease					0.7446^d^					0.7136^d^
Yes	2 (10.0%)	0 (0.0%)	2 (10.0%)	1 (5.0%)		2 (10.0%)	0 (0.0%)	1 (5.9%)	2 (10.0%)	
No	18 (90.0%)	18 (100.0%)	18 (90.0%)	19 (95.0%)		18 (90.0%)	18 (100.0%)	16 (94.1%)	18 (90.0%)	

**Menopausal status, n (%)**					0.8021^d^					
Pre-menopausal	4 (20.0%)	5 (27.8%)	2 (10.0%)	5 (25.0%)						
Post-menopausal	12 (40.0%)	12 (66.7%)	11 (55.0%)	11 (55.0%)						

**Hormone replacement therapy, n (%)**										
Yes	0 (0.0%)	0 (0.0%)	2 (10.0%)	2 (10.0%)						
No	20 (100.0%)	18 (100.0%)	18 (90.0%)	18 (90.0%)						

**Ethnicity, n (%)**					0.2814^d^					0.9615^c^
Hispanic										
Yes	8 (40.0%)	2 (11.1%)	7 (35.0%)	5 (25.0%)		9 (45.0%)	7 (38.9%)	6 (35.3%)	7 (35.0%)	
No	9 (45.0%)	11 (61.1%)	11 (55.0%)	13 (65.0%)		8 (40.0%)	7 (38.9%)	6 (35.3%)	9 (45.0%)	

**Race, n (%)**					0.4858^d^					0.7626^d^
Asian	2 (10.0%)	0 (0.0%)	1 (5.0%)	1 (5.0%)		2 (10.0%)	1 (5.6%)	1 (5.9%)	1 (5.0%)	
Black	2 (10.0%)	1 (5.6%)	3 (15.0%)	0 (0.0%)		3 (15.0%)	4 (22.2%)	1 (5.9%)	0 (0.0%)	
Native	1 (5.0%)	0 (0.0%)	0 (0.0%)	1 (5.0%)		0 (0.0%)	0 (0.0%)	1 (5.9%)	1 (5.0%)	
Other	8 (40.0%)	14 (77.8%)	11 (55.0%)	13 (65.0%)		8 (40.0%)	6 (33.3%)	6 (35.3%)	9 (45.0%)	
White	7 (35.0%)	3 (16.7%)	5 (25.0%)	4 (20.0%)		6 (30.0%)	7 (38.9%)	7 (41.2%)	7 (35.0%)	

Note 1: Missing values for continuous variables were as follows: liver stiffness, age, and BMI had 0% missing; CAP 6 (3.9%), ALT 4 (2.6%), AST 4 (2.6%), systolic blood pressure 1 (0.7%), and diastolic blood pressure 1 (0.7%). Missing values for categorical variables were as follows: obesity, diabetes, hypertension, hypercholesterolemia, kidney disease, and hormone replacement therapy had 0% missing; menopausal status 16 (20.5%), ethnicity 28 (18.3%), and race 5 (3.3%).

Note 2: Statistical significance was determined using:

a Kruskal-Wallis test.

b one-way ANOVA.

c Chi-square test.

d Fisher’s Exact Test. All p values were calculated based on available data; missing values were not imputed.

We further validated our findings in the male subset of an independent MASLD cohort (CLRC cohort, Supplementary Table S2). Due to the limited sample size, patients were stratified into early (F0–F1; *n* = 9) and significant (F2–F4; *n* = 11) fibrosis groups. Notably, serum sol-MerTK levels exhibited a marginally significant reduction (*p* = 0.0783) in the F2–F4 stages relative to the F0–F1 stages in men with MASLD (Supplementary Fig. S2A). Serum sol-Axl (*p* = 0.2295) and Gas6 (*p* = 0.2384) levels were not significantly elevated (Supplementary Fig. S2B and C), possibly due to the small number of patients with F4 fibrosis (*n* = 2). These results are consistent with the observations in our main cohort. Overall, our data suggest that lower circulating sol-MerTK and higher sol-Axl, Gas6, and Protein S levels are related to more advanced liver fibrosis in men with MASLD.

### Circulating TAM receptors and their ligands are associated with liver fibrosis severity in women with MASLD

**Fig. 2:**
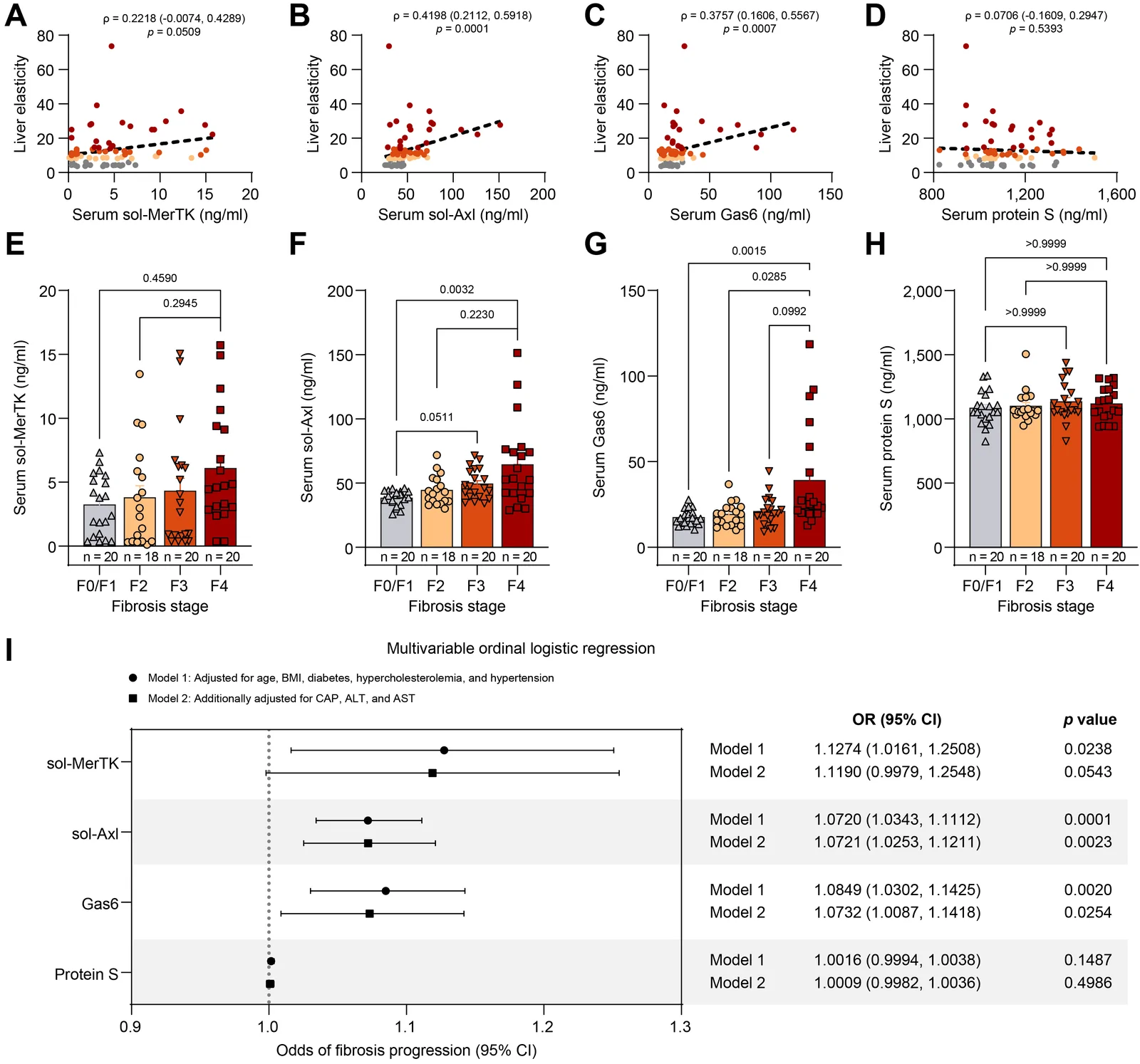
Serum sol-MerTK and Protein S are not, but sol-Axl and Gas6 are, positively associated with liver fibrosis severity in women with MASLD. (A–D) Spearman’s correlation analyses showing the correlations between liver elasticity (kPa) and serum sol-MerTK (A), sol-Axl (B), Gas6 (C), and Protein S (D) in women with MASLD (*n* = 78). (E–H) Serum levels of sol-MerTK (E), sol-Axl (F), Gas6 (G), and Protein S (H) in women with MASLD and different fibrosis stages (F0/1–F4; *n* = 18–20 per group). (I) Multivariable ordinal regression analysis showing the associations between liver fibrosis stage and serum levels of sol-MerTK, sol-Axl, Gas6, and Protein S. Model 1 was adjusted for age, BMI, diabetes, hypercholesterolemia, and hypertension; Model 2 was additionally adjusted for CAP, ALT, and AST. Data are shown as scatter plots with bars representing the mean ± SEM; Kruskal-Wallis with Dunn’s post hoc test (Bonferroni corrected).

We also evaluated serum levels of sol-MerTK, sol-Axl, Gas6, and Protein S in women with MASLD from the Mount Sinai cohort (*n* = 78, 18–20 per fibrosis stage). Interestingly, in contrast to our findings in men, the serum levels of sol-MerTK showed a borderline positive correlation with liver stiffness in women with MASLD (*p* = 0.0509, Fig. 2A). Meanwhile, despite an increasing trend from F0/1 to F4, serum sol-MerTK levels did not differ significantly among liver fibrosis stages in women with MASLD (Fig. 2E). In multivariable ordinal regression analysis adjusted for age, BMI, and metabolic comorbidities, we observed a weak positive association between serum sol-MerTK levels and fibrosis stage (OR = 1.1274, 95% CI = 1.0161–1.2508, *p* = 0.0238; Fig. 2I, Model 1). However, this association was no longer significant following further adjustment for ALT, AST, and CAP (OR = 1.1190, 95% CI = 0.9979–1.2548, *p* = 0.0543; Fig. 2I, Model 2). We also found a sex difference regarding serum Protein S levels, which showed no association with liver fibrosis severity in women with MASLD (Fig. 2D, H, I). Conversely, serum sol-Axl and Gas6 levels were positively associated with fibrosis severity in women with MASLD (Fig. 2B, C, I), consistent with the findings observed in men with MASLD. Notably, serum sol-Axl and Gas6 levels were significantly higher in the F4 fibrosis stage than in early stages (F0/1–F2; Fig. 2F, G). Sensitivity analysis excluding patients with extremely high BMI and ALT did not alter these observations (Supplementary Fig. S1B). When examined in the female subset from the CLRC cohort, serum sol-MerTK (*p* = 0.3527), sol-Axl (*p* = 0.2432), and Gas6 (*p* = 0.1145) levels did not differ significantly between the F0–F1 (*n* = 7) and F2–F4 (*n* = 9) groups (Supplementary Fig. S2D–F). The absence of significant differences in sol-Axl and Gas6 may be due to the limited sample size, particularly patients with F4 fibrosis (*n* = 2). Taken together, these observations demonstrate sex-specific associations of circulating sol-MerTK and Protein S with liver fibrosis severity in patients with MASLD, while the associations of circulating sol-Axl and Gas6 were consistent between sexes.

### Circulating TAM receptors and their ligands are associated with liver fibrosis severity in male and female MASLD mice

**Fig. 3:**
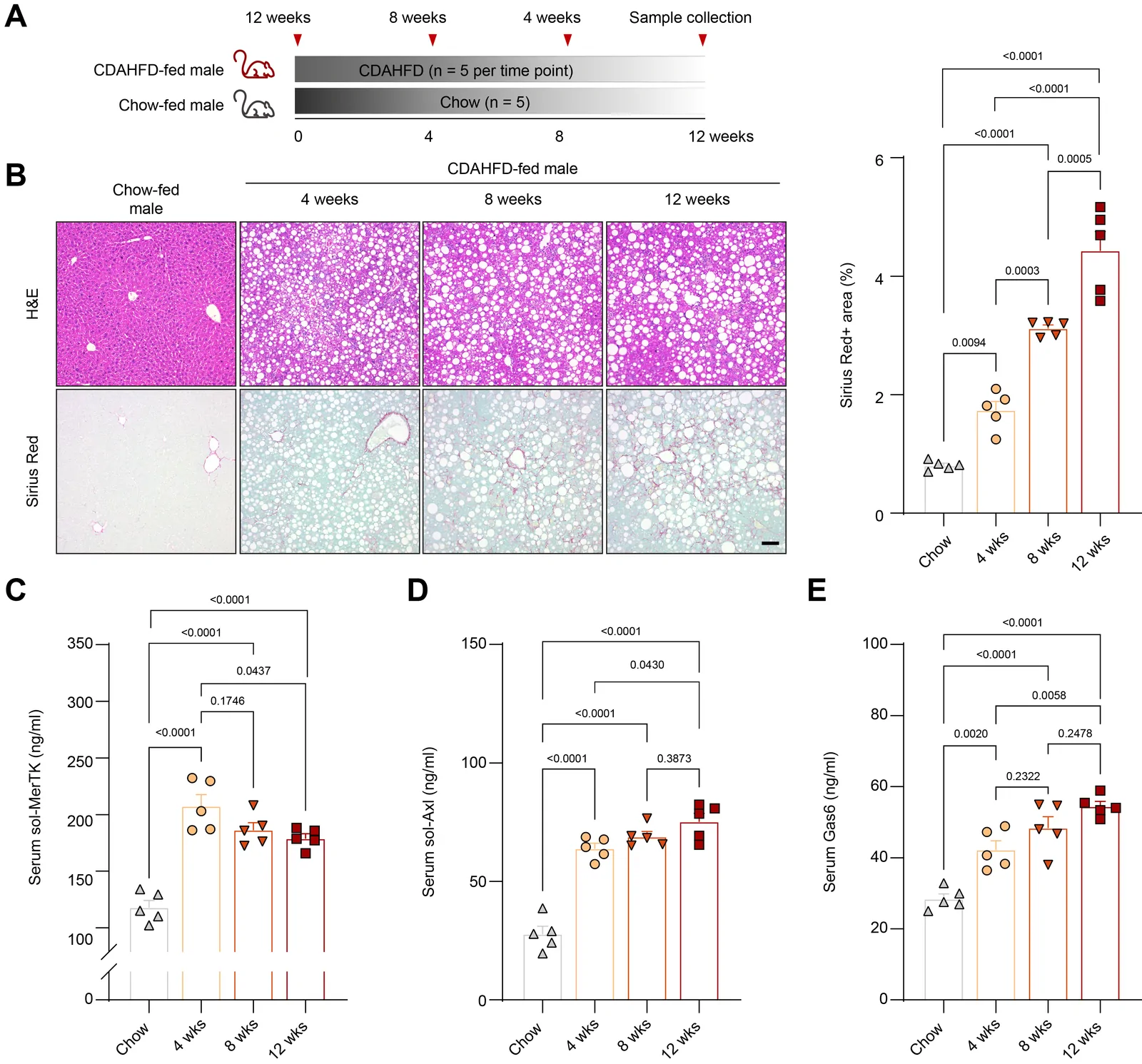
Serum sol-MerTK, sol-Axl, and Gas6 levels are associated with MASLD development and fibrosis progression in CDAHFD-fed male mice. Male mice were fed a chow or CDAHFD diet for 4, 8, or 12 weeks (*n* = 5 per group). (A) Schematic diagram. (B) Representative H&E and Sirius red staining images (left) and quantification of percent Sirius red^+^ area (right). Scale bar, 100 μm. (C–E) Serum levels of sol-MerTK (C), sol-Axl (D), and Gas6 (E). Data are shown as scatter plots with bars representing the mean ± SEM; one-way ANOVA followed by Tukey’s post hoc test.

**Fig. 4:**
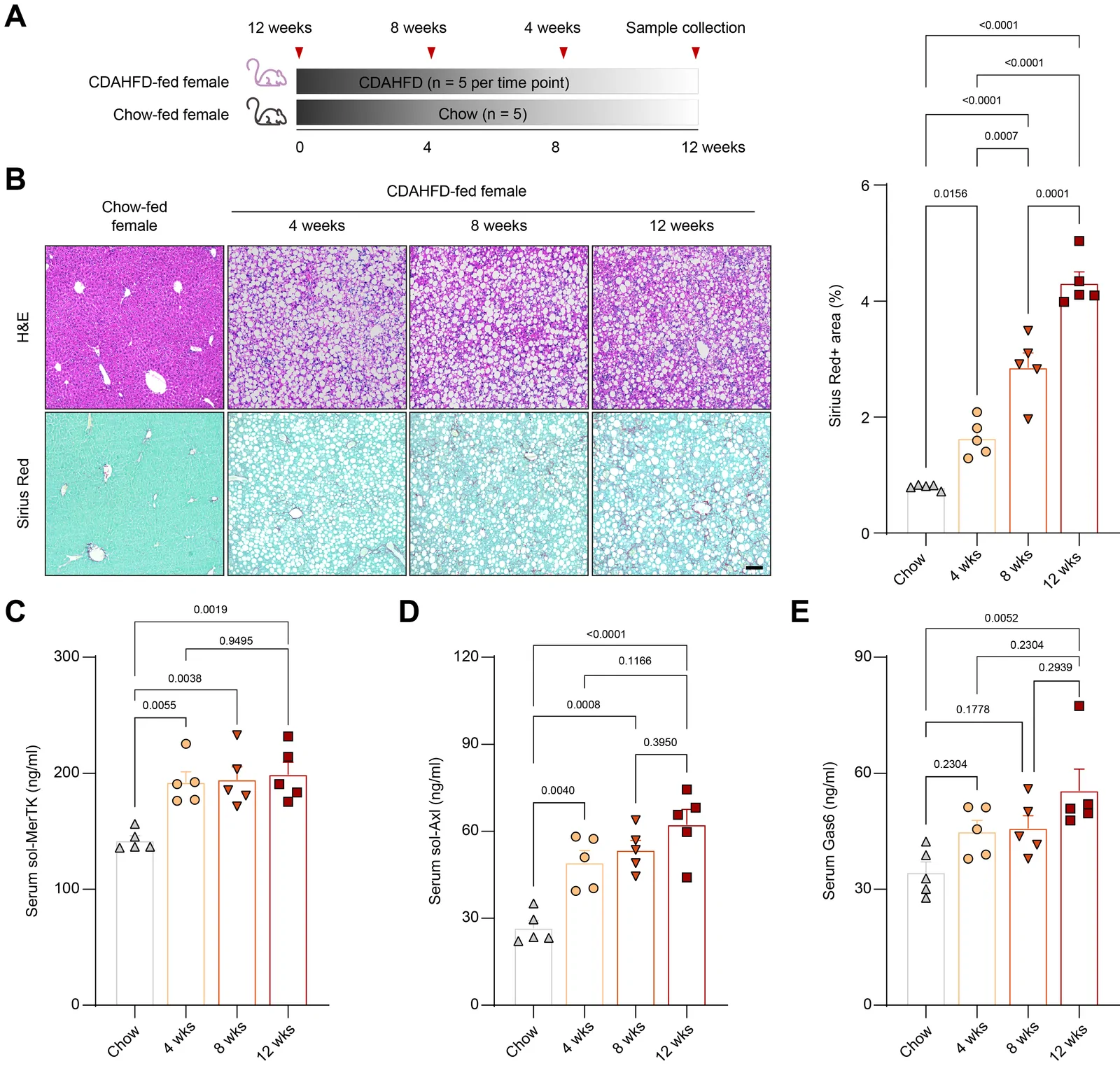
The associations of serum sol-MerTK, sol-Axl, and Gas6 levels with liver fibrosis are characterized in CDAHFD-fed female mice. Female mice were fed a chow or CDAHFD diet for 4, 8, or 12 weeks (*n* = 5 per group). (A) Schematic diagram. (B) Representative H&E and Sirius red staining images (left) and quantification of percent Sirius red^+^ area (right). Scale bar, 100 μm. (C-E) Serum levels of sol-MerTK (C), sol-Axl (D), and Gas6 (E). Data are shown as scatter plots with bars representing the mean ± SEM; one-way ANOVA followed by Tukey’s post hoc test.

To validate the clinical observations, we separately generated time-course CDAHFD models in male mice and female mice to recapitulate MASLD development and fibrosis progression (Fig. 3, Fig. 4A). Histological analyses demonstrated that CDAHFD-fed mice developed hepatic steatosis, inflammation, and fibrosis at all examined time points (4, 8, and 12 weeks) compared with chow-fed controls in both male (Fig. 3B and Supplementary Table S3) and female (Fig. 4B and Supplementary Table S4) mice model. Of note, hepatic steatohepatitis severity was similar across different time points, whereas fibrosis severity progressively increased with the duration of CDAHFD feeding (Fig. 3, Fig. 4B, Supplementary Tables S3 and S4). Therefore, this CDAHFD model may be relevant for investigating fibrosis progression in MASLD.

Next, serum levels of sol-MerTK, sol-Axl, and Gas6 were measured. In the male model, CDAHFD feeding significantly increased serum sol-MerTK, sol-Axl, and Gas6 levels at all time points compared with chow-fed controls (Fig. 3C–E). Within the CDAHFD time-course groups (4, 8, and 12 weeks), serum sol-MerTK levels progressively decreased, while serum sol-Axl and Gas6 levels increased during the feeding period (Fig. 3C–E). Serum sol-MerTK levels were significantly lower, and sol-Axl and Gas6 levels were significantly higher in mice with intense perisinusoidal liver fibrosis (12 weeks) than in those with mild liver fibrosis (4 weeks; Fig. 3C–E). These results are consistent with the findings in men with MASLD. We also determined mRNA expression levels of hepatic *Mertk*, *Axl*, and *Gas6* in the male CDAHFD model. The results showed that the mRNA expression levels of hepatic *Mertk*, *Axl*, and *Gas6* were significantly higher in 4, 8, and 12-week CDAHFD-fed mice than in chow-fed controls (Supplemental Fig. S3A–C). Notably, hepatic *Mertk* and *Axl* mRNA levels did not differ between 4, 8, and 12 weeks of CDAHFD feeding, whereas hepatic *Gas6* mRNA levels increased over time (Supplemental Fig. S3A–C). These data suggest that CDAHFD-induced hepatic steatohepatitis may be associated with increased transcription of hepatic *Mertk*, *Axl*, and *Gas6* in males. Furthermore, liver fibrosis progression appears to be associated with decreased cleavage of MerTK but enhanced cleavage of Axl in male MASLD mice.

In the female CDAHFD model, serum levels and hepatic mRNA expression of Axl and Gas6 showed trends similar to those observed in males (Fig. 4D, E, and Supplemental Fig. S4B and C). Conversely, MerTK exhibited a sex-specific pattern. Unlike in males, serum sol-MerTK levels showed no significant differences among the 4, 8, and 12-week CDAHFD groups in females (Fig. 4C). Furthermore, hepatic *Mertk* mRNA levels did not differ significantly between chow-fed and CDAHFD-fed female mice, despite an upward trend (Supplemental Fig. S4A). Overall, these murine findings further support our clinical observations that the association between circulating sol-MerTK and fibrosis stages is sex-specific, whereas the associations for circulating sol-Axl and Gas6 remain consistent between sexes.

### Serum sol-MerTK levels are lower in postmenopausal than in premenopausal women with MASLD

**Fig. 5:**
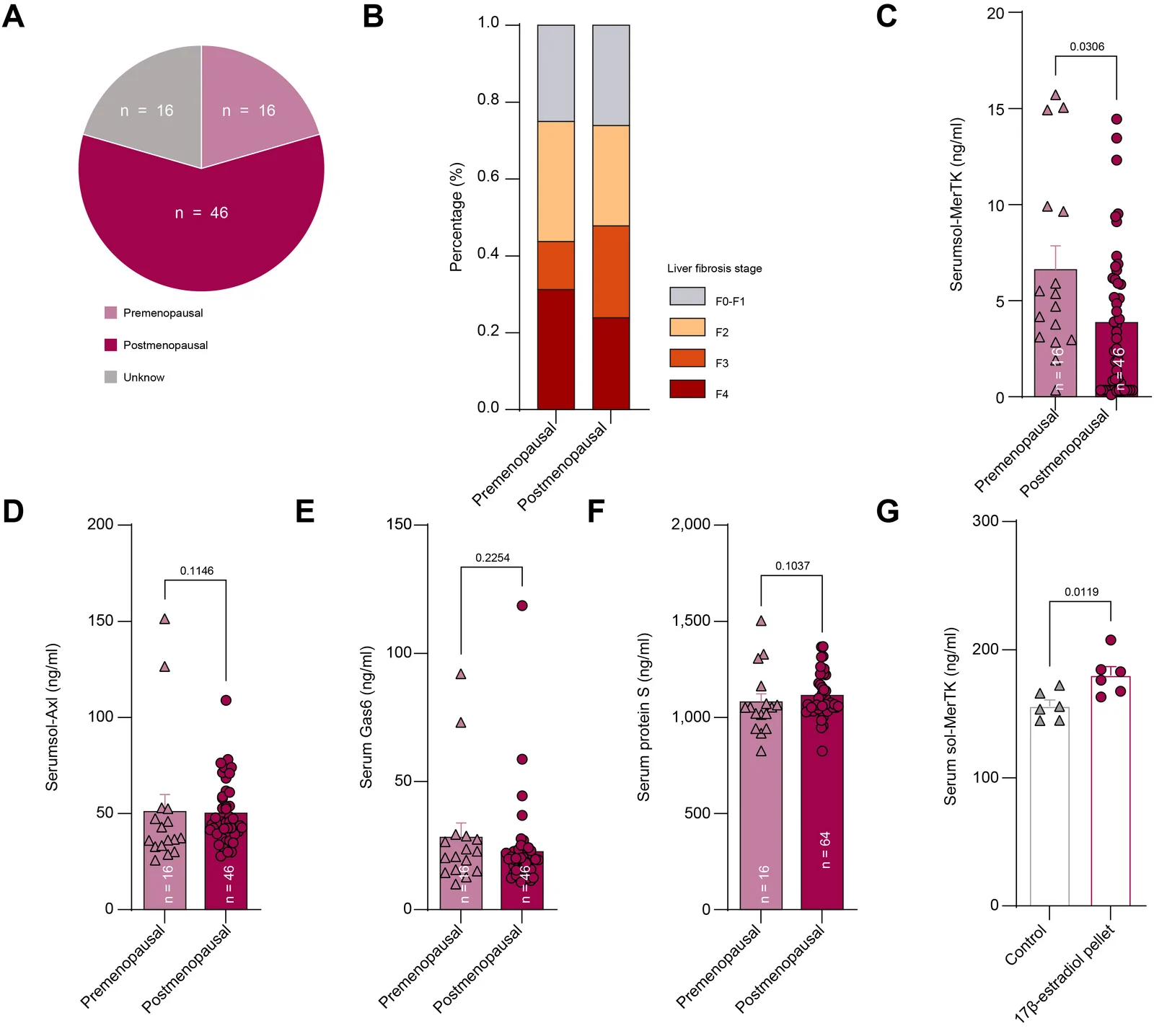
Serum sol-MerTK levels are lower in postmenopausal than in premenopausal women with MASLD. (A) Pie chart showing menopausal status distribution in 78 women with MASLD, based on self-reported information. (B) Stacked bar plot showing the percentage composition of liver fibrosis stages (F0/1–F4) in premenopausal and postmenopausal women. (C–F) Serum levels of sol-MerTK (C), sol-Axl (D), Gas6 (E), and Protein S (F) in premenopausal (*n* = 16) and postmenopausal (*n* = 46) women with MASLD; two-tailed Mann-Whitney test. (G) Serum sol-MerTK levels in male mice implanted with subcutaneous 17β-estradiol pellets for 21 days (*n* = 6) or in control mice (*n* = 6); two-tailed unpaired t-test. Data are shown as scatter plots with bars representing the mean ± SEM.

To determine whether menopausal status influenced serum levels of sol-MerTK, sol-Axl, Gas6, and Protein S, we compared their serum levels in premenopausal and postmenopausal women with MASLD. Among the 78 women in our main cohort, 16 were premenopausal, 46 were postmenopausal, and 16 with unknown menopausal status, as determined by self-report (Fig. 5A and Table 1). The percentage composition of premenopausal and postmenopausal women with different liver fibrosis stages in our main MASLD cohort was shown in Figure 5B. Remarkably, we found that serum sol-MerTK levels were significantly lower in postmenopausal than in premenopausal group (Fig. 5C). However, no significant differences were observed in serum sol-Axl, Gas6, and Protein S levels between the postmenopausal and premenopausal groups (Fig. 5D–F). Given that four women had received hormone replacement therapy (HRT; Table 1), we conducted a sensitivity analysis excluding these cases. As shown in Supplemental Fig. S5, our sensitivity analysis results were consistent with the original findings. In fact, exclusion of these patients resulted in a more significant reduction in serum sol-MerTK levels (Supplemental Fig. S5A). These results suggest that menopause-related declines in estrogen may contribute to reduced circulating sol-MerTK.

To further validate whether estrogen affects circulating sol-MerTK, we measured serum sol-MerTK levels in male mice subcutaneously implanted with 17β-estradiol pellets, the prevalent endogenous estrogen in women.^26^ The results showed that serum sol-MerTK levels were significantly higher in 17β-estradiol-administered mice than in control mice (Fig. 5G). These results support our proposed role of estrogen in increasing circulating sol-MerTK.

Considering these findings, we excluded female patients with prior HRT and stratified the remaining patients by menopausal status to assess whether menopausal heterogeneity influences the relationship between circulating sol-MerTK and fibrosis severity in women with MASLD. As shown in Supplemental Fig. S6, no significant correlation between serum sol-MerTK and fibrosis severity was observed in either premenopausal (*n* = 16) or postmenopausal (*n* = 42) women with MASLD.

### 17β-estradiol induces MerTK cleavage in BMDMs

**Fig. 6:**
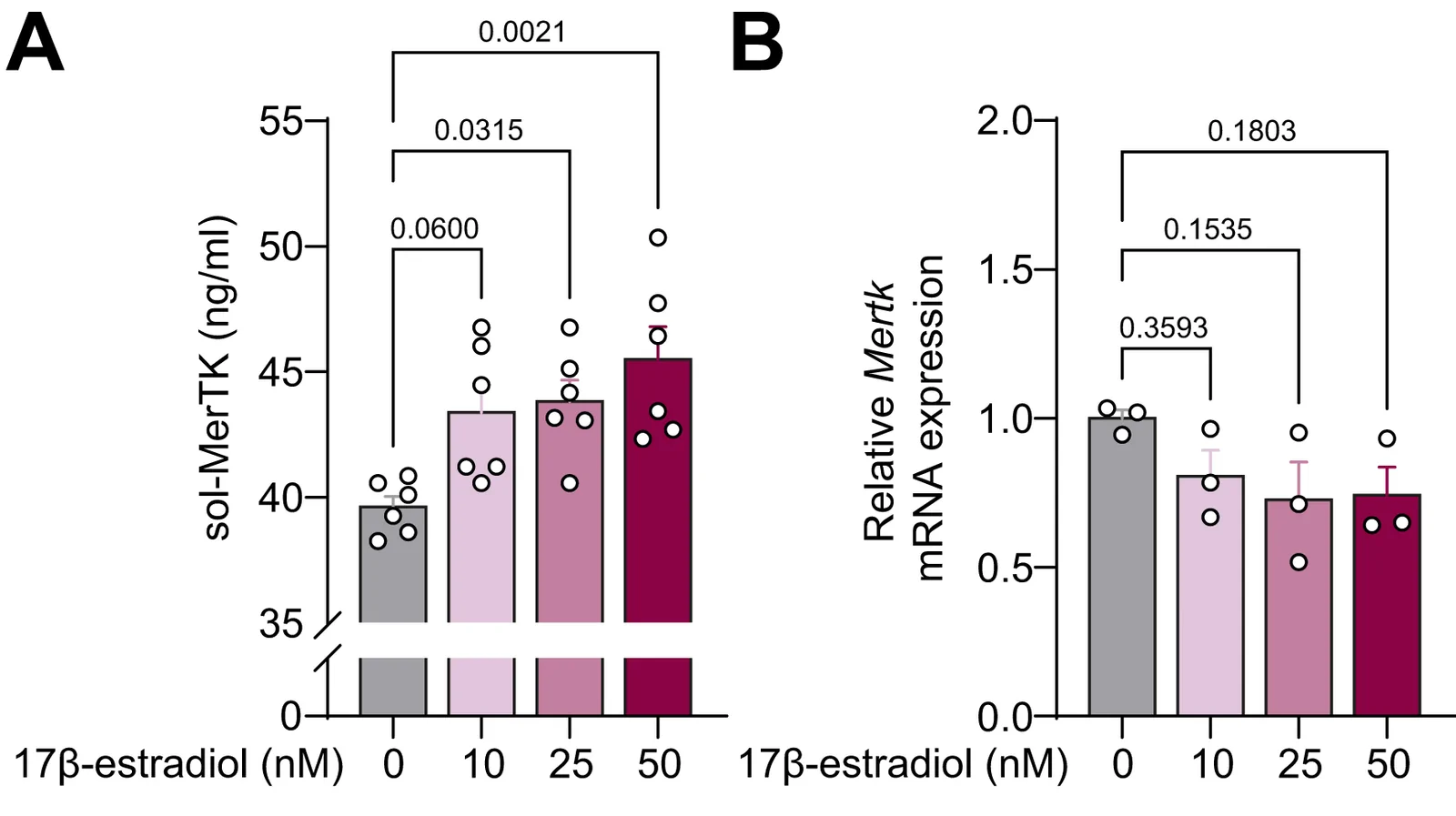
17β-estradiol induces MerTK cleavage in BMDMs. BMDMs were isolated from female mice and treated with different concentrations of 17β-estradiol for 24 h. (A) sol-MerTK levels in BMDM culture supernatants. (B) mRNA levels of *Mertk* in BMDMs. Data are shown as scatter plots with bars representing the mean ± SEM; one-way ANOVA followed by Tukey’s post hoc test.

As sol-MerTK is primarily produced by the cleavage of the ectodomain of MerTK in macrophages,^27^ we next sought to determine whether estrogen regulates MerTK cleavage in macrophages. We treated BMDMs with 17β-estradiol and found that 17β-estradiol treatment increased sol-MerTK levels in BMDM culture supernatants in a dose-dependent manner (Fig. 6A). Notably, 17β-estradiol treatment showed a non-significant trend toward reduced *Mertk* mRNA expression at the tested concentrations (Fig. 6B), suggesting that 17β-estradiol induces MerTK cleavage. These *in vitro* results are in line with our clinical observations that postmenopausal women with lower estrogen levels have reduced circulating sol-MerTK, and that 17β-estradiol administration increases circulating sol-MerTK in mice. Given that reduced MerTK cleavage has been shown to promote MASLD-related fibrosis in mice,^16^ our data suggest that estrogen-induced MerTK cleavage may play a role in MASLD-related fibrosis.

## Discussion

MASLD is a sex-dimorphic disease, with generally higher prevalence in men.^8^^,^^11^^,^^19^ Emerging evidence suggests that sexual dimorphism in MASLD is underpinned by sex-specific modulations of insulin sensitivity, hepatic lipid homeostasis, immune responses, and fibrogenesis.^8^^,^^28^^,^^29^ The TAM receptors MerTK and Axl together with their ligands Gas6 and Protein S play important roles in immune responses and fibrogenesis,^27^^,^^30^^,^^31^ and have been implicated in the development of fibrosis in MASLD.^16^^,^^17^ In this study, we for the first time analyzed circulating TAM receptors and their ligand levels separately in men and women with MASLD, with comparable numbers of patients of each sex at every liver fibrosis stage. Our findings revealed a striking sex-specific association, in that lower circulating sol-MerTK levels were associated with more advanced liver fibrosis in men with MASLD, whereas in women, sol-MerTK levels tended to increase with fibrosis progression but did not reach statistical significance. These observations were further supported by an independent validation cohort, although this cohort was limited by its small sample size and heterogeneous methods for fibrosis assessment. Given the established role of MerTK signaling in MASLD-related fibrogenesis,^16^ our observations suggest that MerTK-related pathways may be involved in sex-specific patterns of fibrogenesis during MASLD progression. It should be noted that fibrosis staging in the Mount Sinai cohort was based on FibroScan-derived liver stiffness, which may be influenced by hepatic steatosis and necroinflammation in addition to fibrosis.^32^ Nevertheless, additional adjustment for CAP score, ALT, and AST helped address this potential limitation. In contrast with our finding, previous studies have reported higher serum MerTK levels in patients with advanced versus early-stage liver fibrosis in their study cohorts.^18^^,^^19^ This discrepancy might be partly explained by two potential confounding factors: sex composition and genetic background. First, previous studies did not analyze their data separately by sex or adjust for sex as a potential confounding factor. Given the opposite trends observed between sexes in our cohort, differences in the male-to-female ratio across study populations may skew the overall association. Second, our previous study demonstrated that the intronic *MERTK* rs4374383 variant is associated with *MERTK* expression.^16^ As circulating sol-MerTK levels may reflect both MerTK cleavage and baseline *MERTK* expression, differences in the genetic background across cohorts may also contribute to the discrepant findings. However, genetic data were not available in our cohorts. Therefore, one limitation of our study is that we were unable to adjust for *MERTK* rs4374383 or other potential genetic confounders when assessing the sex-specific associations between circulating sol-MerTK and fibrosis severity. Notably, genetic risk variants may exert sex-specific effects on MASLD progression, as reported for PNPLA3 I148M and HSD17B13 rs72613567.^33^^,^^34^ Sex-specific associations involving *MERTK*-related SNPs have also been reported in Alzheimer’s disease.^35^ In addition, *MERTK* expression differs by sex across multiple human tissues.^36^ These findings highlight an important direction for future studies to determine whether the reported associations of the intronic MERTK SNPs with MERTK expression and liver fibrosis are sex dependent.

Sex differences across different experimental MASLD models may vary depending on the disease-inducing mechanisms and downstream pathways.^37^ While young adult female mice generally exhibit lower susceptibility than age-matched males to disease phenotypes in most experimental MASLD models,[37], [38], [39], [40] more severe hepatic pathology in females has also been reported in certain experimental settings.^41^ Thus, the selection of experimental models for studies of sex differences in MASLD requires careful consideration. In this study, we sought to experimentally recapitulate the sex-specific patterns observed in our clinical cohorts using the CDAHFD model with different fibrosis severity, in which sex differences in fibrosis have been reported.^39^ Similar to our clinical observations, the CDAHFD models showed sex-specific patterns in serum sol-MerTK levels, which progressively decreased as fibrosis advanced from 4 to 12 weeks in male mice but did not differ in female mice. However, it should be noted that our male and female mouse cohorts were established independently at different times and in different animal facilities, direct male versus female comparisons may not be appropriate, particularly given that environmental factors such as ambient temperature and microbiome can influence sex differences in MASLD susceptibility.^42^ Moreover, our female CDAHFD experiments were conducted in young adult female mice. Future studies using experimental models that more closely recapitulate MASLD progression across different reproductive statuses will be needed.

MerTK cleavage is suppressed in fibrotic compared with steatotic livers from male MASLD mice.^16^ In our male MASLD model, we observed that both hepatic *Mertk* mRNA and serum sol-MerTK levels were significantly higher in CDAHFD-fed male mice than in chow-fed controls. However, serum sol-MerTK levels but not hepatic *Mertk* mRNA levels were decreased as fibrosis progressed. These combined data raise the possibility that circulating sol-MerTK levels are initially increased in early MASLD due to enhanced *Mertk* transcription, whereas their subsequent decline in progressive fibrosis may reflect impaired cleavage in fibrotic livers. Therefore, the decreased MerTK cleavage may represent a key mechanism underlying the progressive decline in circulating sol-MerTK levels observed in advancing liver fibrosis in males.

Postmenopausal women have a higher risk of MASLD and are more likely to develop advanced hepatic fibrosis than premenopausal women.^11^ The postmenopausal decline in estrogen is considered to be a key factor contributing to the increased susceptibility to MASLD.^8^ Our observation that circulating sol-MerTK levels were markedly lower in postmenopausal than in premenopausal women suggests that estrogen may regulate sol-MerTK production. Although this interpretation is limited by the small number of premenopausal women in our cohort, findings from our *in vivo* 17β-estradiol implantation mouse model support the likelihood that estrogen induces sol-MerTK production. Moreover, our *in vitro* experiments suggest that 17β-estradiol may promote sol-MerTK release from BMDMs in a dose-dependent manner, potentially through enhanced MerTK cleavage. It remains to be determined whether MerTK cleavage is specifically regulated by 17β-estradiol or more broadly by other estrogens, and whether this regulation occurs only in macrophages. Given that estrogen regulates MerTK cleavage in a dose-dependent manner, the observed non-association between circulating sol-MerTK and fibrosis severity in women with MASLD may be confounded by inter-individual variations in estrogen levels. However, estrogen data were not available in our cohort. We alternatively stratified women by menopausal status and observed no significant association between circulating sol-MerTK and fibrosis severity in either premenopausal or postmenopausal women. However, the premenopausal subgroup analysis was limited by the small sample size. Therefore, future studies in larger, well-characterized clinical cohorts with detailed reproductive status information and circulating estrogen measurements will be needed to clarify this relationship. Our previous study has demonstrated that MerTK cleavage plays a protective role against liver fibrosis in MASLD.^16^ Thus, the estrogen–MerTK axis may represent one plausible mechanism underlying sex differences in MASLD-related fibrosis. However, further *in vivo* hormonal manipulation and loss-of-function experiments are required to determine whether this axis functionally contributes to sex differences in fibrosis progression in MASLD. Nevertheless, our results highlight the importance of considering sex differences in future investigations of MerTK-related studies in MASLD.

In summary, from a translational perspective, our study highlights the need to integrate sex as a core biological variable in the design of preclinical studies, clinical trials, and biomarker development strategies in MASLD. In recent years, sex disparities in MASLD trial enrollment have improved;^12^ however, important gaps remain in sex-aware study design and sex-stratified analysis.^13^ Our findings also support emerging recommendations to consider reproductive status as an additional layer of biological heterogeneity in study design and analysis. The enrollment of women of reproductive age in clinical trials is often subject to additional restrictions because of ethical and safety concerns.^13^^,^^43^ These necessary safeguards may limit the representation of women across different reproductive and hormonal states, potentially overlooking biological differences that may affect therapeutic efficacy, drug metabolism, and safety profiles. Thus, future preclinical MASLD studies and clinical observational cohort studies should first adopt more rigorous sex-aware designs and incorporate reproductive status and hormonal exposure into both study design and analysis. In interventional trials, efforts should be made to improve representation of women across reproductive status whenever ethically justifiable and clinically safe. Moreover, sex- and reproductive status–stratified analyses and reporting are encouraged to improve the generalizability of trial findings and to support precision medicine in MASLD.

## Authors’ contributions

B. Cai., Y.Z., and N.G. designed the study; Y.Z. and N.G. performed experiments; B. Cogliati performed histological analysis in mouse models; M.B.B., S.L.F., X.Z., A.L., and Y.H. provided serum samples and clinical data from the Mount Sinai MASLD/MASH biobank; A.N., S.B.H., R.S., and J.N.B provided serum samples and clinical data from the UCLA CLRC Human MASLD Core; Z. Z. provided serum samples from the 17β-estradiol pellet implantation mouse model; Y.Z. and B. Cai. integrated and analyzed the data and drafted the manuscript; and all the coauthors participated in the manuscript editing.

## Data availability

All data are available from the corresponding authors upon reasonable request.

## Financial support

This work was supported in part by 10.13039/100000002NIH grants R01DK134610, R01HL167107, and R35GM147269, the Irma T. Hirschl/10.13039/100011331Monique Weill-Caulier Trust Research Award (to B. Cai.); R01DK128289 (to S.L.F.); R01DK125354 (to Z.Z.); K08DK136706 (to A.N.); IK2CX002593 (to J.N.B.); UL1TR004419 (Clinical translational Science Award to 10.13039/100007277Icahn School of Medicine at Mount Sinai); Mount Sinai MASH Center of Excellence (to M.B.B.).

## Conflict of interest

The authors declare no conflicts of interest.

Please refer to the accompanying ICMJE disclosure forms for further details.
