# Supplementary material for: Sexual dimorphism in the association of circulating TAM receptors–ligands with MASLD-related fibrosis
Source: JHEP Rep. 2026 Jun 30;8(9):101937. doi: 10.1016/j.jhepr.2026.101937 (PMC13521018; doi:10.1016/j.jhepr.2026.101937)
Supplement: Supplementary file 1 [file mmc1.pdf]

Supplementary data

Sexual dimorphism in the association of circulating TAM receptors–ligands with MASLD-related fibrosis

Yiwei Zhu, Neha Gupta, Bruno Cogliati, Yulu He, Asher Leviton, Jihane N. Benhammou, Steven-Huy B. Han, Zhenqi Zhou, Alexander Nguyen, Xiaotao Zhang, Rajat Singh, Scott L. Friedman, Meena B. Bansal, Bishuang Cai

Table of contents

Supplementary Material and Methods....2

Supplementary Figures....4

Supplementary Tables....10

Supplementary References....14

Supplementary Material and Methods

Mouse liver histology and scoring

Hematoxylin and eosin (H&E) and picrosirius red staining were performed on formalin-fixed, paraffin-embedded (FFPE) sections. Briefly, fresh liver tissue was fixed in 10% neutral buffered formalin overnight and processed for paraffin embedding. FFPE blocks were sectioned at 3–5 µm using a microtome, and sections were mounted onto charged glass slides. Slides were deparaffinized in xylene and rehydrated through a graded ethanol series (100%, 95%, 85%, and 70% ethanol in water). After deparaffinization and rehydration, standard H&E staining was performed, and liver histology was evaluated by a veterinary pathologist blinded to group allocation. Lesions were graded according to the rodent MASLD scoring framework.[1] A global steatosis index was generated for each animal by summing the individual grades for macrovesicular steatosis, microvesicular steatosis, and hepatocellular hypertrophy. Each component was scored on a 0–3 scale, reflecting involvement of <5%, 5–33%, 33–66%, or >66% of the parenchyma, respectively. Lobular inflammatory activity was evaluated by quantifying inflammatory foci per field and assigning severity categories as follows: 0 (<0.5 foci/field), 1 (0.5–1.0 foci/field), 2 (1.0–2.0 foci/field), or 3 (>2.0 foci/field).

For picrosirius red staining, deparaffinized and dehydrated FFPE sections were first incubated for 2 hours in a solution containing 0.01% fast green (Sigma, #F7258) in picric acid (Sigma, #P6744), followed by an additional 2 hours in a solution containing 0.04% fast green and 0.1% sirius red (Sigma, #365548) in picric acid. Slides were then rehydrated and mounted in Permount solution (Fisher, #SP15) for microscopic evaluation. Liver fibrosis was quantified as the percentage of sirius red$^+$ area out of the total area using Fiji software.[2] Quantification was performed on the whole section for all imaging analyses.

ELISA measurement

Levels of sol-MerTK, sol-Axl, Gas6, and Protein S in human serum, mouse serum, and culture supernatants were measured using ELISA kits according to the manufacturers' instructions. Human serum samples were measured using the human ELISA kits (R&D Systems: #DY6488 for sol-MerTK, #DY154 for sol-Axl, and #DY885B for Gas6; Invitrogen: #EEL043 for Protein S). Mouse serum and cell culture supernatants were measured using the mouse ELISA kits (R&D

Systems: #DY591 for sol-MerTK, #DY854 for sol-Axl, and #DY986 for Gas6). Values below the lower limit of detection (LOD) were imputed as one-half of the LOD for statistical analysis.

Quantitative RT-PCR

Total RNA was extracted from mouse liver tissue or cultured cells using the RNeasy Mini Kit (Qiagen, #74106). Reverse transcription was performed with 0.5–1 μg RNA using the High-Capacity cDNA Reverse Transcription kit (Applied Biosystems, #4374967). Quantitative RT-PCR was performed in a volume of 10 μL using iQ SYBR Green Supermix (Bio-Rad, #1708886) and the Light Cycler 480 II Real-Time PCR system (Roche). Primer sequences are shown in Supplementary Table S1.

Isolation and treatment of mouse bone marrow-derived macrophages (BMDMs)

Mouse BMDMs were isolated from 8-to-10-week-old female C57BL/6J mice. Briefly, the mice were euthanized, and the skin was incised to expose the hind limbs. The femur and tibia were dissected by cutting through the hip and ankle joints, and bone marrow was flushed using a syringe fitted with a 26-gauge needle containing BMDM medium. The resulting bone marrow cell suspension was filtered through a 40-μm cell strainer and cultured for 7–10 days in DMEM supplemented with 10% (vol/vol) FBS, 1% penicillin–streptomycin, and 20% (vol/vol) L-929 fibroblast-conditioned medium.

To examine the effect of 17β-estradiol on MerTK *in vitro*, BMDMs were treated with DMSO or 17β-estradiol (10, 25, or 50 nM) for 24 h. Culture supernatants were collected to measure sol-MerTK, and the cells were harvested for RNA extraction.

Supplementary Figures

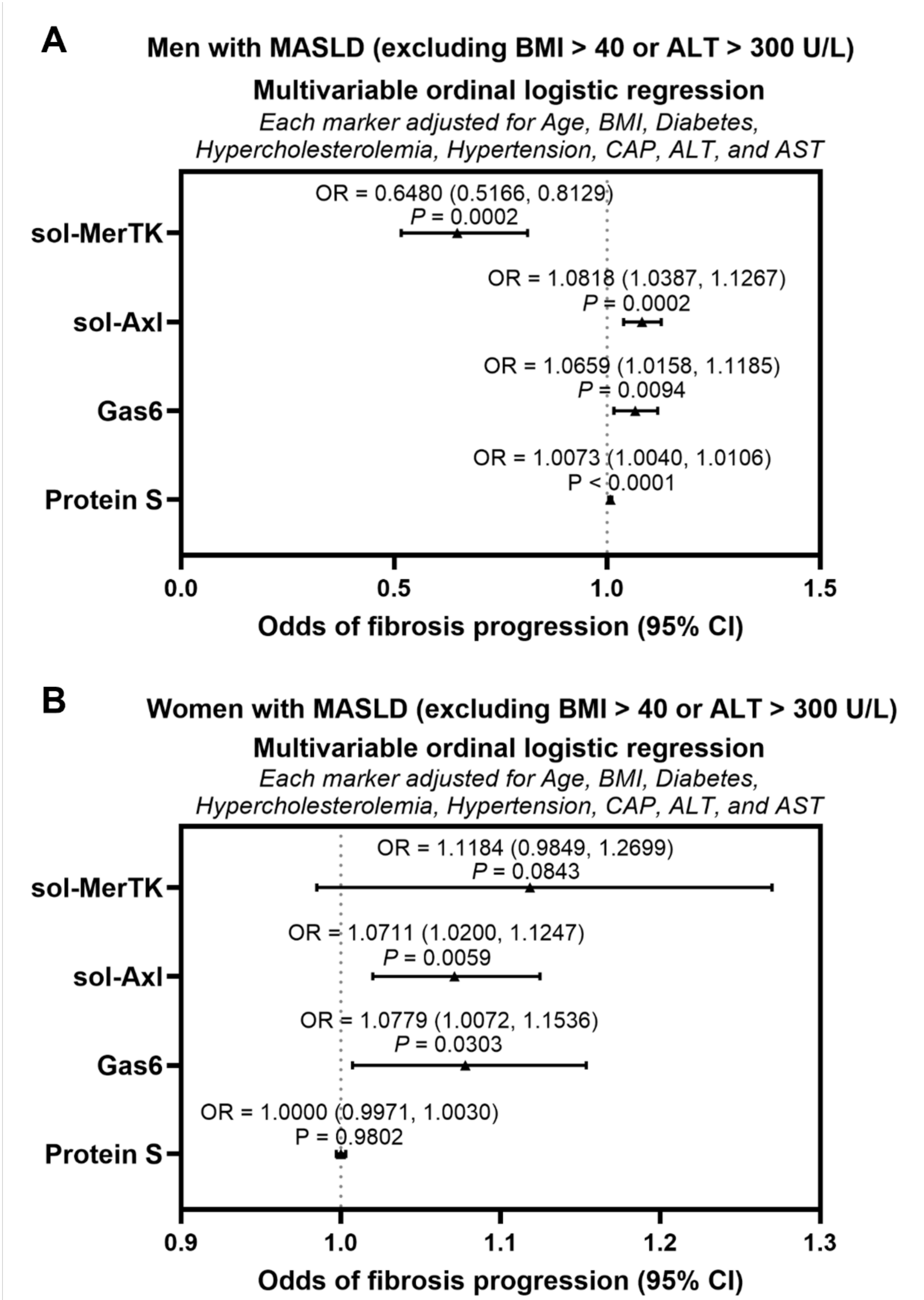

Fig S1. Sensitivity analysis excluding patients with BMI > 40 or ALT > 300 U/L. (A–B) Multivariable ordinal regression analysis showing the associations between liver fibrosis stage

and serum levels of sol-MerTK, sol-Axl, Gas6, and Protein S in men (A) and women (B) with MASLD.

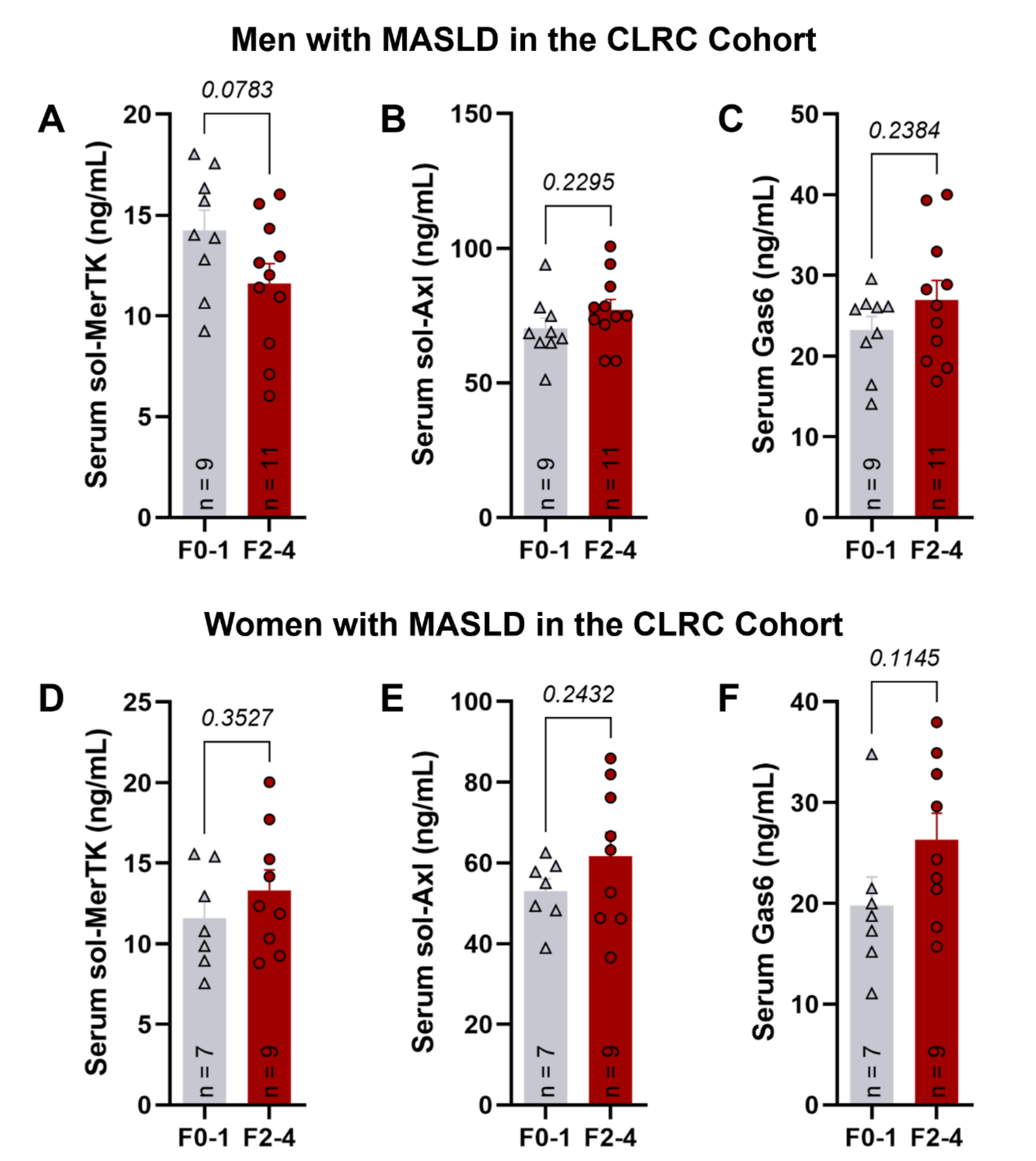

Fig S2. Serum sol-MerTK, sol-Axl, and **Gas6 levels in the CLRC cohort.** (A–C) Serum levels of sol-MerTK (A), sol-Axl (B), and Gas6 (C) in men with MASLD and either F0–1 or F2–4 fibrosis. (D–F) Serum levels of sol-MerTK (D), sol-Axl (E), and Gas6 (F) in women with MASLD and either F0–1 or F2–4 fibrosis. Data are shown as scatter plots with bars representing the mean ± SEM; two-tailed unpaired t test.

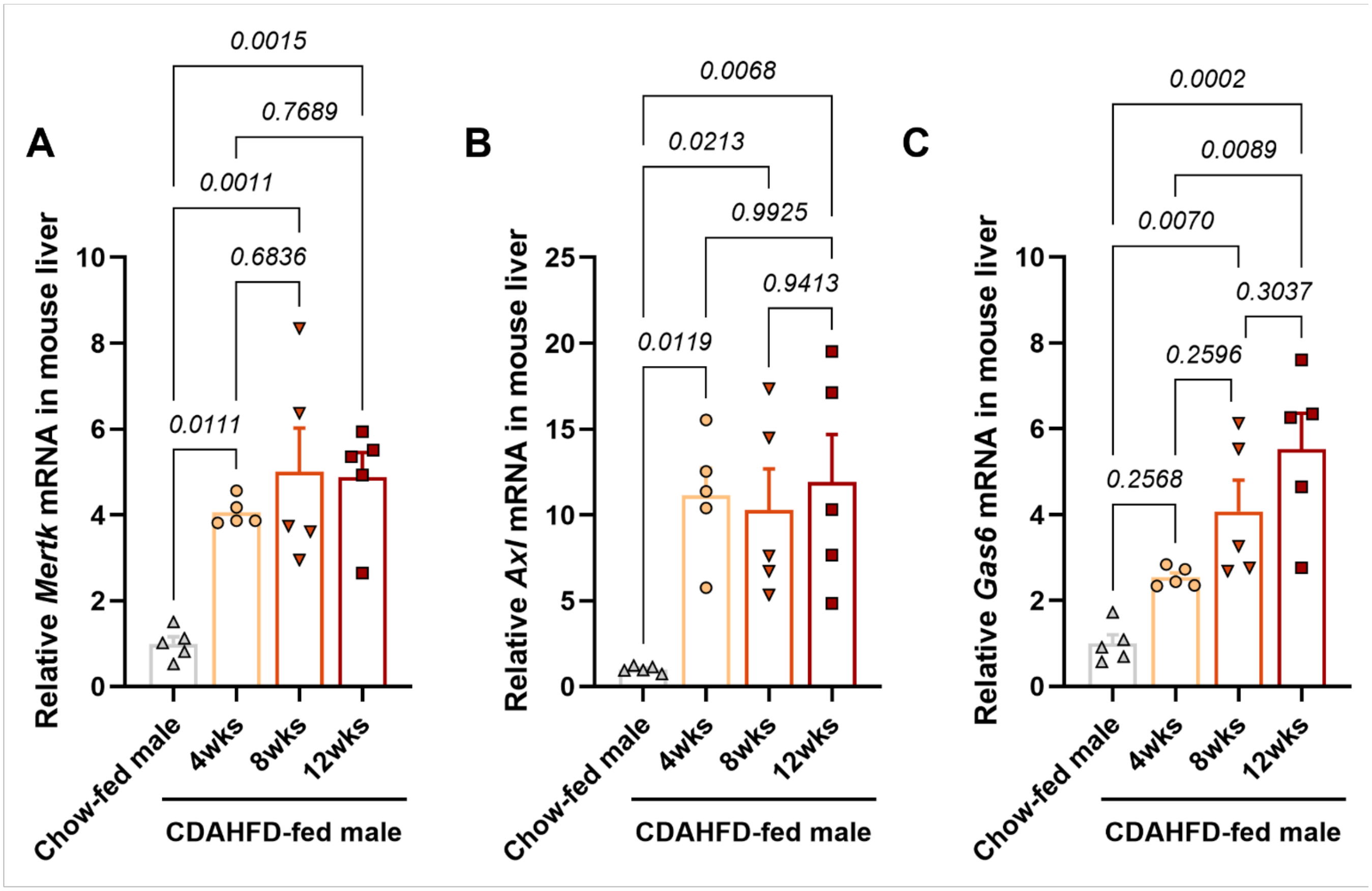

Fig S3. mRNA levels of hepatic *Mertk*, *Axl*, and *Gas6* in the male CDAHFD model. (A–C) mRNA levels of hepatic *Mertk* (A), *Axl* (B), and *Gas6* (C) in chow- or CDAHFD-fed male mice (*n* = 5 per group). Data are shown as scatter plots with bars representing the mean ± SEM; one-way ANOVA followed by Tukey's post hoc test.

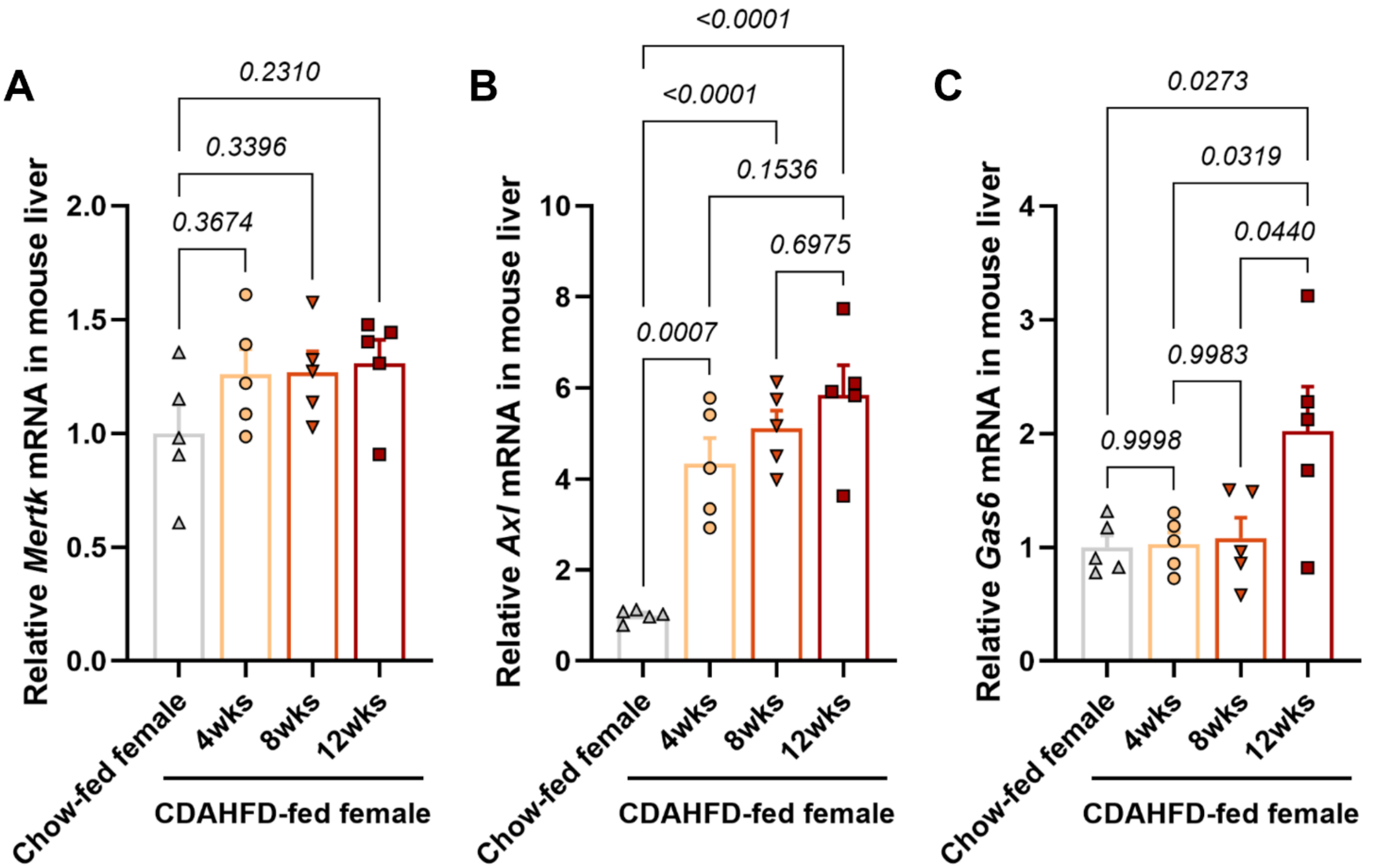

Fig S4. mRNA levels of hepatic *Mertk*, *Axl*, and *Gas6* in the female CDAHFD model. (A–C) mRNA levels of hepatic *Mertk* (A), *Axl* (B), and *Gas6* (C) in chow- or CDAHFD-fed female mice (*n* = 5 per group). Data are shown as scatter plots with bars representing the mean ± SEM; one-way ANOVA followed by Tukey's post hoc test.

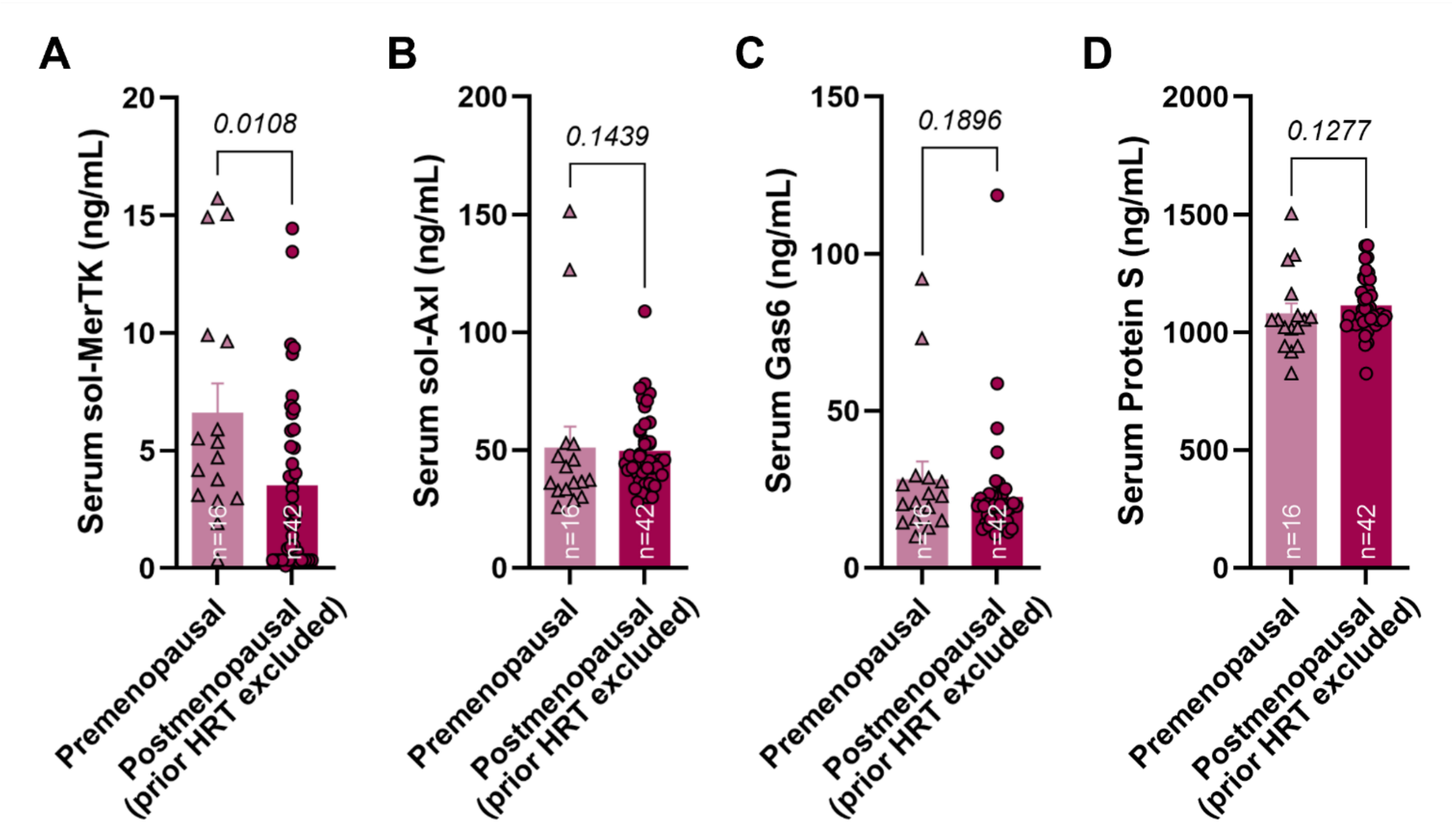

Fig S5. Sensitivity analysis excluding women who had received hormone replacement therapy. (A–D) Serum levels of sol-MerTK (A), sol-Axl (B), Gas6 (C), and Protein S (D) in premenopausal (n = 16) and postmenopausal (n = 42) women with MASLD. Data are shown as scatter plots with bars representing the mean ± SEM; two-tailed Mann-Whitney test.

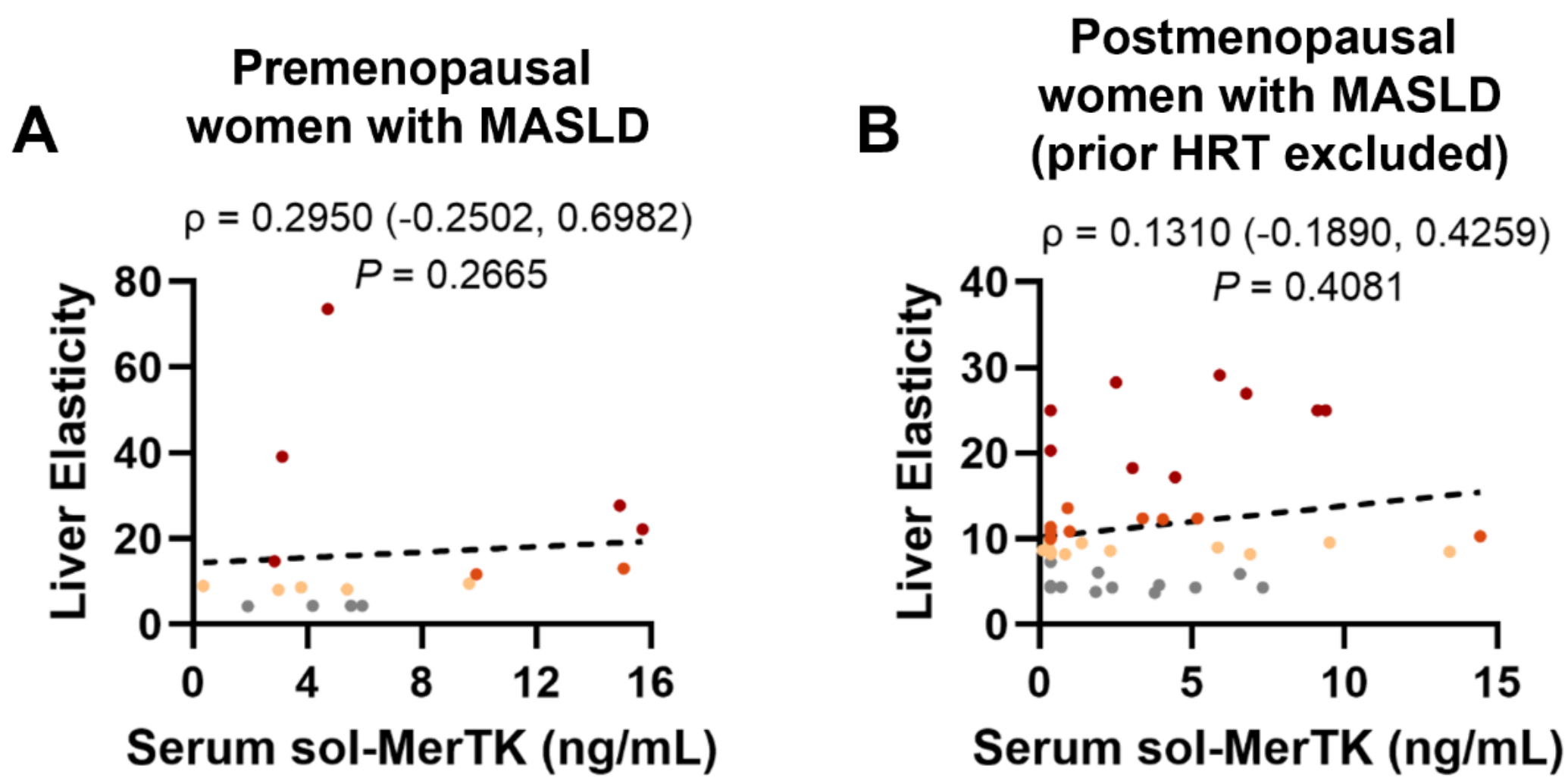

Fig S6. Association between circulating sol-MerTK and liver stiffness in premenopausal and postmenopausal women with MASLD. (A–B) Spearman's correlation between serum sol-MerTK and liver elasticity (kPa) in premenopausal (A; n = 16) and postmenopausal (B; n = 42) women with MASLD excluding who had received hormone replacement therapy.

Supplementary Tables

Table S1. Primers used for qPCR

Gene	Organism	Forward (5' - 3')	Reverse (5' - 3')
Mertk	Mouse	CAGGGCCTTTACCAGGGAGA	TGTGTGCTGGATGTGATCTTC
Axl	Mouse	ATGGCCGACATTGCCAGTG	CGGTAGTAATCCCCGTTGTAGA
Gas6	Mouse	TGCTGGCTTCCGAGTCTTC	CGGGGTCGTTCTCGAACAC

Mertk, MER proto-oncogene tyrosine kinase; Axl, AXL receptor tyrosine kinase; Gas6, growth arrest–specific 6.

Table S2. Baseline characteristics by sex and fibrosis severity in the CLRC Cohort

	Male (*n* = 20)		Female (*n* = 16)	
	**F0–F1 (*n* = 9)**	**F2–F4 (*n* = 11)**	**F0–F1 (*n* = 7)**	**F2–F4 (*n* = 9)**
**Fibrosis diagnosis, *n* (Stage)**	1 (F0), 6 (F0–F1), 2 (F1)	4 (F2), 3 (F2–3), 2 (F3), 2 (F4)	7 (F0–F1)	2 (F2–F3), 3 (F3), 2 (F3–F4), 2 (F4)
FibroScan	6 (F0–F1)	2 (F2), 2 (F3), 1 (F4)	5 (F0–F1)	1 (F3), 1 (F4)
Liver Biopsy	1 (F0), 2 (F1)	2 (F2), 2 (F2–3), 1 (F4)	-	2 (F2–F3), 2 (F3), 2 (F3–F4)
MRE	-	1 (F2–F3)	2 (F0–F1)	1 (F4)
**Continuous variables, mean (SD)**				
Age, years	45.6 (17.5)	59.4 (15.0)	58.1 (11.3)	55.9 (17.0)
BMI, kg/m^2	30.7 (3.5)	31.1 (4.4)	29.7 (7.9)	32.0 (6.1)
ALT, U/L	71.4 (57.0)	77.6 (56.5)	33.9 (14.7)	67.4 (37.3)
AST, U/L	44.6 (34.0)	50.6 (26.6)	28 (7.0)	50.4 (20.8)
**Comorbidities, *n* (%)**				
Obesity				
Yes	6 (66.7%)	7 (63.6%)	3 (42.9%)	3 (33.3%)
No	3 (33.3%)	4 (36.4%)	4 (57.1%)	6 (66.7%)
Diabetes				
Yes	3 (33.3%)	6 (54.5%)	5 (71.4%)	7 (77.8%)
No	6 (66.7%)	5 (45.5%)	2 (28.6%)	2 (22.2%)
Hypertension				
Yes	5 (55.6%)	8 (72.7%)	5 (71.4%)	6 (66.7%)
No	4 (44.4%)	3 (27.3%)	2 (28.6%)	3 (33.3%)
Hypercholesterolemia				
Yes	6 (66.7%)	8 (72.7%)	5 (71.4%)	6 (66.7%)
No	3 (33.3%)	3 (27.3%)	2 (28.6%)	3 (33.3%)
Kidney disease				
Yes	0 (0.0%)	1 (9.1%)	0 (0.0%)	2 (22.2%)
No	9 (100.0%)	10 (90.9%)	7 (100.0%)	7 (77.8%)
**Ethnicity, *n* (%)**				
Hispanic				
Yes	1 (11.1%)	1 (9.1%)	2 (28.6%)	0 (0.0%)
No	8 (88.9%)	9 (81.8%)	4 (57.1%)	7 (77.8%)

Note: Missing values for continuous variables were as follows: Age, BMI, ALT, and AST had 0% missing. Missing values for categorical variables were as follows: obesity, diabetes, hypertension, hypercholesterolemia, and kidney disease had 0% missing; ethnicity 4 (11.1%).

Table S3. Liver histology scores in Chow- and CDAHFD-fed male mice

Male	Macrovesicular steatosis (0-3)	Microvesicular steatosis (0-3)	Hepatocellular hypertrophy (0-3)	Steatosis Score (0-3)	Inflammatory foci (0-3)	Observations
**Chow**						
**1**	0	0	0	0	0	No fibrosis
**2**	0	0	0	0	0	No fibrosis
**3**	0	0	0	0	0	No fibrosis
**4**	0	0	0	0	0	No fibrosis
**5**	0	0	0	0	0	No fibrosis
**4 weeks of CDAHFD**						
**6**	2	3	3	8	3	Very mild perisinusoidal fibrosis
**7**	3	2	3	8	3	Very mild perisinusoidal fibrosis
**8**	3	2	3	8	3	Very mild perisinusoidal fibrosis
**9**	2	3	2	7	3	Very mild perisinusoidal fibrosis
**10**	3	2	3	8	3	Very mild perisinusoidal fibrosis
**8 weeks of CDAHFD**						
**11**	3	1	3	7	3	Moderate perisinusoidal fibrosis
**12**	3	1	3	7	3	Moderate perisinusoidal fibrosis
**13**	3	1	3	7	3	Moderate perisinusoidal fibrosis
**14**	3	2	3	8	3	Moderate perisinusoidal fibrosis
**15**	3	1	3	7	3	Moderate perisinusoidal fibrosis
**12 weeks of CDAHFD**						
**16**	3	2	3	8	3	Intense perisinusoidal fibrosis
**17**	3	1	3	7	3	Intense perisinusoidal fibrosis
**18**	3	1	3	7	3	Intense perisinusoidal fibrosis
**19**	3	1	3	8	3	Intense perisinusoidal fibrosis
**20**	3	1	3	7	3	Intense perisinusoidal fibrosis

Table S4. Liver histology scores in Chow- and CDAHFD-fed female mice

Female	Macrovesicular steatosis (0-3)	Microvesicular steatosis (0-3)	Hepatocellular hypertrophy (0-3)	Steatosis Score (0-3)	Inflammatory foci (0-3)	Observations
**Chow**						
**1**	0	0	0	0	0	No fibrosis
**2**	0	0	0	0	0	No fibrosis
**3**	0	0	0	0	0	No fibrosis
**4**	0	0	0	0	0	No fibrosis
**5**	0	0	0	0	0	No fibrosis
**4 weeks of CDAHFD**						
**6**	3	0	3	6	3	Very mild perisinusoidal fibrosis
**7**	3	1	3	7	3	Very mild perisinusoidal fibrosis
**8**	3	1	3	7	3	Very mild perisinusoidal fibrosis
**9**	2	1	3	7	3	Very mild perisinusoidal fibrosis
**10**	3	1	3	7	3	Very mild perisinusoidal fibrosis
**8 weeks of CDAHFD**						
**11**	3	0	3	6	2	Moderate perisinusoidal fibrosis
**12**	3	1	3	7	3	Moderate perisinusoidal fibrosis
**13**	3	0	3	6	2	Mild perisinusoidal fibrosis
**14**	3	1	3	7	3	Moderate perisinusoidal fibrosis
**15**	3	1	3	7	3	Moderate perisinusoidal fibrosis
**12 weeks of CDAHFD**						
**16**	3	2	3	8	3	Intense perisinusoidal fibrosis
**17**	3	1	3	7	3	Intense perisinusoidal fibrosis
**18**	3	1	3	7	3	Intense perisinusoidal fibrosis
**19**	3	1	3	7	3	Intense perisinusoidal fibrosis
**20**	3	1	3	7	3	Intense perisinusoidal fibrosis

Supplementary References

[1] Liang W, Menke AL, Driessen A, et al. Establishment of a general NAFLD scoring system for rodent models and comparison to human liver pathology. PLoS One. 2014;9:e115922.

[2] Schindelin J, Arganda-Carreras I, Frise E, et al. Fiji: an open-source platform for biological-image analysis. Nat Methods. 2012;9:676-682.
