# Supplementary material for: Sexual dimorphism in the association of circulating TAM receptors–ligands with MASLD-related fibrosis
Source: JHEP Rep. 2026 Jun 30;8(9):101937. doi: 10.1016/j.jhepr.2026.101937 (PMC13521018; doi:10.1016/j.jhepr.2026.101937)
Supplement: Supplementary file 2 [file mmc2.pdf]

JHEP Reports

CTAT methods

Tables for a "Complete, Transparent, Accurate and Timely account" (CTAT) are now mandatory for all revised submissions. The aim is to enhance the reproducibility of methods.

- Only include the parts relevant to your study
- Refer to the CTAT in the main text as 'Supplementary CTAT Table'
- Do not add subheadings
- Add as many rows as needed to include all information
- Only include one item per row

If the CTAT form is not relevant to your study, please outline the reasons why:

N/A

1.1 Antibodies

Name	Citation	Supplier	Cat no.	Clone no.

1.2 Cell lines

Name	Citation	Supplier	Cat no.	Passage no.	Authentication test method

1.3 Organisms

Name	Citation	Supplier	Strain	Sex	Age	Overall n number
Mouse	N/A	The Jackson Laboratory	C57BL/6J	Male	6-8 weeks	25
Mouse	N/A	The Jackson Laboratory	C57BL/6J	Female	6-8 weeks	25

1.4 Sequence based reagents

Name	Sequence	Supplier
Mertk forward	CAGGGCCTTTACCAGGGAGA	Integrated DNA Technologies
Mertk reverse	TGTGTGCTGGATGTGATCTTC	Integrated DNA Technologies
Axl forward	ATGGCCGACATTGCCAGTG	Integrated DNA Technologies
Axl reverse	CGGTAGTAATCCCCGTTGTAGA	Integrated DNA Technologies
Gas6 forward	TGCTGGCTTCCGAGTCTTC	Integrated DNA Technologies
Gas6 reverse	CGGGGTCGTTCTCGAACAC	Integrated DNA Technologies

1.5 Biological samples

Description	Source	Identifier
153 serum samples from patient with MASLD	Mount Sinai MASLD/MASH Registry and Biobank	N/A
36 serum samples from patient with MASLD	UCLA Comprehensive Liver Research Center Human MASLD Core	N/A

1.6 Deposited data

Name of repository	Identifier	Link

1.7 Software

Software name	Manufacturer	Version
Fiji	NIH/Macintosh	ImageJ 1.54p
GraphPad Prism	GraphPad Software Inc	Version 11.0.1
R	R Foundation for Statistical Computing	Version 4.5.1
RStudio	Posit Software	2026.01.1 Build 403

1.8 Other (*e.g.* drugs, proteins, vectors etc.)

L-Amino Acid Diet With 60 kcal% Fat With 0.1% Methionine and No Added Choline (CDAHFD)	Research diets	A06071302
17β-Estradiol	Sigma Aldrich	E8875
17β-estradiol pellets	Innovative Research of America	E-121

1.9 Please provide the details of the corresponding methods author for the manuscript:

Bishuang Cai: bishuangcai@mednet.ucla.edu

2.0 Please confirm for randomised controlled trials all versions of the clinical protocol are included in the submission. These will be published online as supplementary information.

N/A
