# Supplementary material for: Sexual dimorphism in the association of circulating TAM receptors–ligands with MASLD-related fibrosis
Source: JHEP Rep. 2026 Jun 30;8(9):101937. doi: 10.1016/j.jhepr.2026.101937 (PMC13521018; doi:10.1016/j.jhepr.2026.101937)
Supplement: Supplementary file 4 [file mmc4.pdf]

Research article

JHEP|Reports

Sexual dimorphism in the association of circulating TAM receptors–ligands with MASLD-related fibrosis

Authors

Yiwei Zhu, Neha Gupta, Bruno Cogliati, …, Scott L. Friedman, Meena B. Bansal, Bishuang Cai

Correspondence

BishuangCai@mednet.ucla.edu (B. Cai).

Graphical abstract

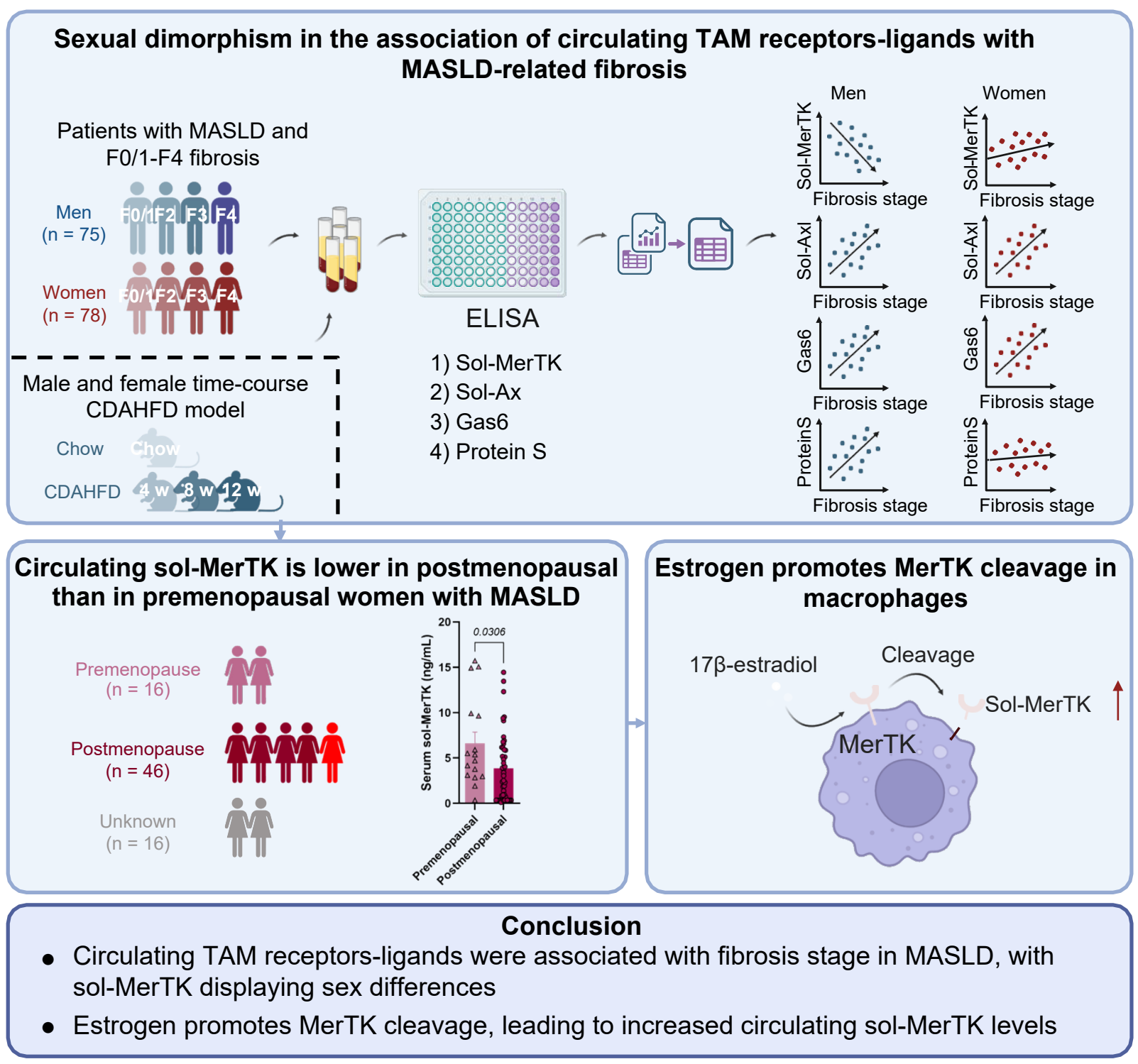

Highlights:

- Circulating sol-MerTK is negatively associated with fibrosis stages in men with MASLD but not in women.
- Circulating sol-Axl and Gas6 are positively associated with fibrosis stages in both sexes.
- Premenopausal women with MASLD have higher circulating sol-MerTK than postmenopausal women.
- Estrogen increases circulating sol-MerTK levels in mice and promotes MerTK cleavage in macrophages.

Impact and implications:

MASLD-related fibrosis is progressive and sexually dimorphic. Our findings reveal sex-specific associations of circulating sol-MerTK and Protein S with liver fibrosis stages in MASLD, highlighting the need to incorporate sex as a biological variable in MerTK- and Protein S–related studies. In contrast, circulating sol-Axl and Gas6 were positively associated with liver fibrosis stages in both sexes, suggesting their potential as sex-independent indicators of fibrosis progression in MASLD. We further show that circulating sol-MerTK levels are higher in premenopausal than in postmenopausal women, and that estrogen increases circulating sol-MerTK levels in mice and promotes MerTK cleavage in macrophages. Given the reported roles of estrogen and MerTK cleavage in MASLD-related fibrosis, our study provides potential mechanistic insights into sex differences in MASLD-related fibrosis.

https://doi.org/10.1016/j.jhepr.2026.101937
© 2026 The Author(s). Published by Elsevier B.V. on behalf of European Association for the Study of the Liver (EASL). This is an open access article under the CC BY license (http://creativecommons.org/licenses/by/4.0/).

Sexual dimorphism in the association of circulating TAM receptors–ligands with MASLD-related fibrosis

Yiwei Zhu[1,2,3,†], **Neha Gupta**[1,2,3,†], Bruno Cogliati[3], Yulu He[3], Asher Leviton[3], Jihane N. Benhammou[1,2], Steven-Huy B. Han[1,2], Zhenqi Zhou[4], Alexander Nguyen[1,2], Xiaotao Zhang[3], Rajat Singh[1,2], Scott L. Friedman[3], Meena B. Bansal[3], Bishuang Cai[1,2,3,*]

JHEP Reports **2026**. vol. 8 | 1–12

Check for updates

Background & Aims: The TAM receptors (MerTK and Axl) and their ligands (Gas6 and Protein S) have been implicated in MASLD-related fibrosis. However, whether circulating levels are associated with fibrosis stage and whether these associations differ by sex remain unclear. This study aimed to characterize circulating TAM receptors and ligands across fibrosis stages in men and women with MASLD.

Methods: Circulating TAM receptors and ligands were measured in men (n = 75) and women (n = 78) with MASLD and analyzed for associations with fibrosis stage. Time-course choline-deficient, amino acid-defined, high-fat diet mouse models were established in male and female mice to assess circulating TAM members. These markers were further compared between premenopausal and postmenopausal women with MASLD. In addition, circulating soluble MerTK (sol-MerTK) was measured in mice treated with 17β-estradiol. *In vitro*, macrophages were treated with 17β-estradiol, and sol-MerTK and Mertk mRNA levels were measured.

Results: In men with MASLD, circulating sol-MerTK was inversely associated with fibrosis stage (adjusted ordinal regression: odds ratio 0.63; 95% CI 0.51–0.78; p <0.0001) and decreased with fibrosis progression in male mice. In contrast, sol-MerTK showed no significant association with fibrosis stage in women (odds ratio 1.12; 95% CI 1.00–1.25; p = 0.0543) or female mice. Higher circulating sol-Axl and Gas6 levels were associated with more advanced fibrosis in both men and women with MASLD. Circulating sol-MerTK levels were higher in premenopausal than postmenopausal women (p = 0.0306). Treatment with 17β-estradiol increased circulating sol-MerTK levels in mice (p = 0.0119). In macrophages, 17β-estradiol increased sol-MerTK release in a dose-dependent manner (p <0.05), with a non-significant trend toward reduced *Mertk* mRNA expression (p >0.05).

Conclusions: Circulating TAM receptors and ligands were associated with fibrosis stage in MASLD, with sex-specific associations observed for sol-MerTK. Furthermore, 17β-estradiol promoted MerTK cleavage, providing a potential mechanism underlying these sex differences.

Clinical trial number: N/A.

© 2026 The Author(s). Published by Elsevier B.V. on behalf of European Association for the Study of the Liver (EASL). This is an open access article under the CC BY license (http://creativecommons.org/licenses/by/4.0/).

Introduction

Metabolic dysfunction-associated steatotic liver disease (MASLD) is the most prevalent chronic liver condition worldwide, affecting approximately 25% of the general population, and is the leading indication for liver transplantation in women.[1,2] MASLD encompasses a disease spectrum ranging from simple steatosis to metabolic dysfunction-associated steatohepatitis (MASH) and can progress to liver fibrosis. Liver fibrosis is the major determinant of adverse clinical outcomes including hepatocellular carcinoma, making it the central focus of both preclinical research and therapeutic development in MASLD.[3] Staged from F0 (no fibrosis) to F4 (cirrhosis), liver fibrosis stage ≥ F2 (considered significant fibrosis) is associated with a higher risk of morbidity and mortality, and therefore clinical trials in MASLD have largely focused on patients with fibrosis stage ≥ F2.[4–6] Natural history studies show that fibrosis progression in MASLD is highly heterogeneous: Although many individuals progress slowly, taking approximately seven years to advance by one fibrosis stage, up to 20 percent may be classified as ‘‘rapid progressors” who develop cirrhosis within a decade.[7] However, it is unclear why some patients progress rapidly whereas others remain stable for years. The divergence in clinical outcomes reflects a complex interplay among metabolic stress, inflammatory responsiveness, immune cell activation, and fibrogenesis that vary between individuals. Elucidating the mediators associated with fibrosis stages in MASLD is therefore essential for understanding the mechanisms driving

* Corresponding author. Address: Vatche and Tamar Manoukian Division of Digestive Diseases, Department of Medicine, David Geffen School of Medicine, University of California, Los Angeles, CA, USA.
E-mail address: BishuangCai@mednet.ucla.edu (B. Cai).
† These authors contributed equally.
https://doi.org/10.1016/j.jhepr.2026.101937

fibrosis progression, identifying patients with at-risk MASLD, and guiding the development of more effective and individualized anti-fibrotic therapies in MASLD.

MASLD is a sex-dimorphic disease, with its development and progression influenced by sex chromosomes and sex hormones.[8] In general, men show a higher prevalence of MASLD than women.[9,10] Premenopausal women have a lower risk of developing MASLD and fibrosis progression compared with men; however, after menopause, the loss of estrogen-mediated protection is associated with a comparable or even higher prevalence of advanced hepatic fibrosis in women.[11] Despite the well-recognized sexual dimorphism in MASLD development, progression, and outcomes, our understanding of the mechanisms underlying sex differences in MASLD remains limited. Moreover, sex remains insufficiently considered as a biological variable in the design of preclinical and clinical studies.[8] Although a recent systematic review showed meaningful improvement in the representation of women in MASLD clinical trials,[12] balanced enrollment alone does not ensure sex-aware study design or sex-stratified analysis.[8,13] In addition, experimental studies and the development of new disease models have predominantly relied upon male animals.[14,15] These limitations may have contributed to discrepancies in study outcomes and may hinder the translation of effective therapies, particularly for women. Therefore, incorporating sex as a biological variable in MASLD-related research is essential to enhance the translational relevance of preclinical findings and ensuring equitable diagnostic and therapeutic strategies for both sexes.

TAM (Tyro3, Axl, and MerTK) receptor tyrosine kinases are recognized as important regulators of fibrosis.[16–19] By engaging their ligands Gas6 and Protein S, TAM receptors, particularly Axl and MerTK, activate downstream signaling pathways that exacerbate hepatic fibrogenesis in MASLD.[16,20] The essential roles of TAM receptors in liver fibrosis are further supported by human genome-wide association studies (GWAS), which have linked two intronic single nucleotide polymorphisms (SNPs) in MERTK, rs4374383 and rs6726639, to liver fibrosis in patients with MASLD and HCV.[21–23] MerTK and Axl ectodomains undergo ADAM metallopeptidase-mediated cleavage, generating soluble MerTK (sol-MerTK) and soluble Axl (sol-Axl). Blocking MerTK cleavage in cleavage-resistant MerTK mutant mice has been shown to exacerbate fibrosis in MASLD.[16] Moreover, circulating levels of TAM components have been linked to liver fibrosis in MASLD. However, the circulating patterns of TAM receptors and their ligands across liver fibrosis stages in MASLD remain poorly understood. Furthermore, it remains unknown whether their functions and circulating levels differ by sex in the setting of MASLD. Therefore, this study aimed to characterize circulating levels of TAM receptors and their ligands across fibrosis stages in MASLD and to define their associations with fibrosis severity, with analyses stratified by sex.

In this study, we characterized significant associations between circulating TAM receptors and their ligands and liver fibrosis severity, with circulating sol-MerTK exhibiting sex-specific associations with fibrosis severity in MASLD. Specifically, circulating sol-MerTK was negatively associated with fibrosis stage in men with MASLD and male MASLD mice, but showed no significant association in women with MASLD and female MASLD mice. We further made a novel observation that circulating sol-MerTK levels were higher in premenopausal than postmenopausal women with MASLD. Consistently, 17β-estradiol-treated mice had higher circulating sol-MerTK levels than controls. Finally, we demonstrated that estrogen induced MerTK cleavage, leading to increased sol-MerTK released from macrophages. Overall, our findings highlight the estrogen–MerTK axis as a plausible determinant of underlying sex differences in MASLD-related fibrosis.

Material and methods

Study cohort

The study included two independent study cohorts. The main cohort included 153 patients enrolled in the Mount Sinai MASLD/MASH Registry and Biobank.[24,25] The CLRC cohort included 36 patients from the UCLA Comprehensive Liver Research Center Human MASLD Core. The Mount Sinai MASLD/MASH Registry and Biobank is an ongoing, longitudinal prospective cohort study. Eligible participants were identified from the Mount Sinai Hepatology Practice based on a clinical diagnosis of MASLD. Patients were approached for consent during routine clinical visits. Exclusion criteria included active hepatitis C virus (HCV) infection, untreated hepatitis B virus (HBV) infection, excessive alcohol use, or other chronic liver disease etiologies, ensuring a homogeneous cohort focused on MASLD. Upon enrollment, comprehensive baseline data were collected, including demographics, lifestyle factors, comorbidities, detailed metabolic profiling, and a broad panel of laboratory parameters. Exposure to alcohol was quantitatively assessed using the Alcohol Use Disorders Identification Test (AUDIT-C) along with a detailed review of the electronic health record (EHR). Liver stiffness was assessed by vibration-controlled transient elastography (VCTE), and fibrosis staging was defined as F0 to F1 (<8 kPa), F2 (8 to <10 kPa), F3 (10 to <14 kPa), and F4 (≥14 kPa). Among the 153 patients included from the Mount Sinai Biobank, 14 underwent liver biopsy within 1 year of the baseline date, 30 had biopsies performed more than one year apart from the baseline, and 109 had no biopsy data available. All the studies with human serum samples were approved by Institutional Review Boards (IRB) at the Icahn School of Medicine at Mount Sinai and UCLA.

Animal model

To induce MASLD, 6–8-week-old male and female wild-type C57BL/6J mice (Jackson Laboratory, #000664) were randomly assigned to receive either a chow diet or a choline-deficient, L-amino acid–defined, high-fat diet (CDAHFD; Research Diets, #A06071302) for 4, 8, or 12 weeks (n = 5 per group). The male and female cohorts were established independently, with the male cohort was generated at the Icahn School of Medicine at Mount Sinai and the female cohort was generated at UCLA. For *in vivo* estrogen supplementation, male mice underwent subcutaneous implantation of 17β-estradiol pellets (0.001 mg/pellet; Innovative Research of America, #E-121) for 21 days. Age-matched male mice were used as controls. Food and water were provided *ad libitum*. Mice were housed in a specific pathogen-free facility under a 12-hour light/dark cycle. All animal procedures were approved by the Institutional Animal Care and Use Committee of the

Icahn School of Medicine at Mount Sinai or by the UCLA Chancellor's Animal Research Committee.

Statistical analysis

Spearman's correlation analysis was used to analyze correlations between two parameters. Statistical significance was evaluated using the Kruskal-Wallis test with Dunn's post hoc correction (Bonferroni corrected), one-way ANOVA followed by Tukey's post hoc test, the two-tailed Mann-Whitney test, or the two-tailed unpaired t-test, as appropriate. Continuous variables for patients' baseline characteristics are presented as the mean ± SD. All other results are expressed as the mean ± SEM, and statistical significance was defined as $p < 0.05$. Statistical analyses were performed using GraphPad Prism and R software.

Full details of the materials and methods are provided in the Supplementary Materials.

Results

Circulating TAM receptors and their ligands are associated with liver fibrosis severity in men with MASLD

Serum levels of sol-MerTK, sol-Axl, Gas6, and Protein S were measured in men with MASLD and different degrees of liver fibrosis (n = 75, 17–20 per fibrosis stage; from Mount Sinai cohort) to investigate their associations with fibrosis severity. Baseline characteristics of patients were shown in Table 1. Among male patients in this cohort, CAP score, serum AST levels, and the rate of hypertension differed significantly across fibrosis stages (Table 1). Spearman's correlation analysis showed that serum sol-MerTK levels were negatively correlated with liver stiffness (Fig. 1A), whereas serum sol-Axl, Gas6, and Protein S levels were positively correlated with liver stiffness in men with MASLD (Fig. 1B–D). We further compared serum levels of sol-MerTK, sol-Axl, Gas6, and Protein S across fibrosis stages (F0/F1 to F4) in men with MASLD. Serum sol-MerTK levels gradually decreased with increasing fibrosis stage (Fig. 1E), consistent with our previous finding that MerTK cleavage is suppressed in fibrotic livers compared with steatotic livers in male MASLD mice.[16] In contrast, serum sol-Axl, Gas6, and Protein S levels showed an increasing trend from F0/F1 to F4 and were significantly higher in the F4 stage than in earlier stages (F0/1 and F2; Fig. 1F–H). Moreover, multivariable ordinal regression analysis adjusted for age, BMI, and metabolic comorbidities showed a significant inverse association between serum sol-MerTK levels and fibrosis stage (OR = 0.6032, 95% CI = 0.4876–0.7462, $p <$ 0.0001), indicating that each unit increase in sol-MerTK was associated with a 39.68% reduction in the odds of being in a higher liver fibrosis stage (Fig. 1I, Model 1). Meanwhile, sol-Axl, Gas6, and Protein S levels were positively associated with liver fibrosis stages (Fig. 1I, Model 1). Further adjustment for ALT, AST, and CAP score did not change the observed associations (Fig. 1I, Model 2). In addition, sensitivity analysis excluding patients with extremely high BMI and ALT further supported the robustness of our observations (Supplementary Fig. S1A).

We further validated our findings in the male subset of an independent MASLD cohort (CLRC cohort, Supplementary Table S2). Due to the limited sample size, patients were stratified into early (F0–F1; n = 9) and significant (F2–F4; n = 11) fibrosis groups. Notably, serum sol-MerTK levels exhibited a marginally significant reduction (p = 0.0783) in the F2–F4 stages relative to the F0–F1 stages in men with MASLD (Supplementary Fig. S2A). Serum sol-Axl (p = 0.2295) and Gas6 (p = 0.2384) levels were not significantly elevated (Supplementary Fig. S2B and C), possibly due to the small number of patients with F4 fibrosis (n = 2). These results are consistent with the observations in our main cohort. Overall, our data suggest that lower circulating sol-MerTK and higher sol-Axl, Gas6, and Protein S levels are related to more advanced liver fibrosis in men with MASLD.

Circulating TAM receptors and their ligands are associated with liver fibrosis severity in women with MASLD

We also evaluated serum levels of sol-MerTK, sol-Axl, Gas6, and Protein S in women with MASLD from the Mount Sinai cohort (n = 78, 18–20 per fibrosis stage). Interestingly, in contrast to our findings in men, the serum levels of sol-MerTK showed a borderline positive correlation with liver stiffness in women with MASLD (p = 0.0509, Fig. 2A). Meanwhile, despite an increasing trend from F0/1 to F4, serum sol-MerTK levels did not differ significantly among liver fibrosis stages in women with MASLD (Fig. 2E). In multivariable ordinal regression analysis adjusted for age, BMI, and metabolic comorbidities, we observed a weak positive association between serum sol-MerTK levels and fibrosis stage (OR = 1.1274, 95% CI = 1.0161–1.2508, p = 0.0238; Fig. 2I, Model 1). However, this association was no longer significant following further adjustment for ALT, AST, and CAP (OR = 1.1190, 95% CI = 0.9979–1.2548, p = 0.0543; Fig. 2I, Model 2). We also found a sex difference regarding serum Protein S levels, which showed no association with liver fibrosis severity in women with MASLD (Fig. 2D, H, I). Conversely, serum sol-Axl and Gas6 levels were positively associated with fibrosis severity in women with MASLD (Fig. 2B, C, I), consistent with the findings observed in men with MASLD. Notably, serum sol-Axl and Gas6 levels were significantly higher in the F4 fibrosis stage than in early stages (F0/1–F2; Fig. 2F, G). Sensitivity analysis excluding patients with extremely high BMI and ALT did not alter these observations (Supplementary Fig. S1B). When examined in the female subset from the CLRC cohort, serum sol-MerTK (p = 0.3527), sol-Axl (p = 0.2432), and Gas6 (p = 0.1145) levels did not differ significantly between the F0–F1 (n = 7) and F2–F4 (n = 9) groups (Supplementary Fig. S2D–F). The absence of significant differences in sol-Axl and Gas6 may be due to the limited sample size, particularly patients with F4 fibrosis (n = 2). Taken together, these observations demonstrate sex-specific associations of circulating sol-MerTK and Protein S with liver fibrosis severity in patients with MASLD, while the associations of circulating sol-Axl and Gas6 were consistent between sexes.

Circulating TAM receptors and their ligands are associated with liver fibrosis severity in male and female MASLD mice

To validate the clinical observations, we separately generated time-course CDAHFD models in male mice and female mice to recapitulate MASLD development and fibrosis progression (Fig. 3A and Fig 4A). Histological analyses demonstrated that CDAHFD-fed mice developed hepatic steatosis, inflammation, and fibrosis at all examined time points (4, 8, and 12 weeks)

Table 1. Baseline characteristics by sex and fibrosis stage in main cohort.

	Female (n = 78)					Male (n = 75)				
	F0–F1 (n = 20)	F2 (n = 18)	F3 (n = 20)	F4 (n = 20)	*p* values	F0–F1 (n = 20)	F2 (n = 18)	F3 (n = 17)	F4 (n = 20)	*p* values
**Continuous variables, mean (SD)**										
Liver stiffness, kPa	4.6 (1.0)	8.8 (0.5)	11.7 (1.1)	26.5 (13.1)	<0.0001[a]	5.0 (1.4)	9.1 (0.6)	11.8 (1.1)	26.9 (8.7)	<0.0001[a]
CAP, dB/m	288.8 (52.9)	328.8 (62.7)	326.8 (68.1)	309.9 (69.0)	0.0396[a]	298.7 (49.5)	305.2 (59.4)	275.5 (79.2)	343.7 (39.6)	0.0162[a]
Age, years	58.7 (16.1)	54.4 (13.5)	62.8 (10.7)	59.6 (12.9)	0.3016[a]	48.0 (19.9)	49.5 (16.7)	53.4 (13.9)	55.1 (11.2)	0.9268[a]
BMI, kg/m^2	30.1 (4.8)	33.3 (6.1)	35.1 (9.2)	31.5 (10.2)	0.2235[a]	30.4 (6.2)	34.9 (8.4)	32.8 (4.8)	33.9 (5.7)	0.1805[a]
ALT, U/L	33.2 (17.6)	48.8 (20.6)	28.7 (17.5)	33.9 (12.3)	0.0097[a]	52.7 (47.1)	41.6 (19.7)	51.1 (22.4)	50.9 (23.1)	0.4990[a]
AST, U/L	27.2 (8.6)	36.4 (11.4)	27.0 (9.5)	36.4 (14.6)	0.0085[a]	31.6 (18.8)	32.0 (10.2)	44.4 (21.3)	46.8 (21.7)	0.0035[a]
Systolic BP, mmHg	131.4 (16.6)	127.0 (15.9)	124.9 (14.0)	132.3 (18.8)	0.4351[b]	126.9 (13.0)	121.6 (13.5)	121.7 (13.6)	120.1 (13.2)	0.4082[b]
Diastolic BP, mmHg	75.8 (12.4)	76.6 (7.6)	75.6 (11.4)	77.6 (11.3)	0.9359[b]	79.9 (5.6)	77.6 (9.2)	77.1 (7.1)	74.8 (7.9)	0.2256[b]
**Comorbidities, n (%)**										
Obesity					0.1390[c]					0.7035[c]
Yes	8 (40.0%)	13 (72.2%)	13 (65.0%)	14 (70.0%)		12 (60.0%)	12 (66.7%)	10 (58.8%)	15 (75.0%)	
No	12 (60.0%)	5 (27.8%)	7 (35.0%)	6 (30.0%)		8 (40.0%)	6 (33.3%)	7 (41.2%)	5 (25.0%)	
Diabetes					0.9030[c]					0.5822[c]
Yes	10 (50.0%)	10 (55.6%)	9 (45.0%)	11 (55.0%)		6 (30.0%)	7 (38.9%)	8 (47.1%)	10 (50.0%)	
No	10 (50.0%)	8 (44.4%)	11 (55.0%)	9 (45.0%)		14 (70.0%)	11 (61.1%)	9 (52.9%)	10 (50.0%)	
Hypertension					0.6759[c]					0.0112[c]
Yes	10 (50.0%)	12 (66.7%)	13 (65.0%)	13 (65.0%)		6 (30.0%)	10 (55.6%)	13 (76.5%)	6 (30.0%)	
No	10 (50.0%)	6 (33.3%)	7 (35.0%)	7 (35.0%)		14 (70.0%)	8 (44.4%)	4 (23.5%)	14 (70.0%)	
Hypercholesterolemia					0.2366[c]					0.6259[c]
Yes	8 (40.0%)	11 (61.1%)	12 (60.0%)	7 (35.0%)		5 (25.0%)	8 (44.4%)	6 (35.3%)	8 (40.0%)	
No	12 (60.0%)	7 (38.9%)	8 (40.0%)	13 (65.0%)		15 (75.0%)	10 (55.6%)	11 (64.7%)	12 (60.0%)	
Kidney disease					0.7446[d]					0.7136[d]
Yes	2 (10.0%)	0 (0.0%)	2 (10.0%)	1 (5.0%)		2 (10.0%)	0 (0.0%)	1 (5.9%)	2 (10.0%)	
No	18 (90.0%)	18 (100.0%)	18 (90.0%)	19 (95.0%)		18 (90.0%)	18 (100.0%)	16 (94.1%)	18 (90.0%)	
**Menopausal status, n (%)**					0.8021[d]					
Pre-menopausal	4 (20.0%)	5 (27.8%)	2 (10.0%)	5 (25.0%)						
Post-menopausal	12 (40.0%)	12 (66.7%)	11 (55.0%)	11 (55.0%)						
**Hormone replacement therapy, n (%)**										
Yes	0 (0.0%)	0 (0.0%)	2 (10.0%)	2 (10.0%)						
No	20 (100.0%)	18 (100.0%)	18 (90.0%)	18 (90.0%)						
**Ethnicity, n (%)**					0.2814[d]					0.9615[c]
Hispanic										
Yes	8 (40.0%)	2 (11.1%)	7 (35.0%)	5 (25.0%)		9 (45.0%)	7 (38.9%)	6 (35.3%)	7 (35.0%)	
No	9 (45.0%)	11 (61.1%)	11 (55.0%)	13 (65.0%)		8 (40.0%)	7 (38.9%)	6 (35.3%)	9 (45.0%)	
**Race, n (%)**					0.4858[d]					0.7626[d]
Asian	2 (10.0%)	0 (0.0%)	1 (5.0%)	1 (5.0%)		2 (10.0%)	1 (5.6%)	1 (5.9%)	1 (5.0%)	
Black	2 (10.0%)	1 (5.6%)	3 (15.0%)	0 (0.0%)		3 (15.0%)	4 (22.2%)	1 (5.9%)	0 (0.0%)	
Native	1 (5.0%)	0 (0.0%)	0 (0.0%)	1 (5.0%)		0 (0.0%)	0 (0.0%)	1 (5.9%)	1 (5.0%)	
Other	8 (40.0%)	14 (77.8%)	11 (55.0%)	13 (65.0%)		8 (40.0%)	6 (33.3%)	6 (35.3%)	9 (45.0%)	
White	7 (35.0%)	3 (16.7%)	5 (25.0%)	4 (20.0%)		6 (30.0%)	7 (38.9%)	7 (41.2%)	7 (35.0%)	

Note 1: Missing values for continuous variables were as follows: liver stiffness, age, and BMI had 0% missing; CAP 6 (3.9%), ALT 4 (2.6%), AST 4 (2.6%), systolic blood pressure 1 (0.7%), and diastolic blood pressure 1 (0.7%). Missing values for categorical variables were as follows: obesity, diabetes, hypertension, hypercholesterolemia, kidney disease, and hormone replacement therapy had 0% missing; menopausal status 16 (20.5%), ethnicity 28 (18.3%), and race 5 (3.3%).

Note 2: Statistical significance was determined using:

[a]Kruskal-Wallis test.

[b]one-way ANOVA.

[c]Chi-square test.

[d]Fisher's Exact Test. All p values were calculated based on available data; missing values were not imputed.

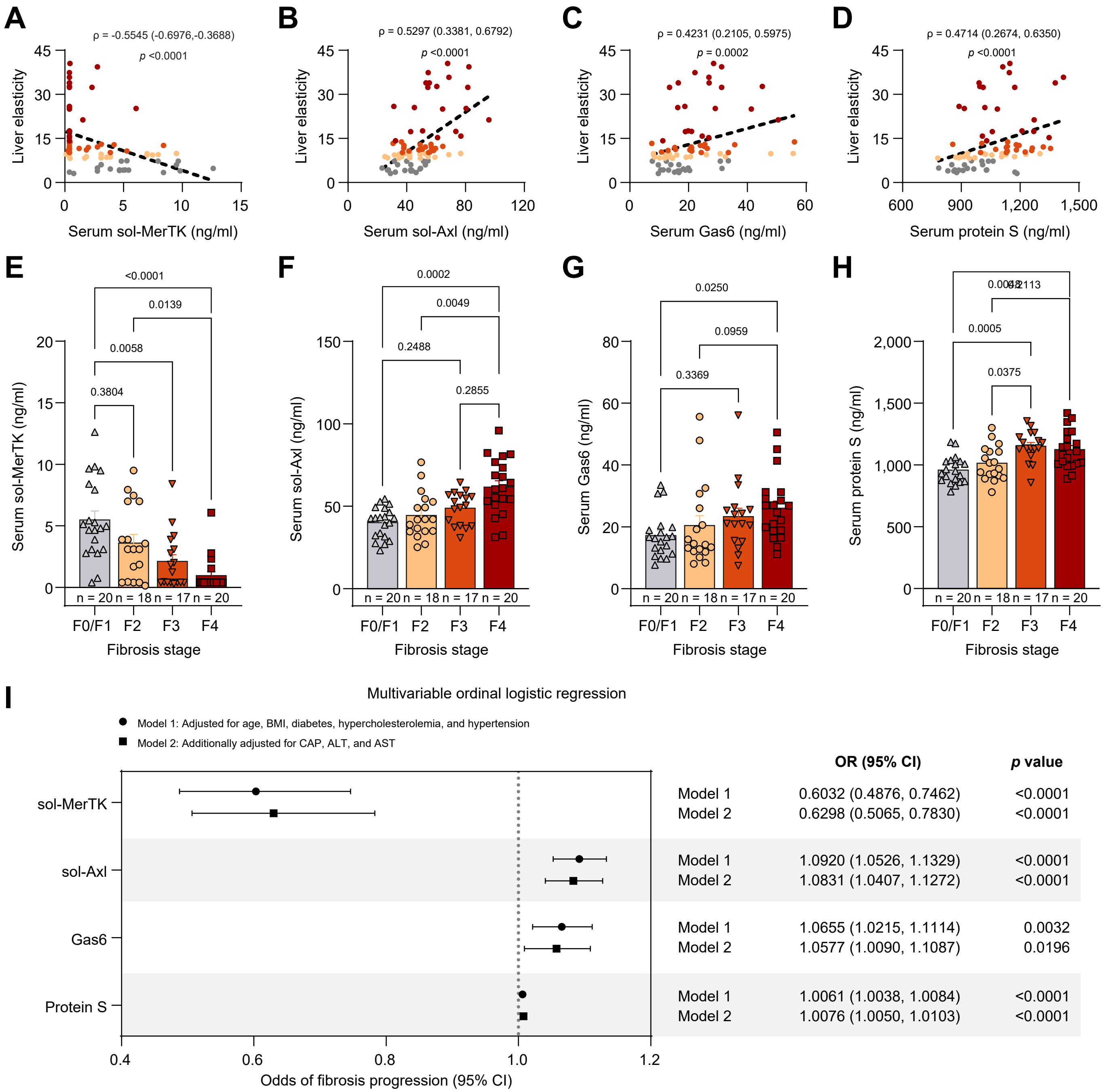

Fig. 1. Serum sol-MerTK, sol-Axl, Gas6, and Protein S levels are associated with liver fibrosis severity in men with MASLD. (A–D) Spearman's correlation analyses showing the correlations between liver elasticity (kPa) and serum sol-MerTK (A), sol-Axl (B), Gas6 (C), and Protein S (D) in men with MASLD (n = 75). (E–H) Serum levels of sol-MerTK (E), sol-Axl (F), Gas6 (G), and Protein S (H) in men with MASLD and different liver fibrosis stages (F0/1–F4; n = 17–20 per group). (I) Multivariable ordinal regression analysis showing the associations between liver fibrosis stage and serum levels of sol-MerTK, sol-Axl, Gas6, and Protein S. Model 1 was adjusted for age, BMI, diabetes, hypercholesterolemia, and hypertension; Model 2 was additionally adjusted for CAP, ALT, and AST. Data are shown as scatter plots with bars representing the mean ± SEM; Kruskal-Wallis with Dunn's post hoc test (Bonferroni corrected).

compared with chow-fed controls in both male (Fig. 3B and Supplementary Table S3) and female (Fig. 4B and Supplementary Table S4) mice model. Of note, hepatic steatohepatitis severity was similar across different time points, whereas fibrosis severity progressively increased with the duration of CDAHFD feeding (Fig. 3B and 4B, Supplementary Tables S3 and S4). Therefore, this CDAHFD model may be relevant for investigating fibrosis progression in MASLD.

Next, serum levels of sol-MerTK, sol-Axl, and Gas6 were measured. In the male model, CDAHFD feeding significantly increased serum sol-MerTK, sol-Axl, and Gas6 levels at all time points compared with chow-fed controls (Fig. 3C–E). Within the CDAHFD time-course groups (4, 8, and 12 weeks), serum sol-MerTK levels progressively decreased, while serum sol-Axl and Gas6 levels increased during the feeding period (Fig. 3C–E). Serum sol-MerTK levels were significantly lower, and sol-

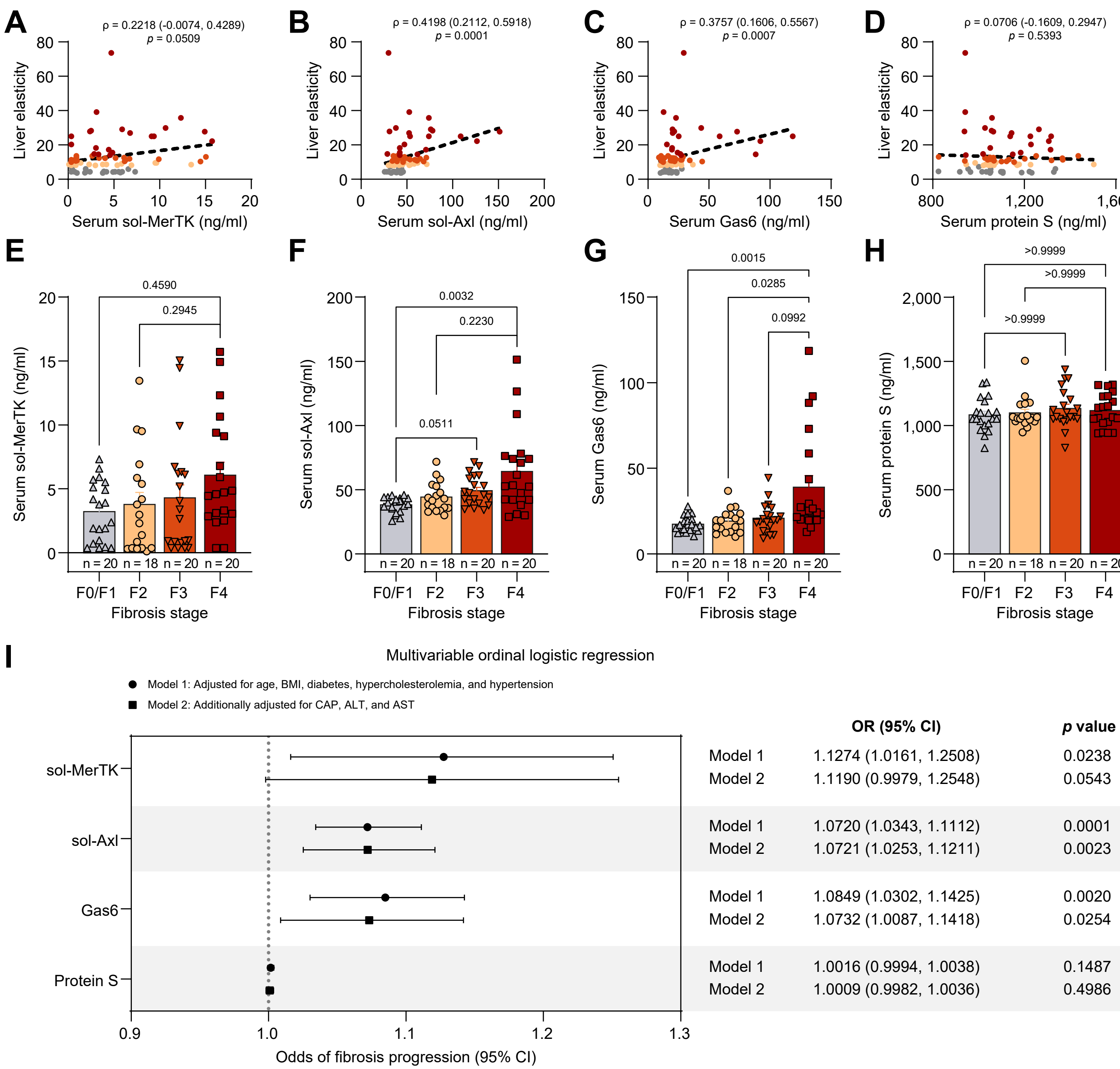

Fig. 2. Serum sol-MerTK and Protein S are not, but sol-Axl and Gas6 are, positively associated with liver fibrosis severity in women with MASLD. (A–D) Spearman's correlation analyses showing the correlations between liver elasticity (kPa) and serum sol-MerTK (A), sol-Axl (B), Gas6 (C), and Protein S (D) in women with MASLD (*n* = 78). (E–H) Serum levels of sol-MerTK (E), sol-Axl (F), Gas6 (G), and Protein S (H) in women with MASLD and different fibrosis stages (F0/1–F4; *n* = 18–20 per group). (I) Multivariable ordinal regression analysis showing the associations between liver fibrosis stage and serum levels of sol-MerTK, sol-Axl, Gas6, and Protein S. Model 1 was adjusted for age, BMI, diabetes, hypercholesterolemia, and hypertension; Model 2 was additionally adjusted for CAP, ALT, and AST. Data are shown as scatter plots with bars representing the mean ± SEM; Kruskal-Wallis with Dunn's post hoc test (Bonferroni corrected).

Axl and Gas6 levels were significantly higher in mice with intense perisinusoidal liver fibrosis (12 weeks) than in those with mild liver fibrosis (4 weeks; Fig. 3C–E). These results are consistent with the findings in men with MASLD. We also determined mRNA expression levels of hepatic *Mertk*, *Axl*, and *Gas6* in the male CDAHFD model. The results showed that the mRNA expression levels of hepatic *Mertk*, *Axl*, and *Gas6* were significantly higher in 4, 8, and 12-week CDAHFD-fed mice than in chow-fed controls (Supplemental Fig. S3A–C). Notably, hepatic *Mertk* and *Axl* mRNA levels did not differ between 4, 8, and 12 weeks of CDAHFD feeding, whereas hepatic *Gas6* mRNA levels increased over time (Supplemental Fig. S3A–C). These data suggest that CDAHFD-induced hepatic steatohepatitis may be associated with increased transcription of hepatic *Mertk*, *Axl*, and *Gas6* in males. Furthermore, liver fibrosis progression appears to be associated with decreased cleavage of MerTK but enhanced cleavage of Axl in male MASLD mice.

In the female CDAHFD model, serum levels and hepatic mRNA expression of Axl and Gas6 showed trends similar to

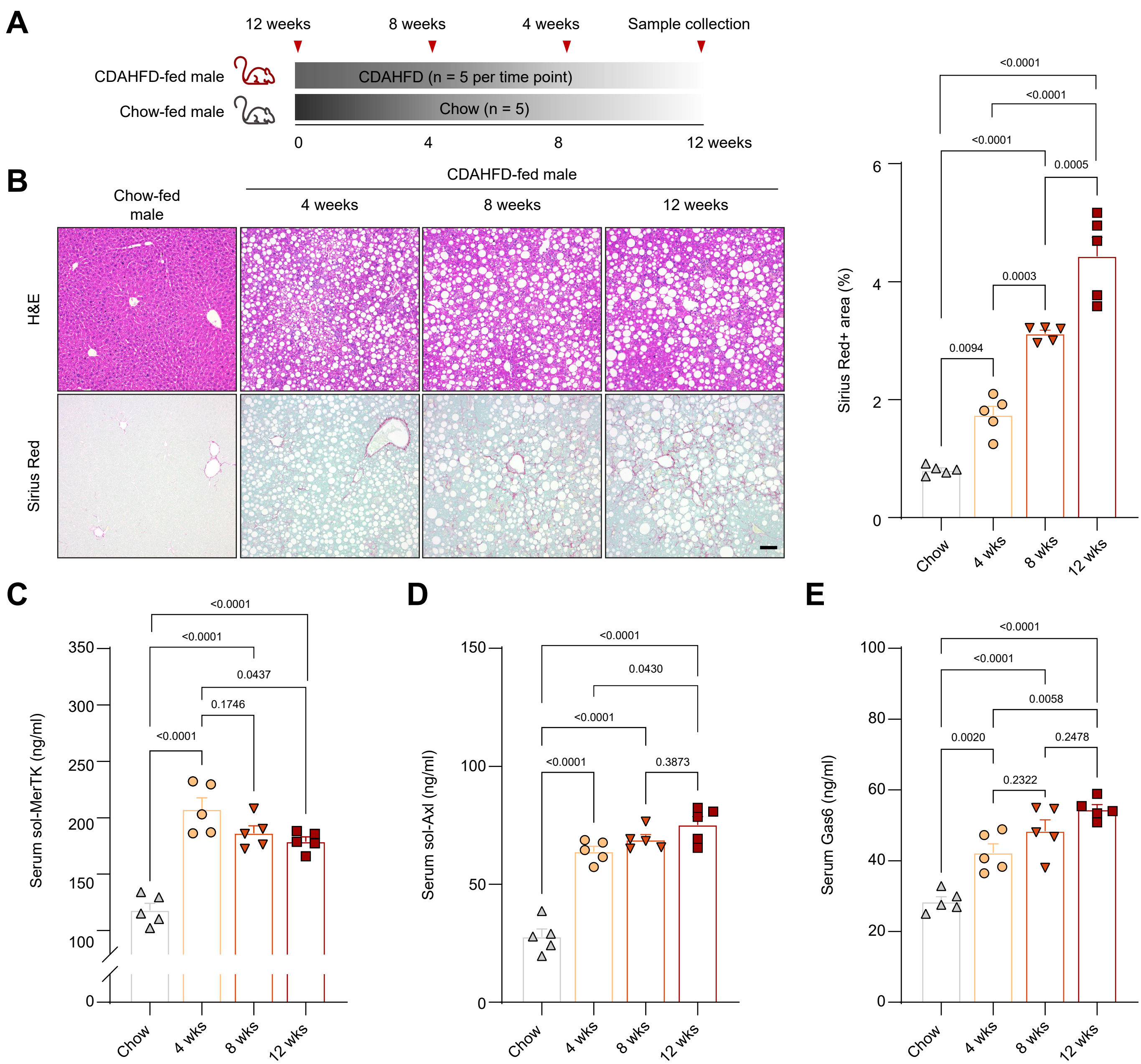

Fig. 3. Serum sol-MerTK, sol-Axl, and Gas6 levels are associated with MASLD development and fibrosis progression in CDAHFD-fed male mice. Male mice were fed a chow or CDAHFD diet for 4, 8, or 12 weeks (*n* = 5 per group). (A) Schematic diagram. (B) Representative H&E and Sirius red staining images (left) and quantification of percent Sirius red^{+} area (right). Scale bar, 100 µm. (C–E) Serum levels of sol-MerTK (C), sol-Axl (D), and Gas6 (E). Data are shown as scatter plots with bars representing the mean ± SEM; one-way ANOVA followed by Tukey's post hoc test.

those observed in males (Fig. 4D, E, and Supplemental Fig. S4B and C). Conversely, MerTK exhibited a sex-specific pattern. Unlike in males, serum sol-MerTK levels showed no significant differences among the 4, 8, and 12-week CDAHFD groups in females (Fig. 4C). Furthermore, hepatic *Mertk* mRNA levels did not differ significantly between chow-fed and CDAHFD-fed female mice, despite an upward trend (Supplemental Fig. S4A). Overall, these murine findings further support our clinical observations that the association between circulating sol-MerTK and fibrosis stages is sex-specific, whereas the associations for circulating sol-Axl and Gas6 remain consistent between sexes.

Serum sol-MerTK levels are lower in postmenopausal than in premenopausal women with MASLD

To determine whether menopausal status influenced serum levels of sol-MerTK, sol-Axl, Gas6, and Protein S, we compared their serum levels in premenopausal and postmenopausal women with MASLD. Among the 78 women in our main cohort, 16 were premenopausal, 46 were postmenopausal, and 16 with unknown menopausal status, as determined by self-report (Fig. 5A and Table 1). The percentage composition of premenopausal and postmenopausal women with different liver fibrosis stages in our main MASLD

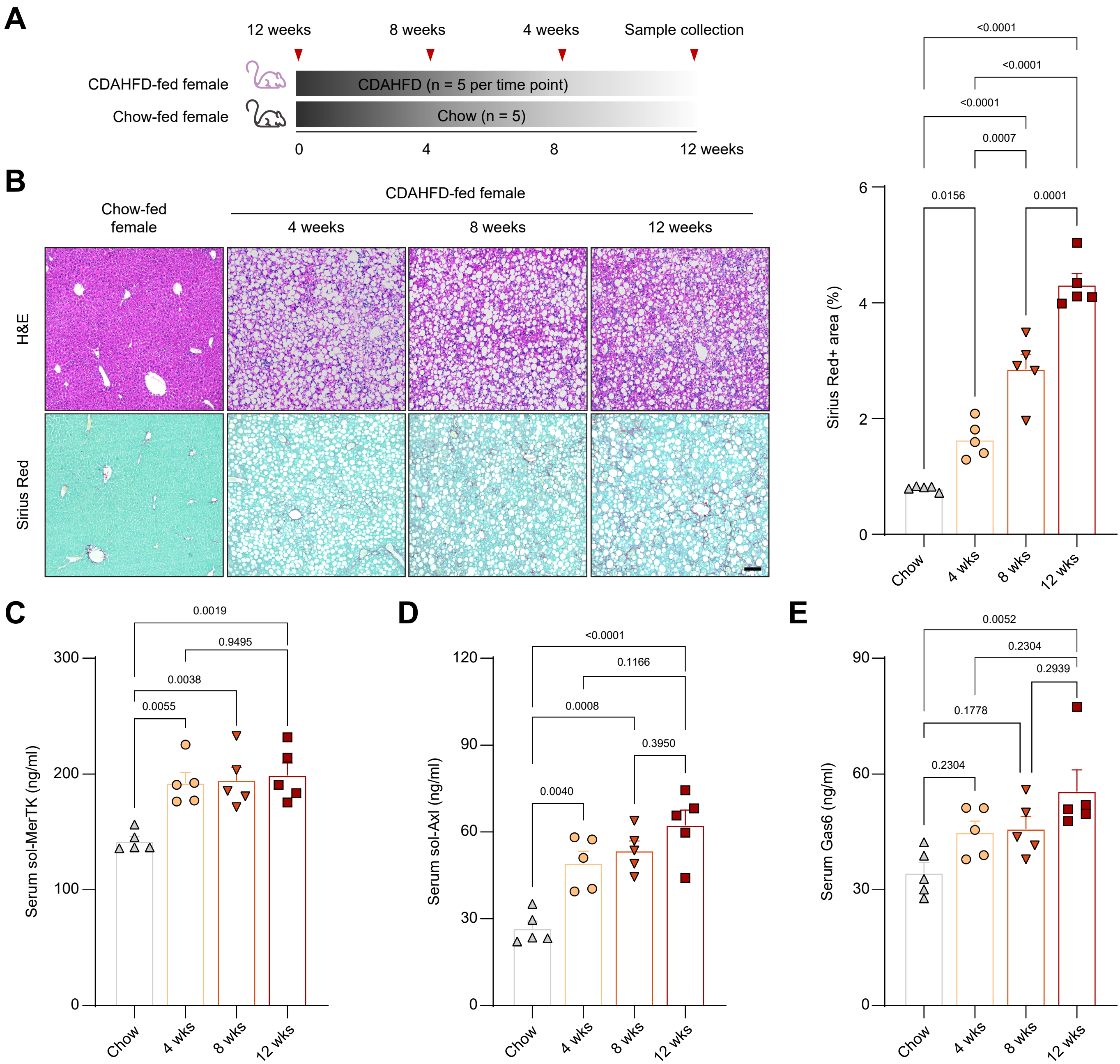

Fig. 4. The associations of serum sol-MerTK, sol-Axl, and Gas6 levels with liver fibrosis are characterized in CDAHFD-fed female mice. Female mice were fed a chow or CDAHFD diet for 4, 8, or 12 weeks (n = 5 per group). (A) Schematic diagram. (B) Representative H&E and Sirius red staining images (left) and quantification of percent Sirius red^{+} area (right). Scale bar, 100 μm. (C-E) Serum levels of sol-MerTK (C), sol-Axl (D), and Gas6 (E). Data are shown as scatter plots with bars representing the mean ± SEM; one-way ANOVA followed by Tukey's post hoc test.

cohort was shown in Figure 5B. Remarkably, we found that serum sol-MerTK levels were significantly lower in postmenopausal than in premenopausal group (Fig. 5C). However, no significant differences were observed in serum sol-Axl, Gas6, and Protein S levels between the postmenopausal and premenopausal groups (Fig. 5D–F). Given that four women had received hormone replacement therapy (HRT; Table 1), we conducted a sensitivity analysis excluding these cases. As shown in Supplemental Fig. S5, our sensitivity analysis results were consistent with the original findings. In fact, exclusion of these patients resulted in a more significant reduction in serum sol-MerTK levels (Supplemental Fig. S5A). These results suggest that menopause-related declines in estrogen may contribute to reduced circulating sol-MerTK.

To further validate whether estrogen affects circulating sol-MerTK, we measured serum sol-MerTK levels in male mice subcutaneously implanted with 17β-estradiol pellets, the prevalent endogenous estrogen in women.[26] The results showed that serum sol-MerTK levels were significantly higher in 17β-estradiol-administered mice than in control mice (Fig. 5G). These results support our proposed role of estrogen in increasing circulating sol-MerTK.

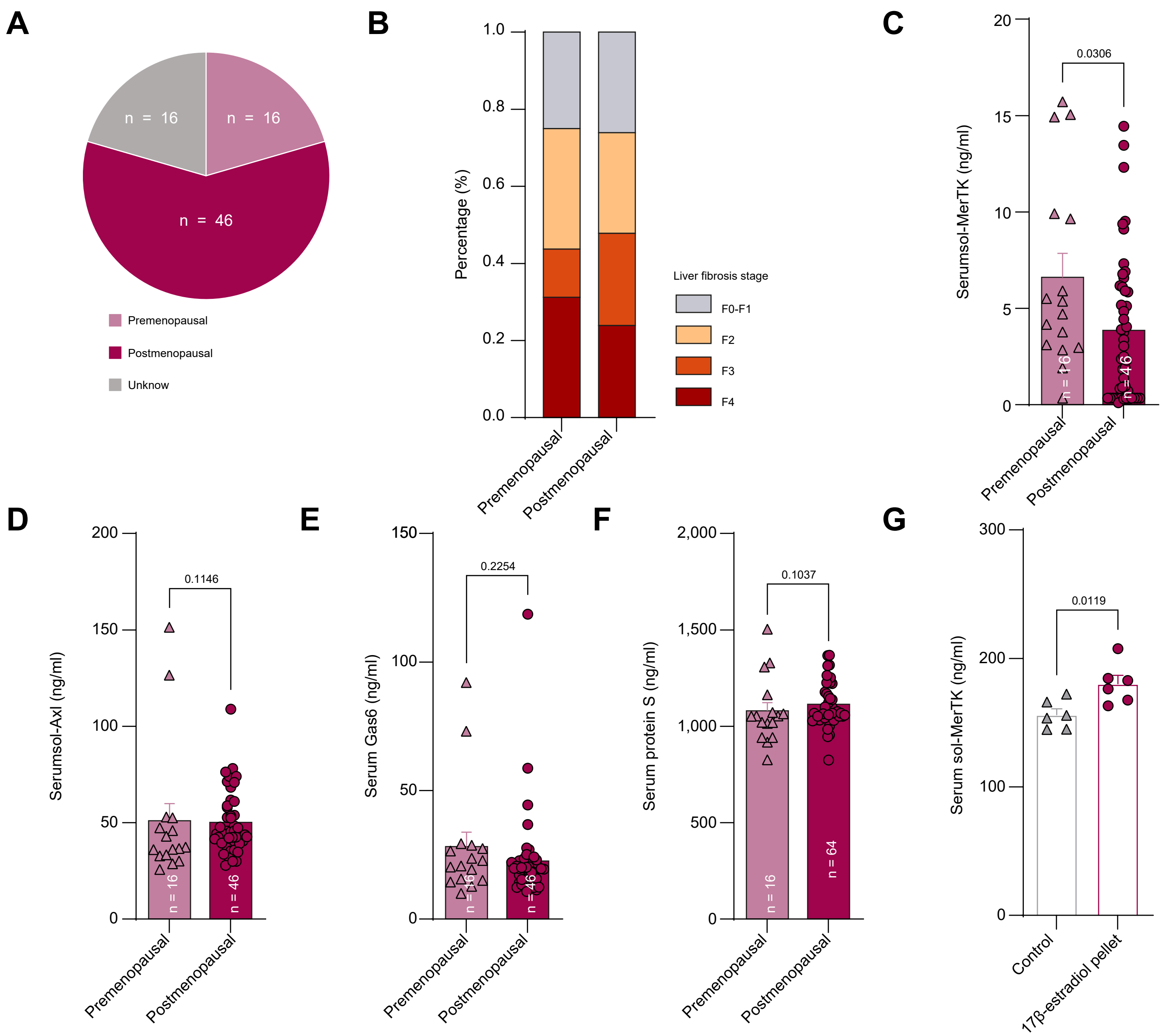

Fig. 5. Serum sol-MerTK levels are lower in postmenopausal than in premenopausal women with MASLD. (A) Pie chart showing menopausal status distribution in 78 women with MASLD, based on self-reported information. (B) Stacked bar plot showing the percentage composition of liver fibrosis stages (F0/1–F4) in premenopausal and postmenopausal women. (C–F) Serum levels of sol-MerTK (C), sol-Axl (D), Gas6 (E), and Protein S (F) in premenopausal (n = 16) and postmenopausal (n = 46) women with MASLD; two-tailed Mann-Whitney test. (G) Serum sol-MerTK levels in male mice implanted with subcutaneous 17β-estradiol pellets for 21 days (n = 6) or in control mice (n = 6); two-tailed unpaired t-test. Data are shown as scatter plots with bars representing the mean ± SEM.

Considering these findings, we excluded female patients with prior HRT and stratified the remaining patients by menopausal status to assess whether menopausal heterogeneity influences the relationship between circulating sol-MerTK and fibrosis severity in women with MASLD. As shown in Supplemental Fig. S6, no significant correlation between serum sol-MerTK and fibrosis severity was observed in either premenopausal (n = 16) or postmenopausal (n = 42) women with MASLD.

17β-estradiol induces MerTK cleavage in BMDMs

As sol-MerTK is primarily produced by the cleavage of the ectodomain of MerTK in macrophages,[27] we next sought to determine whether estrogen regulates MerTK cleavage in macrophages. We treated BMDMs with 17β-estradiol and found that 17β-estradiol treatment increased sol-MerTK levels in BMDM culture supernatants in a dose-dependent manner (Fig. 6A). Notably, 17β-estradiol treatment showed a non-significant trend toward reduced *Mertk* mRNA expression at the tested concentrations (Fig. 6B), suggesting that 17β-estradiol induces MerTK cleavage. These *in vitro* results are in line with our clinical observations that postmenopausal women with lower estrogen levels have reduced circulating sol-MerTK, and that 17β-estradiol administration increases circulating sol-MerTK in mice. Given that reduced MerTK cleavage has been shown to promote MASLD-related fibrosis in mice,[16] our data

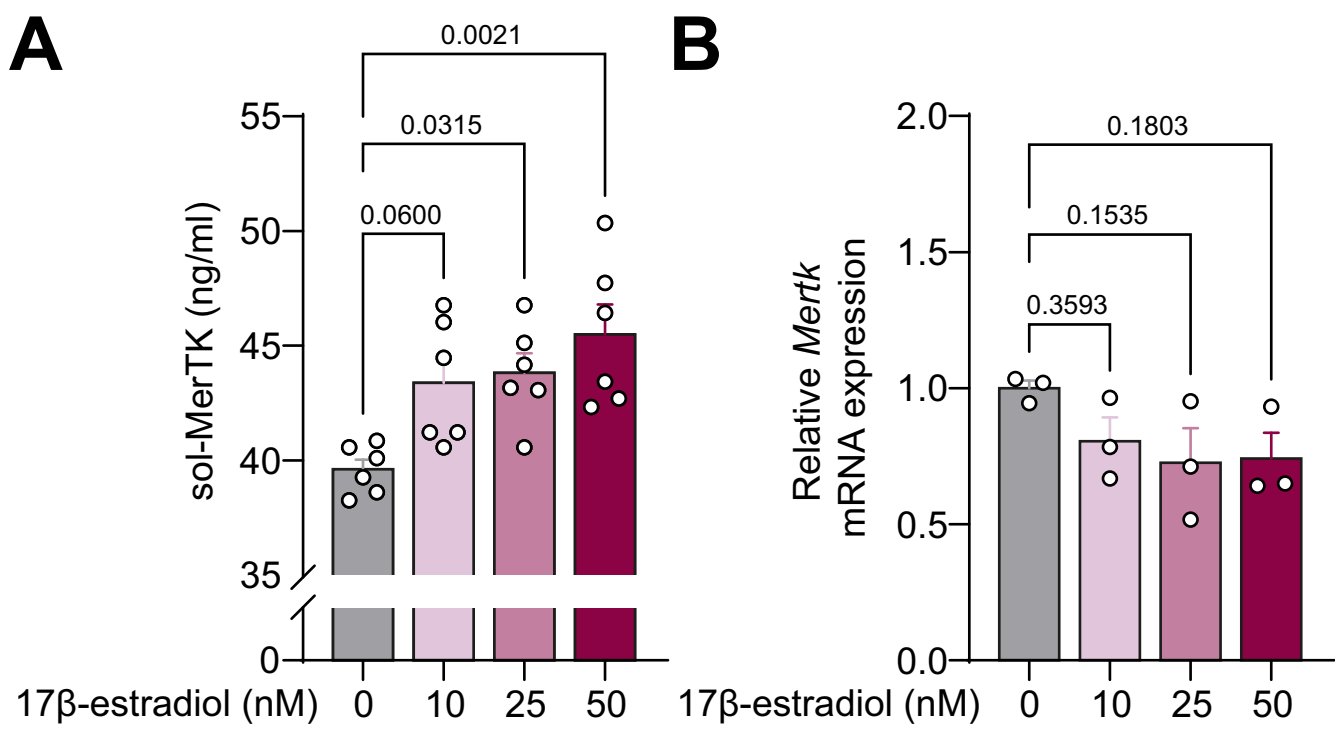

Fig. 6. 17β-estradiol induces MerTK cleavage in BMDMs. BMDMs were isolated from female mice and treated with different concentrations of 17β-estradiol for 24 h. (A) sol-MerTK levels in BMDM culture supernatants. (B) mRNA levels of *Mertk* in BMDMs. Data are shown as scatter plots with bars representing the mean ± SEM; one-way ANOVA followed by Tukey's post hoc test.

suggest that estrogen-induced MerTK cleavage may play a role in MASLD-related fibrosis.

Discussion

MASLD is a sex-dimorphic disease, with generally higher prevalence in men.[8,11,19] Emerging evidence suggests that sexual dimorphism in MASLD is underpinned by sex-specific modulations of insulin sensitivity, hepatic lipid homeostasis, immune responses, and fibrogenesis.[8,28,29] The TAM receptors MerTK and Axl together with their ligands Gas6 and Protein S play important roles in immune responses and fibrogenesis,[27,30,31] and have been implicated in the development of fibrosis in MASLD.[16,17] In this study, we for the first time analyzed circulating TAM receptors and their ligand levels separately in men and women with MASLD, with comparable numbers of patients of each sex at every liver fibrosis stage. Our findings revealed a striking sex-specific association, in that lower circulating sol-MerTK levels were associated with more advanced liver fibrosis in men with MASLD, whereas in women, sol-MerTK levels tended to increase with fibrosis progression but did not reach statistical significance. These observations were further supported by an independent validation cohort, although this cohort was limited by its small sample size and heterogeneous methods for fibrosis assessment. Given the established role of MerTK signaling in MASLD-related fibrogenesis,[16] our observations suggest that MerTK-related pathways may be involved in sex-specific patterns of fibrogenesis during MASLD progression. It should be noted that fibrosis staging in the Mount Sinai cohort was based on FibroScan-derived liver stiffness, which may be influenced by hepatic steatosis and necroinflammation in addition to fibrosis.[32] Nevertheless, additional adjustment for CAP score, ALT, and AST helped address this potential limitation. In contrast with our finding, previous studies have reported higher serum MerTK levels in patients with advanced versus early-stage liver fibrosis in their study cohorts.[18,19] This discrepancy might be partly explained by two potential confounding factors: sex composition and genetic background. First, previous studies did not analyze their data separately by sex or adjust for sex as a potential confounding factor. Given the opposite trends observed between sexes in our cohort, differences in the male-to-female ratio across study populations may skew the overall association. Second, our previous study demonstrated that the intronic *MERTK* rs4374383 variant is associated with *MERTK* expression.[16] As circulating sol-MerTK levels may reflect both MerTK cleavage and baseline *MERTK* expression, differences in the genetic background across cohorts may also contribute to the discrepant findings. However, genetic data were not available in our cohorts. Therefore, one limitation of our study is that we were unable to adjust for *MERTK* rs4374383 or other potential genetic confounders when assessing the sex-specific associations between circulating sol-MerTK and fibrosis severity. Notably, genetic risk variants may exert sex-specific effects on MASLD progression, as reported for PNPLA3 I148M and HSD17B13 rs72613567.[33,34] Sex-specific associations involving *MERTK*-related SNPs have also been reported in Alzheimer's disease.[35] In addition, *MERTK* expression differs by sex across multiple human tissues.[36] These findings highlight an important direction for future studies to determine whether the reported associations of the intronic MERTK SNPs with MERTK expression and liver fibrosis are sex dependent.

Sex differences across different experimental MASLD models may vary depending on the disease-inducing mechanisms and downstream pathways.[37] While young adult female mice generally exhibit lower susceptibility than age-matched males to disease phenotypes in most experimental MASLD models,[37–40] more severe hepatic pathology in females has also been reported in certain experimental settings.[41] Thus, the selection of experimental models for studies of sex differences in MASLD requires careful consideration. In this study, we sought to experimentally recapitulate the sex-specific patterns observed in our clinical cohorts using the CDAHFD model with different fibrosis severity, in which sex differences in fibrosis have been reported.[39] Similar to our clinical observations, the CDAHFD models showed sex-specific patterns in serum sol-MerTK levels, which progressively decreased as fibrosis advanced from 4 to 12 weeks in male mice but did not differ in female mice. However, it should be noted that our male and female mouse cohorts were established independently at different times and in different animal facilities, direct male versus female comparisons may not be appropriate, particularly given that environmental factors such as ambient temperature and microbiome can influence sex differences in MASLD susceptibility.[42] Moreover, our female CDAHFD experiments were conducted in young adult female mice. Future studies using experimental models that more closely recapitulate MASLD progression across different reproductive statuses will be needed.

MerTK cleavage is suppressed in fibrotic compared with steatotic livers from male MASLD mice.[16] In our male MASLD model, we observed that both hepatic *Mertk* mRNA and serum sol-MerTK levels were significantly higher in CDAHFD-fed male mice than in chow-fed controls. However, serum sol-MerTK levels but not hepatic *Mertk* mRNA levels were decreased as fibrosis progressed. These combined data raise the possibility that circulating sol-MerTK levels are initially increased in early MASLD due to enhanced *Mertk* transcription, whereas their subsequent decline in progressive fibrosis may reflect impaired cleavage in fibrotic livers. Therefore, the decreased MerTK cleavage may represent a key mechanism underlying

the progressive decline in circulating sol-MerTK levels observed in advancing liver fibrosis in males.

Postmenopausal women have a higher risk of MASLD and are more likely to develop advanced hepatic fibrosis than premenopausal women.[11] The postmenopausal decline in estrogen is considered to be a key factor contributing to the increased susceptibility to MASLD.[8] Our observation that circulating sol-MerTK levels were markedly lower in postmenopausal than in premenopausal women suggests that estrogen may regulate sol-MerTK production. Although this interpretation is limited by the small number of premenopausal women in our cohort, findings from our *in vivo* 17β-estradiol implantation mouse model support the likelihood that estrogen induces sol-MerTK production. Moreover, our *in vitro* experiments suggest that 17β-estradiol may promote sol-MerTK release from BMDMs in a dose-dependent manner, potentially through enhanced MerTK cleavage. It remains to be determined whether MerTK cleavage is specifically regulated by 17β-estradiol or more broadly by other estrogens, and whether this regulation occurs only in macrophages. Given that estrogen regulates MerTK cleavage in a dose-dependent manner, the observed non-association between circulating sol-MerTK and fibrosis severity in women with MASLD may be confounded by inter-individual variations in estrogen levels. However, estrogen data were not available in our cohort. We alternatively stratified women by menopausal status and observed no significant association between circulating sol-MerTK and fibrosis severity in either premenopausal or postmenopausal women. However, the premenopausal subgroup analysis was limited by the small sample size. Therefore, future studies in larger, well-characterized clinical cohorts with detailed reproductive status information and circulating estrogen measurements will be needed to clarify this relationship. Our previous study has demonstrated that MerTK cleavage plays a protective role against liver fibrosis in MASLD.[16] Thus, the estrogen–MerTK axis may represent one plausible mechanism underlying sex differences in MASLD-related fibrosis. However, further *in vivo* hormonal manipulation and loss-of-function experiments are required to determine whether this axis functionally contributes to sex differences in fibrosis progression in MASLD. Nevertheless, our results highlight the importance of considering sex differences in future investigations of MerTK-related studies in MASLD.

In summary, from a translational perspective, our study highlights the need to integrate sex as a core biological variable in the design of preclinical studies, clinical trials, and biomarker development strategies in MASLD. In recent years, sex disparities in MASLD trial enrollment have improved;[12] however, important gaps remain in sex-aware study design and sex-stratified analysis.[13] Our findings also support emerging recommendations to consider reproductive status as an additional layer of biological heterogeneity in study design and analysis. The enrollment of women of reproductive age in clinical trials is often subject to additional restrictions because of ethical and safety concerns.[13,43] These necessary safeguards may limit the representation of women across different reproductive and hormonal states, potentially overlooking biological differences that may affect therapeutic efficacy, drug metabolism, and safety profiles. Thus, future preclinical MASLD studies and clinical observational cohort studies should first adopt more rigorous sex-aware designs and incorporate reproductive status and hormonal exposure into both study design and analysis. In interventional trials, efforts should be made to improve representation of women across reproductive status whenever ethically justifiable and clinically safe. Moreover, sex- and reproductive status–stratified analyses and reporting are encouraged to improve the generalizability of trial findings and to support precision medicine in MASLD.

Affiliations

[1]*Vatche and Tamar Manoukian Division of Digestive Diseases, Department of Medicine, David Geffen School of Medicine, University of California, Los Angeles, CA, USA;* [2]*Comprehensive Liver Research Center at UCLA, University of California, Los Angeles, CA, USA;* [3]*Division of Liver Diseases, Department of Medicine, Icahn School of Medicine at Mount Sinai, New York, NY, USA;* [4]*Division of Endocrinology, Diabetes and Hypertension, Department of Medicine, David Geffen School of Medicine, University of California, Los Angeles, CA, USA*

Financial support

This work was supported in part by NIH grants R01DK134610, R01HL167107, and R35GM147269, the Irma T. Hirschl/Monique Weill-Caulier Trust Research Award (to B. Cai.); R01DK128289 (to S.L.F.); R01DK125354 (to Z.Z.); K08DK136706 (to A.N.); IK2CX002593 (to J.N.B.); UL1TR004419 (Clinical translational Science Award to Icahn School of Medicine at Mount Sinai); Mount Sinai MASH Center of Excellence (to M.B.B.).

Conflict of interest

The authors declare no conflicts of interest.

Please refer to the accompanying ICMJE disclosure forms for further details.

Authors' contributions

B. Cai., Y.Z., and N.G. designed the study; Y.Z. and N.G. performed experiments; B. Cogliati performed histological analysis in mouse models; M.B.B., S.L.F., X.Z., A.L., and Y.H. provided serum samples and clinical data from the Mount Sinai MASLD/MASH biobank; A.N., S.B.H., R.S., and J.N.B provided serum samples and clinical data from the UCLA CLRC Human MASLD Core; Z. Z. provided serum samples from the 17β-estradiol pellet implantation mouse model; Y.Z. and B. Cai. integrated and analyzed the data and drafted the manuscript; and all the coauthors participated in the manuscript editing.

Data availability

All data are available from the corresponding authors upon reasonable request.

Supplementary data

Supplementary data to this article can be found online at https://doi.org/10.1016/j.jhepr.2026.101937.

References

Author names in bold designate shared co-first authorship

[1] Noureddin M, Vipani A, Bresee C, et al. NASH leading cause of liver transplant in women: updated analysis of indications for liver transplant and ethnic and gender variances. Am J Gastroenterol 2018;113:1649–1659.

[2] Setiawan VW, Stram DO, Porcel J, et al. Prevalence of chronic liver disease and cirrhosis by underlying cause in understudied ethnic groups: the multiethnic cohort. Hepatology 2016;64:1969–1977.

[3] Zhu Y, Cai B. Mechanisms and therapeutic insights into MASH-associated fibrosis. Trends Endocrinol Metab 2026;37:402–417.

[4] Sanyal AJ, Natta MLV, Clark J, et al. Prospective study of outcomes in adults with nonalcoholic fatty liver disease. New Engl J Med 2021;385:1559–1569.

[5] **Hagström H**, **Nasr P**, Ekstedt M, et al. Fibrosis stage but not NASH predicts mortality and time to development of severe liver disease in biopsy-proven NAFLD. J Hepatol 2017;67:1265–1273.
[6] Noureddin M, Muthiah MD, Sanyal AJ. Drug discovery and treatment paradigms in nonalcoholic steatohepatitis. Endocrinol Diabetes Metab 2020;3:e00105.
[7] **Loomba R**, **Friedman SL**, **Shulman GI**. Mechanisms and disease consequences of nonalcoholic fatty liver disease. Cell 2021;184:2537–2564.
[8] Cherubini A, Della Torre S, Pelusi S, et al. Sexual dimorphism of metabolic dysfunction-associated steatotic liver disease. Trends Mol Med 2024;30:1126–1136.
[9] Younossi ZM, Golabi P, Paik JM, et al. The global epidemiology of nonalcoholic fatty liver disease (NAFLD) and nonalcoholic steatohepatitis (NASH): a systematic review. Hepatology 2023;77:1335–1347.
[10] Riazi K, Azhari H, Charette JH, et al. The prevalence and incidence of NAFLD worldwide: a systematic review and meta-analysis. Lancet Gastroenterol Hepatol 2022;7:851–861.
[11] DiStefano JK. NAFLD and NASH in postmenopausal women: implications for diagnosis and treatment. Endocrinology 2020;161.
[12] Souza M, Al-Sharif L, Diaz I, et al. Representation of sex, race and ethnicity in MASH randomised controlled trials: a systematic review and meta-analysis. Liver Int 2025;45:e70029.
[13] **Burra P**, **Zanetto A**, **Germani G**. Sex bias in clinical trials in gastroenterology and hepatology. Nat Rev Gastroenterol Hepatol 2022;19:413–414.
[14] **Im YR**, **Hunter H**, **de Gracia Hahn D**, et al. A systematic review of animal models of NAFLD finds high-fat, high-fructose diets most closely resemble human NAFLD. Hepatology 2021;74:1884–1901.
[15] **Vacca M**, **Kamzolas I**, **Harder LM**, et al. An unbiased ranking of murine dietary models based on their proximity to human metabolic dysfunction-associated steatotic liver disease (MASLD). Nat Metab 2024;6:1178–1196.
[16] Cai B, Dongiovanni P, Corey KE, et al. Macrophage MerTK promotes liver fibrosis in nonalcoholic steatohepatitis. Cell Metab 2020;31:406–421.
[17] Bárcena C, Stefanovic M, Tutusaus A, et al. Gas6/Axl pathway is activated in chronic liver disease and its targeting reduces fibrosis via hepatic stellate cell inactivation. J Hepatol 2015;63:670–678.
[18] Pan Z, El Sharkway R, Bayoumi A, et al. Inhibition of MERTK reduces organ fibrosis in mouse models of fibrotic disease. Sci Transl Med 2024;16:eadj0133.
[19] Tutusaus A, de Gregorio E, Cucarull B, et al. A functional role of GAS6/TAM in nonalcoholic steatohepatitis progression implicates AXL as therapeutic target. Cell Mol Gastroenterol Hepatol 2020;9:349–368.
[20] **Grøndal SM**, **Tutusaus A**, Boix L, et al. Dynamic changes in immune cell populations by AXL kinase targeting diminish liver inflammation and fibrosis in experimental MASH. Front Immunol 2024;15:1400553.
[21] Petta S, Valenti L, Marra F, et al. MERTK rs4374383 polymorphism affects the severity of fibrosis in non-alcoholic fatty liver disease. J Hepatol 2016;64:682–690.
[22] **Patin E**, **Kutalik Z**, Guergnon J, et al. Genome-wide association study identifies variants associated with progression of liver fibrosis from HCV infection. Gastroenterology 2012;143:1244–1252.e1212.
[23] Cavalli M, Pan G, Nord H, et al. Genetic prevention of hepatitis C virus-induced liver fibrosis by allele-specific downregulation of MERTK. Hepatol Res 2017;47:826–830.
[24] Zhang X, Sivakumar V, Paredes-Marin A, et al. Real-world comparison of steatosis-associated fibrosis estimator and fibrosis-4 scores for assessing metabolic dysfunction-associated steatotic liver disease-related fibrosis in a highly diverse urban metabolic dysfunction-associated steatotic liver disease population. Clin Gastroenterol Hepatol 2026;24(1):261–263.e3.
[25] Paredes-Marin A, Napoli J, Sivakumar V, et al. Identifying environmental factors associated with significant fibrosis in metabolic dysfunction-associated steatotic liver disease/steatohepatitis. Dig Dis Sci 2025;70:3658–3670.
[26] Rannevik G, Jeppsson S, Johnell O, et al. A longitudinal study of the perimenopausal transition: altered profiles of steroid and pituitary hormones, SHBG and bone mineral density. Maturitas 1995;21:103–113.
[27] Cai B, Thorp EB, Doran AC, et al. MerTK cleavage limits proresolving mediator biosynthesis and exacerbates tissue inflammation. Proc Natl Acad Sci U S A 2016;113:6526–6531.
[28] Lonardo A, Nascimbeni F, Ballestri S, et al. Sex differences in nonalcoholic fatty liver disease: state of the art and identification of research gaps. Hepatology 2019;70:1457–1469.
[29] Booijink R, Ramachandran P, Bansal R. Implications of innate immune sexual dimorphism for MASLD pathogenesis and treatment. Trends Pharmacol Sci 2024;45:614–627.
[30] Rothlin CV, Carrera-Silva EA, Bosurgi L, et al. TAM receptor signaling in immune homeostasis. Annu Rev Immunol 2015;33:355–391.
[31] van der Meer JHM, van der Poll T, van 't Veers C. TAM receptors, Gas6, and protein S: roles in inflammation and hemostasis. Blood 2014;123:2460–2469.
[32] EASL Clinical Practice Guidelines on non-invasive tests for evaluation of liver disease severity and prognosis - 2021 update. J Hepatol 2021;75:659–689.
[33] **Cherubini A**, **Ostadreza M**, Jamialahmadi O, et al. Interaction between estrogen receptor-α and PNPLA3 p.I148M variant drives fatty liver disease susceptibility in women. Nat Med 2023;29:2643–2655.
[34] Vilar-Gomez E, Pirola CJ, Sookoian S, et al. The protection conferred by HSD17B13 rs72613567 polymorphism on risk of steatohepatitis and fibrosis may Be limited to selected subgroups of patients with NAFLD. Clin Translational Gastroenterol 2021;12:e00400.
[35] Zhang L, He CH, Coffey S, et al. Single-cell transcriptomic atlas of Alzheimer's disease middle temporal gyrus reveals region, cell type and sex specificity of gene expression with novel genetic risk for MERTK in female. medRxiv [Preprint] 2023;23. 2023.02.18.23286037. https://doi.org/10.1101/2023.02.18.23286037.
[36] Liu S, Wu J, Yang D, et al. Big data analytics for MerTK genomics reveals its double-edged sword functions in human diseases. Redox Biol 2024;70:103061.
[37] **Smiriglia A**, **Lorito N**, Serra M, et al. Sex difference in liver diseases: how preclinical models help to dissect the sex-related mechanisms sustaining NAFLD and hepatocellular carcinoma. iScience 2023;26:108363.
[38] Meyer J, Teixeira AM, Richter S, et al. Sex differences in diet-induced MASLD - are female mice naturally protected? Front Endocrinol (Lausanne) 2025;16:1567573.
[39] Lee C, Kim J, Han J, et al. Formyl peptide receptor 2 determines sex-specific differences in the progression of nonalcoholic fatty liver disease and steatohepatitis. Nat Commun 2022;13:578.
[40] Manjarrés L, Xavier A, González L, et al. Sex differences in the relationship between body composition and MASLD progression in a murine model of metabolic syndrome. iScience 2025;28.
[41] Arivazhagan L, Delbare S, Wilson RA, et al. Sex differences in murine MASH induced by a fructose-palmitate-cholesterol-enriched diet. JHEP Rep 2025;7:101222.
[42] Giles DA, Moreno-Fernandez ME, Stankiewicz TE, et al. Thermoneutral housing exacerbates nonalcoholic fatty liver disease in mice and allows for sex-independent disease modeling. Nat Med 2017;23:829–838.
[43] Sarkar M, Kushner T. Metabolic dysfunction-associated steatotic liver disease and pregnancy. J Clin Invest 2025;135.

Keywords: MASH; MerTK; Sex differences; Menopause; Estrogen.
Received 1 December 2025; received in revised form 17 June 2026; accepted 22 June 2026; Available online 30 June 2026

Journal of Hepatology, Volume *8*

Supplemental information

Sexual dimorphism in the association of circulating TAM receptors–ligands with MASLD-related fibrosis

Yiwei Zhu, Neha Gupta, Bruno Cogliati, Yulu He, Asher Leviton, Jihane N. Benhammou, Steven-Huy B. Han, Zhenqi Zhou, Alexander Nguyen, Xiaotao Zhang, Rajat Singh, Scott L. Friedman, Meena B. Bansal, and Bishuang Cai

Supplementary data

Sexual dimorphism in the association of circulating TAM receptors–ligands with MASLD-related fibrosis

Yiwei Zhu, Neha Gupta, Bruno Cogliati, Yulu He, Asher Leviton, Jihane N. Benhammou, Steven-Huy B. Han, Zhenqi Zhou, Alexander Nguyen, Xiaotao Zhang, Rajat Singh, Scott L. Friedman, Meena B. Bansal, Bishuang Cai

Table of contents

Supplementary Material and Methods..2

Supplementary Figures...4

Supplementary Tables..10

Supplementary References...14

Supplementary Material and Methods

Mouse liver histology and scoring

Hematoxylin and eosin (H&E) and picrosirius red staining were performed on formalin-fixed, paraffin-embedded (FFPE) sections. Briefly, fresh liver tissue was fixed in 10% neutral buffered formalin overnight and processed for paraffin embedding. FFPE blocks were sectioned at 3–5 µm using a microtome, and sections were mounted onto charged glass slides. Slides were deparaffinized in xylene and rehydrated through a graded ethanol series (100%, 95%, 85%, and 70% ethanol in water). After deparaffinization and rehydration, standard H&E staining was performed, and liver histology was evaluated by a veterinary pathologist blinded to group allocation. Lesions were graded according to the rodent MASLD scoring framework.[1] A global steatosis index was generated for each animal by summing the individual grades for macrovesicular steatosis, microvesicular steatosis, and hepatocellular hypertrophy. Each component was scored on a 0–3 scale, reflecting involvement of <5%, 5–33%, 33–66%, or >66% of the parenchyma, respectively. Lobular inflammatory activity was evaluated by quantifying inflammatory foci per field and assigning severity categories as follows: 0 (<0.5 foci/field), 1 (0.5–1.0 foci/field), 2 (1.0–2.0 foci/field), or 3 (>2.0 foci/field).

For picrosirius red staining, deparaffinized and dehydrated FFPE sections were first incubated for 2 hours in a solution containing 0.01% fast green (Sigma, #F7258) in picric acid (Sigma, #P6744), followed by an additional 2 hours in a solution containing 0.04% fast green and 0.1% sirius red (Sigma, #365548) in picric acid. Slides were then rehydrated and mounted in Permount solution (Fisher, #SP15) for microscopic evaluation. Liver fibrosis was quantified as the percentage of sirius red$^+$ area out of the total area using Fiji software.[2] Quantification was performed on the whole section for all imaging analyses.

ELISA measurement

Levels of sol-MerTK, sol-Axl, Gas6, and Protein S in human serum, mouse serum, and culture supernatants were measured using ELISA kits according to the manufacturers' instructions. Human serum samples were measured using the human ELISA kits (R&D Systems: #DY6488 for sol-MerTK, #DY154 for sol-Axl, and #DY885B for Gas6; Invitrogen: #EEL043 for Protein S). Mouse serum and cell culture supernatants were measured using the mouse ELISA kits (R&D

Systems: #DY591 for sol-MerTK, #DY854 for sol-Axl, and #DY986 for Gas6). Values below the lower limit of detection (LOD) were imputed as one-half of the LOD for statistical analysis.

Quantitative RT-PCR

Total RNA was extracted from mouse liver tissue or cultured cells using the RNeasy Mini Kit (Qiagen, #74106). Reverse transcription was performed with 0.5–1 μg RNA using the High-Capacity cDNA Reverse Transcription kit (Applied Biosystems, #4374967). Quantitative RT-PCR was performed in a volume of 10 μL using iQ SYBR Green Supermix (Bio-Rad, #1708886) and the Light Cycler 480 II Real-Time PCR system (Roche). Primer sequences are shown in Supplementary Table S1.

Isolation and treatment of mouse bone marrow-derived macrophages (BMDMs)

Mouse BMDMs were isolated from 8-to-10-week-old female C57BL/6J mice. Briefly, the mice were euthanized, and the skin was incised to expose the hind limbs. The femur and tibia were dissected by cutting through the hip and ankle joints, and bone marrow was flushed using a syringe fitted with a 26-gauge needle containing BMDM medium. The resulting bone marrow cell suspension was filtered through a 40-μm cell strainer and cultured for 7–10 days in DMEM supplemented with 10% (vol/vol) FBS, 1% penicillin–streptomycin, and 20% (vol/vol) L-929 fibroblast-conditioned medium.

To examine the effect of 17β-estradiol on MerTK *in vitro*, BMDMs were treated with DMSO or 17β-estradiol (10, 25, or 50 nM) for 24 h. Culture supernatants were collected to measure sol-MerTK, and the cells were harvested for RNA extraction.

Supplementary Figures

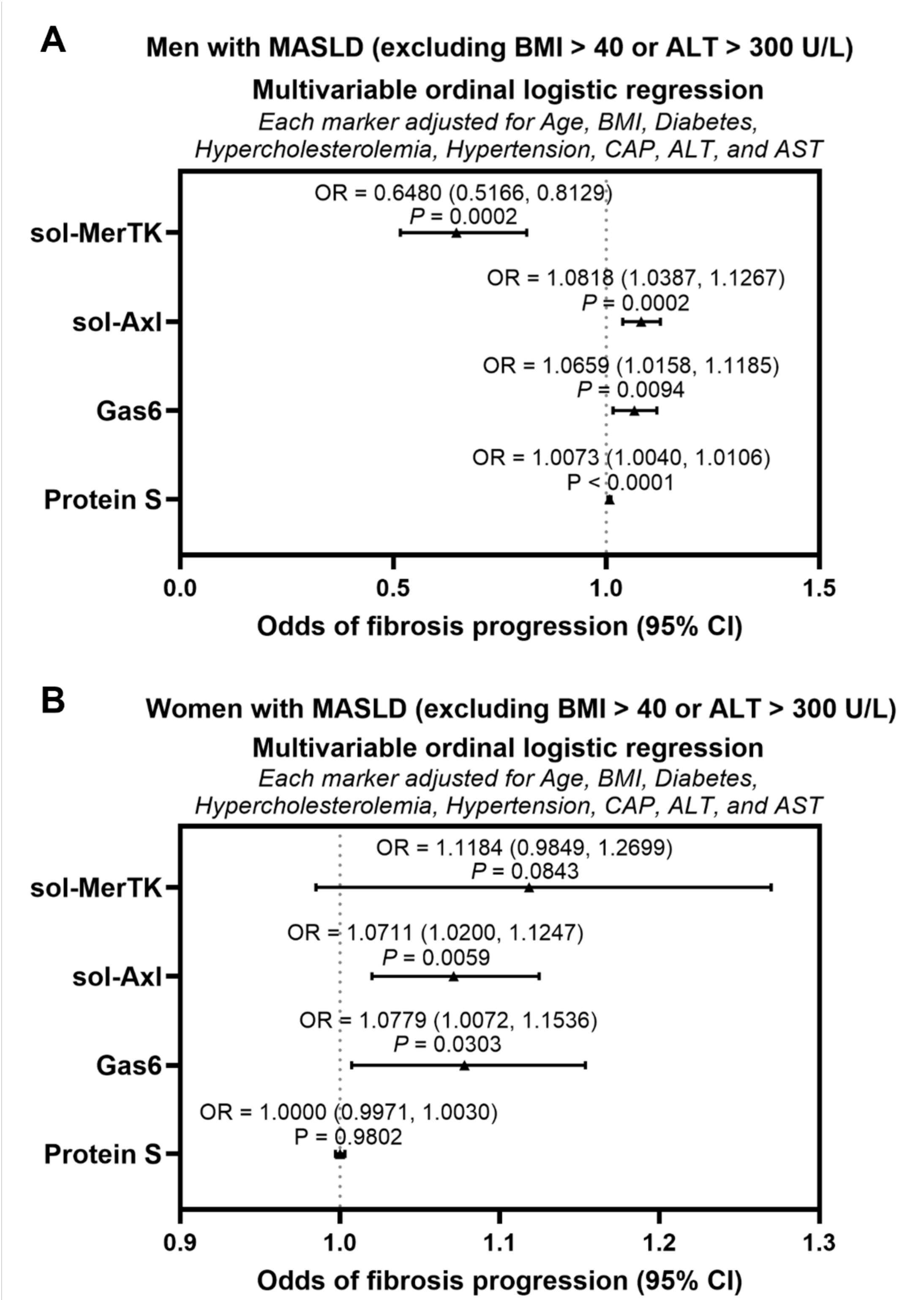

Fig S1. Sensitivity analysis excluding patients with BMI > 40 or ALT > 300 U/L. (A–B) Multivariable ordinal regression analysis showing the associations between liver fibrosis stage

and serum levels of sol-MerTK, sol-Axl, Gas6, and Protein S in men (A) and women (B) with MASLD.

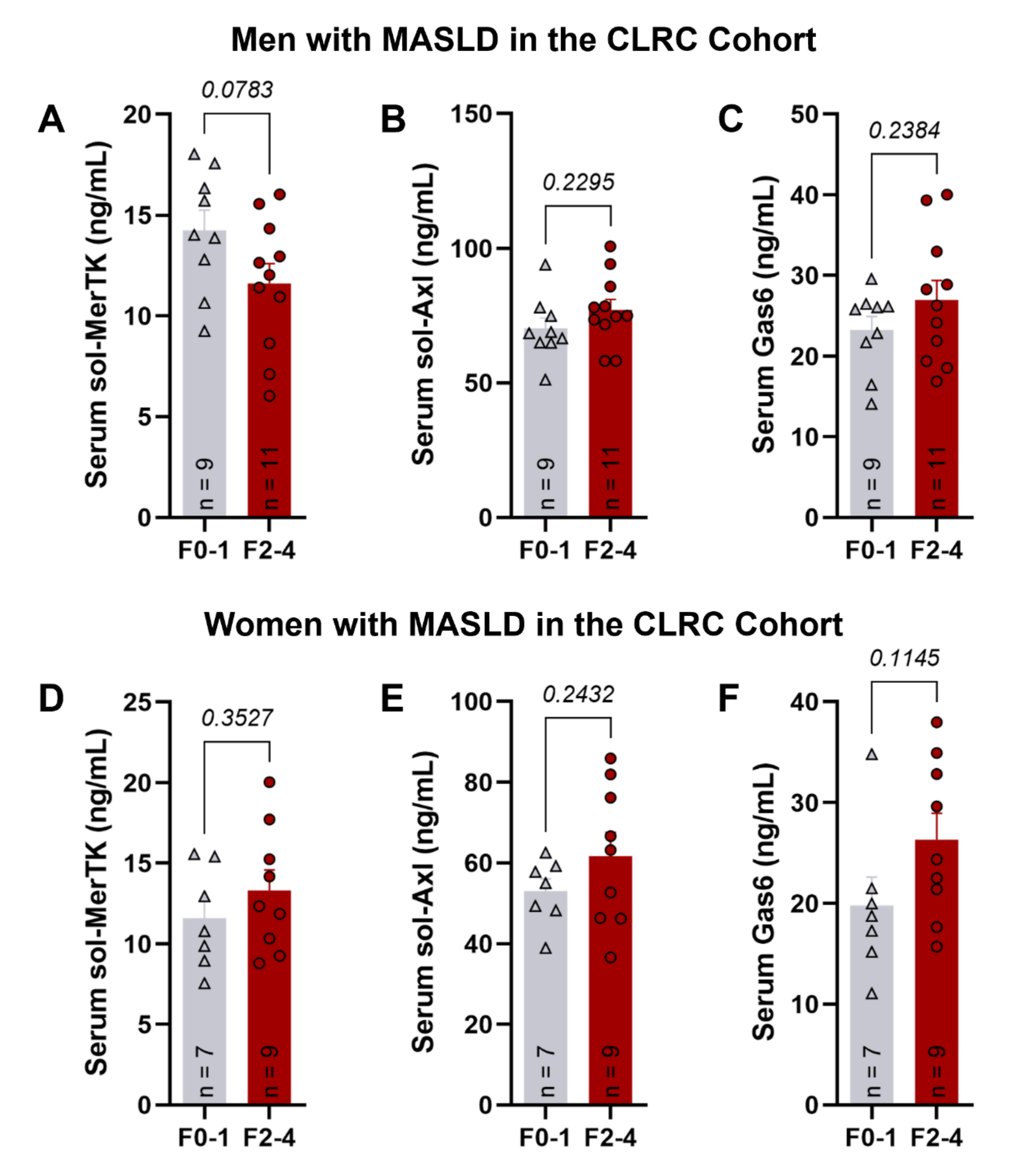

Fig S2. Serum sol-MerTK, sol-Axl, and **Gas6 levels in the CLRC cohort.** (A–C) Serum levels of sol-MerTK (A), sol-Axl (B), and Gas6 (C) in men with MASLD and either F0–1 or F2–4 fibrosis. (D–F) Serum levels of sol-MerTK (D), sol-Axl (E), and Gas6 (F) in women with MASLD and either F0–1 or F2–4 fibrosis. Data are shown as scatter plots with bars representing the mean ± SEM; two-tailed unpaired t test.

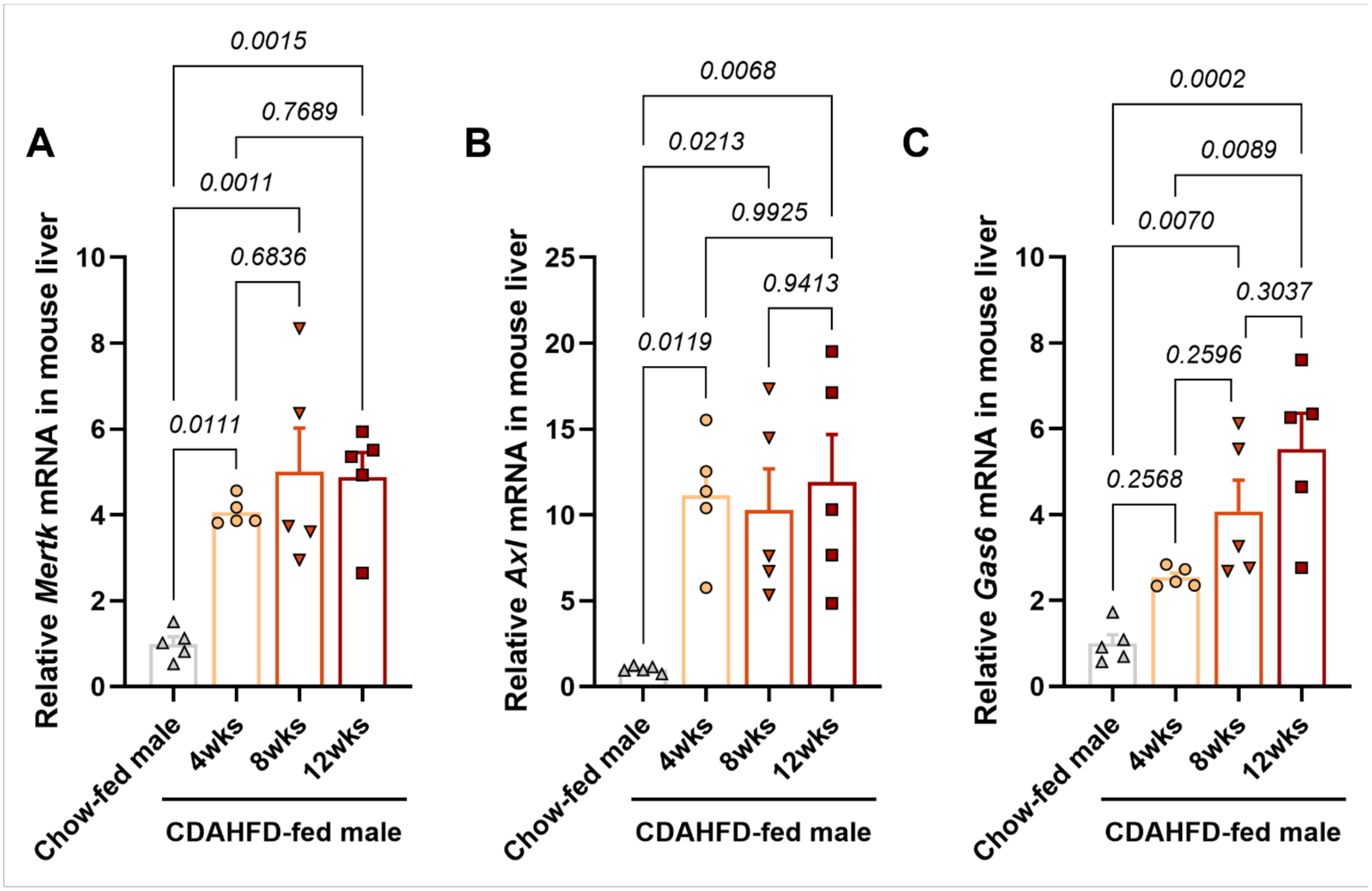

Fig S3. mRNA levels of hepatic *Mertk*, *Axl*, and *Gas6* in the male CDAHFD model. (A–C) mRNA levels of hepatic *Mertk* (A), *Axl* (B), and *Gas6* (C) in chow- or CDAHFD-fed male mice (*n* = 5 per group). Data are shown as scatter plots with bars representing the mean ± SEM; one-way ANOVA followed by Tukey's post hoc test.

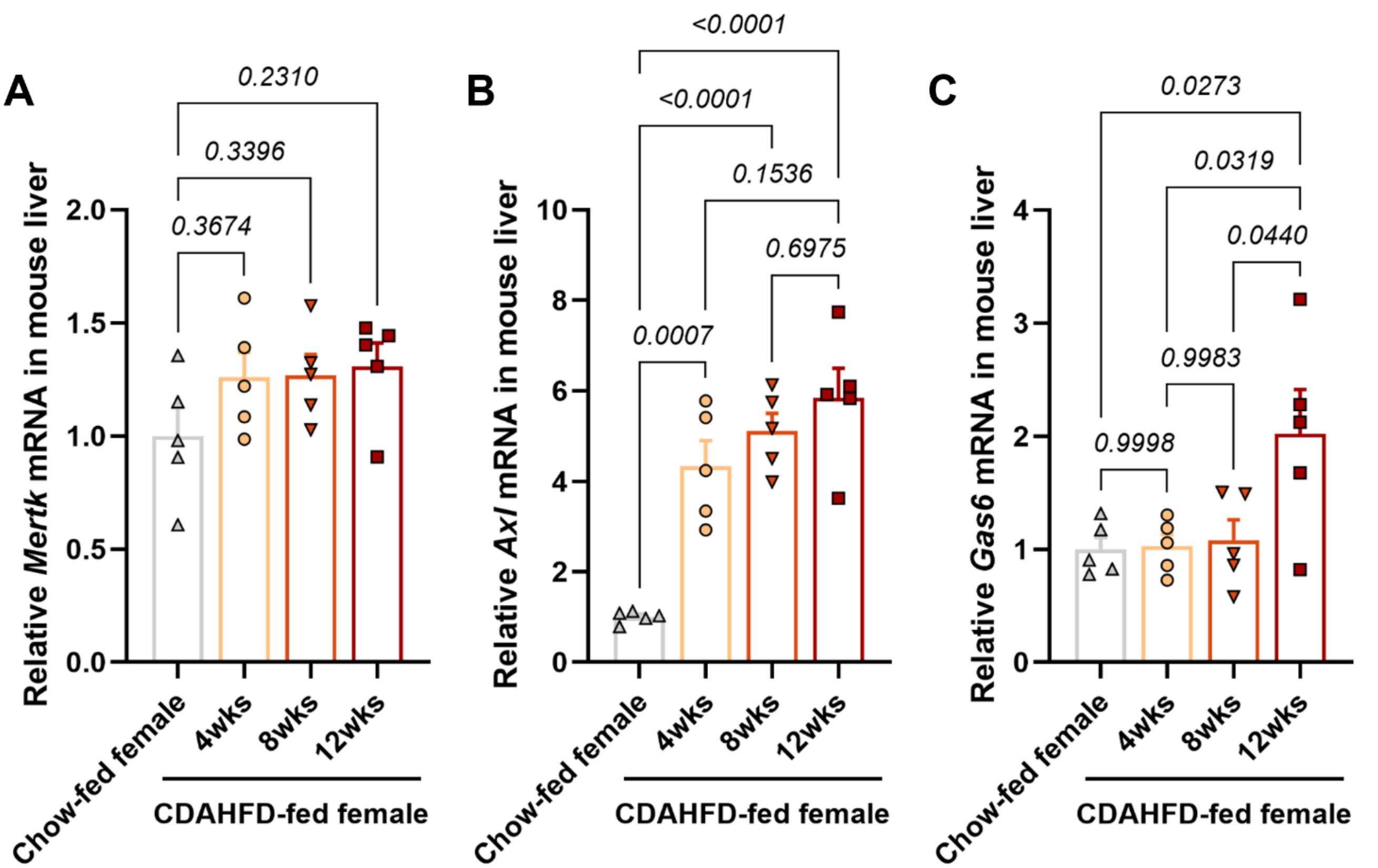

Fig S4. mRNA levels of hepatic *Mertk*, *Axl*, and *Gas6* in the female CDAHFD model. (A–C) mRNA levels of hepatic *Mertk* (A), *Axl* (B), and *Gas6* (C) in chow- or CDAHFD-fed female mice (*n* = 5 per group). Data are shown as scatter plots with bars representing the mean ± SEM; one-way ANOVA followed by Tukey's post hoc test.

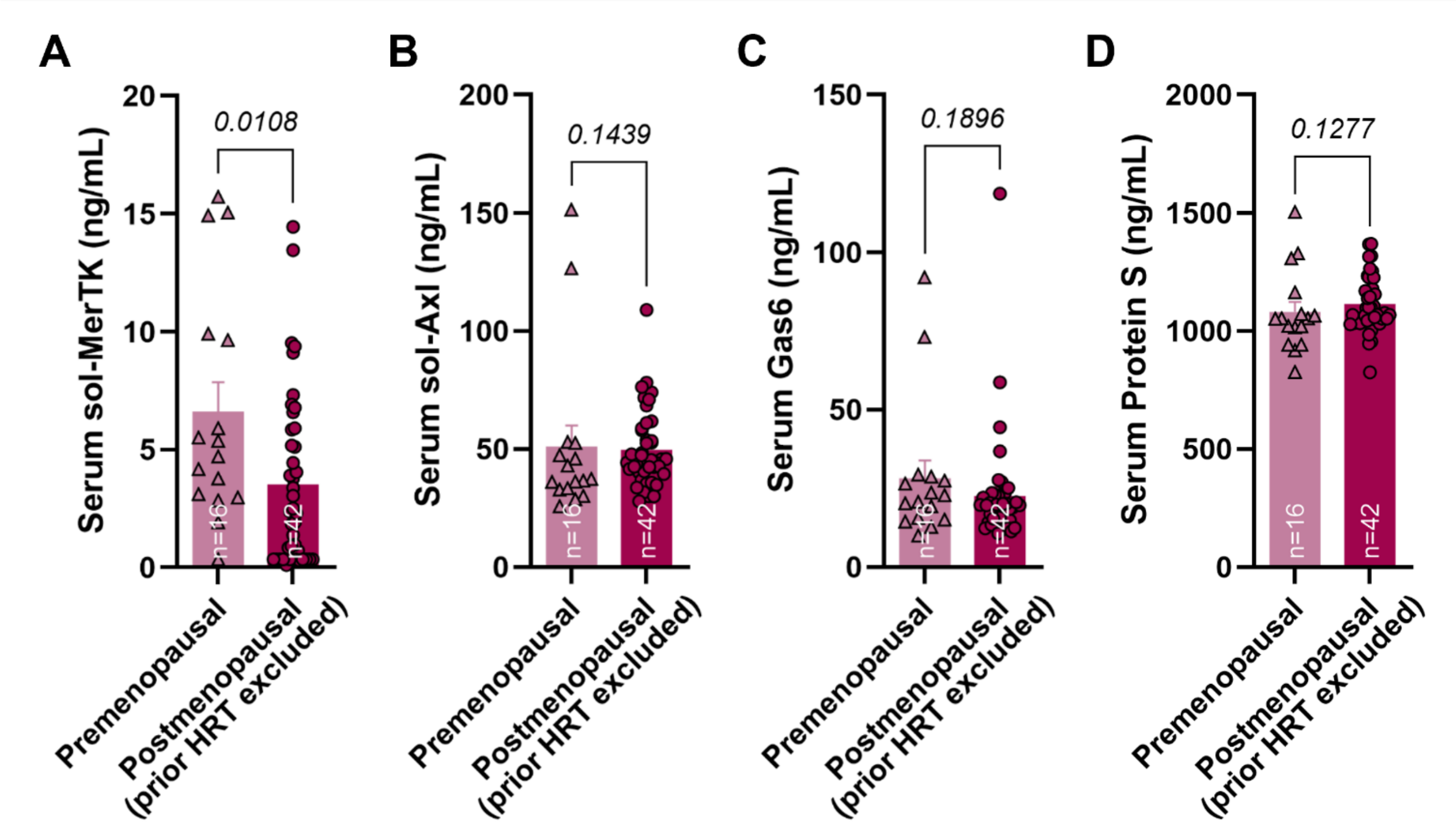

Fig S5. Sensitivity analysis excluding women who had received hormone replacement therapy. (A–D) Serum levels of sol-MerTK (A), sol-Axl (B), Gas6 (C), and Protein S (D) in premenopausal (*n* = 16) and postmenopausal (*n* = 42) women with MASLD. Data are shown as scatter plots with bars representing the mean ± SEM; two-tailed Mann-Whitney test.

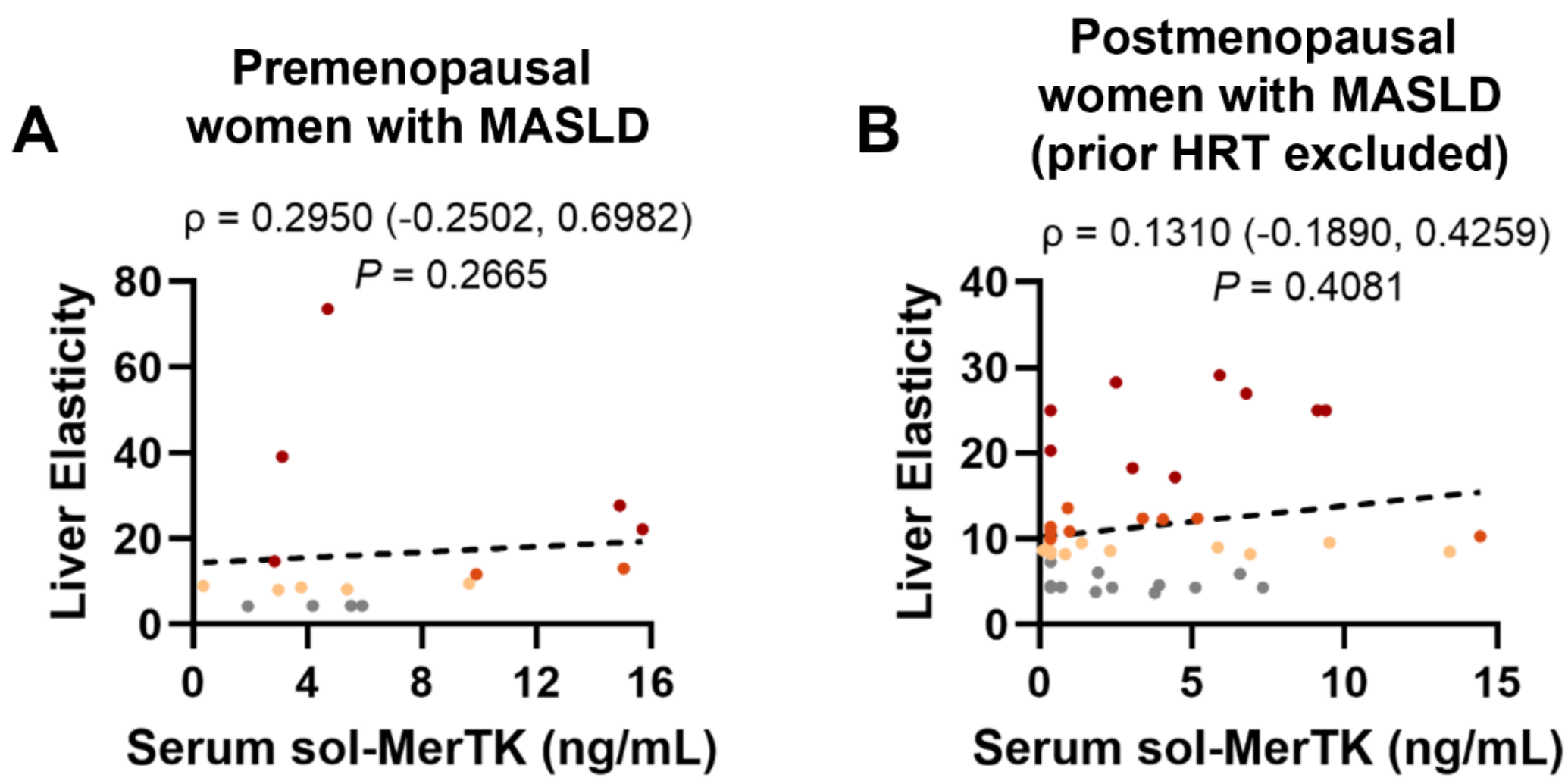

Fig S6. Association between circulating sol-MerTK and liver stiffness in premenopausal and postmenopausal women with MASLD. (A–B) Spearman's correlation between serum sol-MerTK and liver elasticity (kPa) in premenopausal (A; *n* = 16) and postmenopausal (B; *n* = 42) women with MASLD excluding who had received hormone replacement therapy.

Supplementary Tables

Table S1. Primers used for qPCR

Gene	Organism	Forward (5' - 3')	Reverse (5' - 3')
Mertk	Mouse	CAGGGCCTTTACCAGGGAGA	TGTGTGCTGGATGTGATCTTC
Axl	Mouse	ATGGCCGACATTGCCAGTG	CGGTAGTAATCCCCGTTGTAGA
Gas6	Mouse	TGCTGGCTTCCGAGTCTTC	CGGGGTCGTTCTCGAACAC

Mertk, MER proto-oncogene tyrosine kinase; Axl, AXL receptor tyrosine kinase; Gas6, growth arrest–specific 6.

Table S2. Baseline characteristics by sex and fibrosis severity in the CLRC Cohort

	**Male (*n* = 20)**		**Female (*n* = 16)**	
	**F0–F1 (*n* = 9)**	**F2–F4 (*n* = 11)**	**F0–F1 (*n* = 7)**	**F2–F4 (*n* = 9)**
**Fibrosis diagnosis, *n* (Stage)**	1 (F0), 6 (F0–F1), 2 (F1)	4 (F2), 3 (F2–3), 2 (F3), 2 (F4)	7 (F0–F1)	2 (F2–F3), 3 (F3), 2 (F3–F4), 2 (F4)
FibroScan	6 (F0–F1)	2 (F2), 2 (F3), 1 (F4)	5 (F0–F1)	1 (F3), 1 (F4)
Liver Biopsy	1 (F0), 2 (F1)	2 (F2), 2 (F2–3), 1 (F4)	-	2 (F2–F3), 2 (F3), 2 (F3–F4)
MRE	-	1 (F2–F3)	2 (F0–F1)	1 (F4)
**Continuous variables, mean (SD)**				
Age, years	45.6 (17.5)	59.4 (15.0)	58.1 (11.3)	55.9 (17.0)
BMI, kg/m^2	30.7 (3.5)	31.1 (4.4)	29.7 (7.9)	32.0 (6.1)
ALT, U/L	71.4 (57.0)	77.6 (56.5)	33.9 (14.7)	67.4 (37.3)
AST, U/L	44.6 (34.0)	50.6 (26.6)	28 (7.0)	50.4 (20.8)
**Comorbidities, *n* (%)**				
Obesity				
Yes	6 (66.7%)	7 (63.6%)	3 (42.9%)	3 (33.3%)
No	3 (33.3%)	4 (36.4%)	4 (57.1%)	6 (66.7%)
Diabetes				
Yes	3 (33.3%)	6 (54.5%)	5 (71.4%)	7 (77.8%)
No	6 (66.7%)	5 (45.5%)	2 (28.6%)	2 (22.2%)
Hypertension				
Yes	5 (55.6%)	8 (72.7%)	5 (71.4%)	6 (66.7%)
No	4 (44.4%)	3 (27.3%)	2 (28.6%)	3 (33.3%)
Hypercholesterolemia				
Yes	6 (66.7%)	8 (72.7%)	5 (71.4%)	6 (66.7%)
No	3 (33.3%)	3 (27.3%)	2 (28.6%)	3 (33.3%)
Kidney disease				
Yes	0 (0.0%)	1 (9.1%)	0 (0.0%)	2 (22.2%)
No	9 (100.0%)	10 (90.9%)	7 (100.0%)	7 (77.8%)
**Ethnicity, *n* (%)**				
Hispanic				
Yes	1 (11.1%)	1 (9.1%)	2 (28.6%)	0 (0.0%)
No	8 (88.9%)	9 (81.8%)	4 (57.1%)	7 (77.8%)

Note: Missing values for continuous variables were as follows: Age, BMI, ALT, and AST had 0% missing. Missing values for categorical variables were as follows: obesity, diabetes, hypertension, hypercholesterolemia, and kidney disease had 0% missing; ethnicity 4 (11.1%).

Table S3. Liver histology scores in Chow- and CDAHFD-fed male mice

Male	Macrovesicular steatosis (0-3)	Microvesicular steatosis (0-3)	Hepatocellular hypertrophy (0-3)	Steatosis Score (0-3)	Inflammatory foci (0-3)	Observations
**Chow**						
**1**	0	0	0	0	0	No fibrosis
**2**	0	0	0	0	0	No fibrosis
**3**	0	0	0	0	0	No fibrosis
**4**	0	0	0	0	0	No fibrosis
**5**	0	0	0	0	0	No fibrosis
**4 weeks of CDAHFD**						
**6**	2	3	3	8	3	Very mild perisinusoidal fibrosis
**7**	3	2	3	8	3	Very mild perisinusoidal fibrosis
**8**	3	2	3	8	3	Very mild perisinusoidal fibrosis
**9**	2	3	2	7	3	Very mild perisinusoidal fibrosis
**10**	3	2	3	8	3	Very mild perisinusoidal fibrosis
**8 weeks of CDAHFD**						
**11**	3	1	3	7	3	Moderate perisinusoidal fibrosis
**12**	3	1	3	7	3	Moderate perisinusoidal fibrosis
**13**	3	1	3	7	3	Moderate perisinusoidal fibrosis
**14**	3	2	3	8	3	Moderate perisinusoidal fibrosis
**15**	3	1	3	7	3	Moderate perisinusoidal fibrosis
**12 weeks of CDAHFD**						
**16**	3	2	3	8	3	Intense perisinusoidal fibrosis
**17**	3	1	3	7	3	Intense perisinusoidal fibrosis
**18**	3	1	3	7	3	Intense perisinusoidal fibrosis
**19**	3	1	3	8	3	Intense perisinusoidal fibrosis
**20**	3	1	3	7	3	Intense perisinusoidal fibrosis

Table S4. Liver histology scores in Chow- and CDAHFD-fed female mice

Female	Macrovesicular steatosis (0-3)	Microvesicular steatosis (0-3)	Hepatocellular hypertrophy (0-3)	Steatosis Score (0-3)	Inflammatory foci (0-3)	Observations
**Chow**						
**1**	0	0	0	0	0	No fibrosis
**2**	0	0	0	0	0	No fibrosis
**3**	0	0	0	0	0	No fibrosis
**4**	0	0	0	0	0	No fibrosis
**5**	0	0	0	0	0	No fibrosis
**4 weeks of CDAHFD**						
**6**	3	0	3	6	3	Very mild perisinusoidal fibrosis
**7**	3	1	3	7	3	Very mild perisinusoidal fibrosis
**8**	3	1	3	7	3	Very mild perisinusoidal fibrosis
**9**	2	1	3	7	3	Very mild perisinusoidal fibrosis
**10**	3	1	3	7	3	Very mild perisinusoidal fibrosis
**8 weeks of CDAHFD**						
**11**	3	0	3	6	2	Moderate perisinusoidal fibrosis
**12**	3	1	3	7	3	Moderate perisinusoidal fibrosis
**13**	3	0	3	6	2	Mild perisinusoidal fibrosis
**14**	3	1	3	7	3	Moderate perisinusoidal fibrosis
**15**	3	1	3	7	3	Moderate perisinusoidal fibrosis
**12 weeks of CDAHFD**						
**16**	3	2	3	8	3	Intense perisinusoidal fibrosis
**17**	3	1	3	7	3	Intense perisinusoidal fibrosis
**18**	3	1	3	7	3	Intense perisinusoidal fibrosis
**19**	3	1	3	7	3	Intense perisinusoidal fibrosis
**20**	3	1	3	7	3	Intense perisinusoidal fibrosis

Supplementary References

[1] Liang W, Menke AL, Driessen A, et al. Establishment of a general NAFLD scoring system for rodent models and comparison to human liver pathology. PLoS One. 2014;9:e115922.

[2] Schindelin J, Arganda-Carreras I, Frise E, et al. Fiji: an open-source platform for biological-image analysis. Nat Methods. 2012;9:676-682.
